# Synthesis of Novel Acetylene-Containing Phosphonates, Their Antiviral Activity, and Their Cytotoxicity to Different Cancer Cell Lines

**DOI:** 10.3390/molecules31111861

**Published:** 2026-05-28

**Authors:** Anastasia V. Egorova, Anastasia M. Lobova, Dmitrii M. Egorov, Elizaveta A. Tishchenko, Alexandrina S. Volobueva, Iana L. Esaulkova, Renata A. Kadyrova, Dar’ya V. Spiridonova, Andrew S. Drachuk, Vitali M. Boitsov, Daria S. Novikova

**Affiliations:** 1 Department of Organic Chemistry, St. Petersburg State Institute of Technology, 190013 St. Petersburg, Russia; 2 Department of Chemical Technology of Organic Dyes and Phototropic Compounds, St. Petersburg State Institute of Technology, 190013 St. Petersburg, Russia; 3 Saint-Petersburg Pasteur Institute, 197101 St. Petersburg, Russia; 4 Institute of Chemistry, St. Petersburg State University, 199034 St. Petersburg, Russia; 5 Laboratory of Nanobiotechnologies, St. Petersburg National Research Academic University of the Russian Academy of Science (Alferov University), 194021 St. Petersburg, Russia; 6 Laboratory of Cell Biotechnology, St. Petersburg State Institute of Technology, 190013 St. Petersburg, Russia

**Keywords:** acetylenes, phosphonates, anticancer activity, H1N1, molecular docking

## Abstract

This work focuses on rapid, catalyst-free synthesis of a new series of acetylenic phosphonates as promising building blocks for creating antiviral and anticancer agents. A comprehensive assessment of the biological activity of the synthesized compounds was conducted. Dialkyl phosphonates **4d**, **4e**, and **4g** were found to exhibit pronounced antiproliferative activity against human cancer cell lines, with the greatest IC_50_ = 6 μg/mL against the K562 cell line. Further studies revealed that these compounds cause significant disorganization of the actin cytoskeleton, leading to the loss of stress fibers and reduced cell motility. In contrast, diamide derivatives demonstrated a more favorable safety profile, with low cytotoxicity and moderate antiviral activity against influenza A (H1N1) virus, among which compound **6b** achieved a selectivity index of 5 with IC_50_ = 56.9 μg/mL. Screening studies of both dialkyl and diamide acetylenic phosphonates revealed some features of the interaction with kinase and nonkinase targets used for drug development and provide a basis for the subsequent rational design of novel selective anticancer agents based on the acetylenic phosphonate scaffold.

## 1. Introduction

Push-pull acetylenes are promising synthons in modern organic synthesis [1,2,3,4]. The presence of an activated triple bond in the compound makes it reactive toward various transformations, including cycloaddition reactions, which are currently in the spotlight [2,5,6,7], as well as somewhat less topical addition reactions [8,9,10,11]. The development of efficient and general approaches to the synthesis of push-pull acetylenes is an urgent task for synthetic chemists worldwide. Methods allowing one to produce phosphorus-containing derivatives of acetylene are of particular interest due to the unique role of these atoms in biological processes [12,13,14,15]. Currently, approaches to the synthesis of phosphorus-containing acetylenes are limited to metal-catalyzed reactions of terminal alkynes with dialkyl phosphites [16,17,18], which significantly narrows the potential range of accessible structures.

The presence of an acetylene moiety in the structure of a prodrug imparts several advantages in combating tumor cells. Thus, a number of studies have demonstrated that the rigid structure of the acetylene fragment provides selective interaction with tumor cell receptors. This is a necessary condition for proper orientation of the molecule in the active site of cancer cell enzymes and for enhancing the antiproliferative effect [19,20,21,22]. Using the example of a gold(I) complex ligand, which also contained an acetylene group, it was shown that the triple bond acts as a potential center for covalent or coordinate binding to biological targets [20]. The same effect was demonstrated upon the introduction of an acetylene moiety into nucleic acid bases, where the triple bond covalently linked to protein thiols [23].

Furthermore, the presence of a phosphonate moiety in prodrugs ensures better bioavailability of the final molecule [21,24]. Thus, the modification of betulin with an acetylene phosphonate fragment increases the lipophilicity of the molecule compared with the parent natural compound, which in turn contributes to improved bioavailability and anticancer activity [22]. The triple bond located adjacent to the phosphonate group creates steric hindrance for metabolic enzymes (e.g., cytochrome P450) attempting to attack and degrade the molecule [21]. Studies comparing the anticancer activity of acetylene compounds and their vinylphosphonate analogs have shown that the acetylene fragment is critically important for the interaction with the vitamin D receptor (VDR) through the histidine residues His305 and His397 [25]. A similar effect was demonstrated by the introduction of an acetylene group at the six position of the natural alkaloid β-carboline for the treatment of breast cancer [26]. Incorporation of the acetylene moiety enhanced cytotoxicity and provided selectivity for cancer cells due to high affinity for the active site of the EGFR receptor, binding via hydrogen bonds and hydrophobic interactions. All the above examples prove that the acetylene fragment is an important pharmacophore in the design of anticancer drugs, regardless of the original molecular scaffold.

In this work we present a series of new acetylenic phosphonates as promising building blocks for creating antiviral and anticancer agents. To prove this, we conducted primary experiments on cytotoxicity and antiviral activity, as well as more detailed studies of the anticancer activity. The prior ADMET prediction and virtual screening showed that the proposed molecules can be processed using rational design methodology and also outlined further promising directions.

## 2. Results

### 2.1. Chemistry

**Scheme 1:**
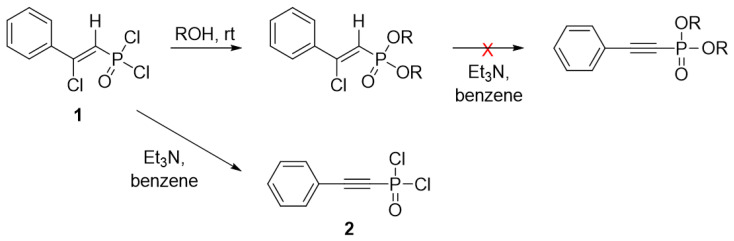
Synthesis of 2-phenylethynylphosphonic acid dichloroanhydride (**2**).

Previously, we achieved the selective synthesis of *Z*-2-phenyl-2-chloroethenylphosphonic acid dichloroanhydride **1** by reacting phosphorus pentachloride with phenylacetylene [27]. This compound exhibits high reactivity and readily reacts with alcohols (Scheme 1). It was shown that the reaction proceeds under mild conditions with quantitative yields. The anticholinesterase activity of the obtained phosphonates was evaluated. Additionally, the effect of laser radiation on the structure and properties of the resulting dialkyl *Z*-2-phenyl-2-chloroethenylphosphonates was investigated [28,29].

In further studies of the reactivity of *Z*-2-phenyl-2-chloroethenylphosphonic acid dichloroanhydride **1**, it was found that, under the action of bases, it eliminates hydrogen chloride and quantitatively affords 2-phenylethynylphosphonic acid dichloroanhydride **2** (Scheme 1). It should be noted that, when using *Z*-2-phenyl-2-chloroethenylphosphonic acid esters instead of the 2-phenylethynylphosphonic acid dichloroanhydride **2**, the dehydrohalogenation reaction could not be accomplished, which may be due to the lower activating effect of the ester group compared with the 2-phenylethynylphosphonic acid dichloroanhydride. These synthetic limitations do not allow one to synthesize esters of phenylacetylenephosphonic acid starting from the previously obtained esters of *Z*-2-phenyl-2-chloroethenylphosphonic acid.

**Scheme 2:**
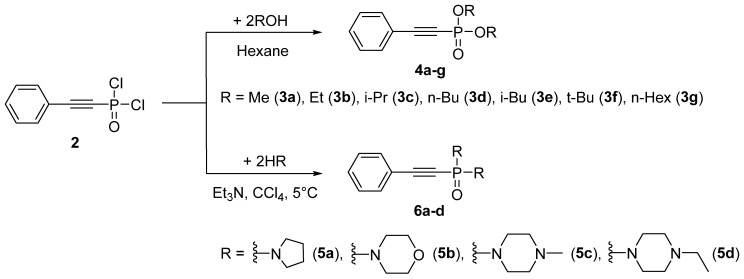
Synthesis of acetylene-containing phosphonates (**4a–c**, **6a–c**).

**Scheme 3:**
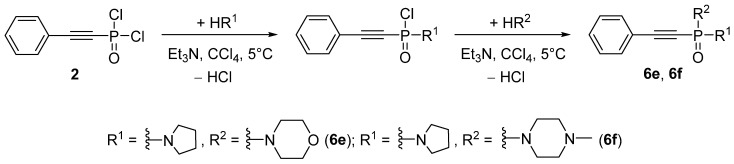
Synthesis of asymmetrical phosphonic acid diamides (**6e**, **6f**).

The present work focuses on the reactivity of 2-phenylethynylphosphonic acid dichloroanhydride **2** in reactions with nucleophiles such as aliphatic alcohols and secondary aliphatic amines. It was found that the reaction proceeds with the formation of symmetrical acetylene-containing phosphonates **4a–c**, **6a–c** (Scheme 2). Furthermore, carefully conducting the reaction protocol allows the asymmetrical phosphonic acide diamides **6e** and **6f** to be obtained in two steps (Scheme 3).

Compounds **4a**–**c** were obtained as yellow oils after workup, readily soluble in polar solvents. In contrast, compounds **6a**–**c**, **6e**, and **6f** were stable crystalline substances, also readily soluble in polar solvents. The yields of the final products ranged from 70% to 95%. The structures of the compounds were established on the basis of ^1^H, ^13^C, and ^31^P NMR spectroscopy, as well as X-ray diffraction analysis and high-resolution mass spectrometry.

**Figure 1:**
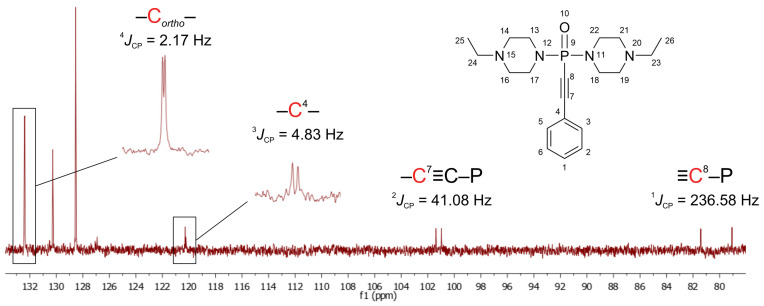
^13^C NMR spectrum of 1,1’-[(phenylethynyl)phosphoryl]di(ethylpiperazine) (**6d**).

The progress of the reaction and the purity of the obtained compounds were monitored by ^31^P NMR spectroscopy. The phosphorus chemical shift for the resulting 2-phenylethynylphosphonic acid dichloroanhydride **2** in deuterated chloroform is around −8 ppm, whereas for compounds **4a**–**c** the phosphorus signal shifts slightly downfield to −5 to −6 ppm. In contrast, the signal of amides **6a**–**c**, **6e**, and **6f** shifts downfield to +1.5 ÷ 5 ppm. As exemplified by compound **6d** (Figure 1), the presence of the triple bond in the final molecule of the entire series of the synthesized compounds is confirmed by the characteristic spin–spin coupling constants of the phosphorus nucleus with the triple-bond carbons. Thus, typical signals confirming the presence of the acetylenic bond in the ^13^C NMR spectra are doublets at 80.29 ppm and 101.13 ppm, with coupling constants of ^1^*J*_CP_ = 236.58 Hz and ^2^*J*_CP_ = 41.08 Hz, respectively.

**Figure 2:**
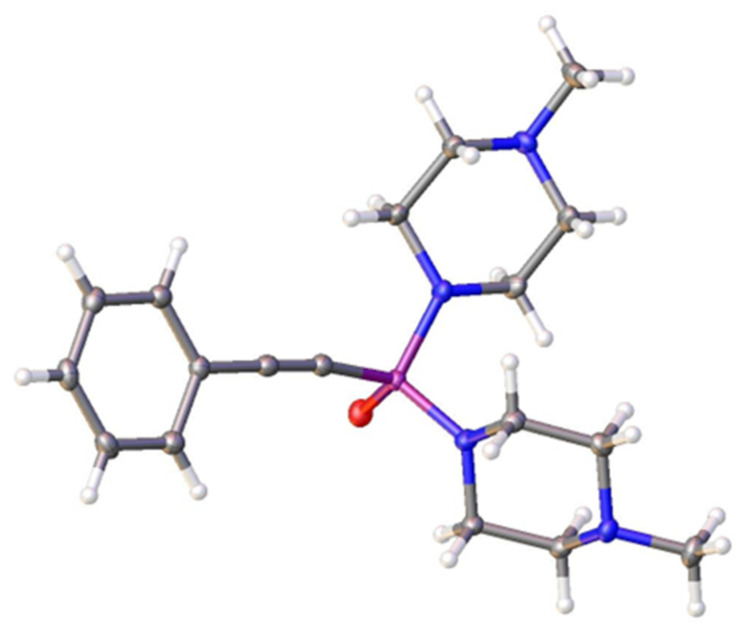
Structure of 1,1’-[(phenylethynyl)phosphoryl]di(methylpiperazine) (**6c**). CCDC 2543042.

The structure of compound (**6c**) was also confirmed by X-ray diffraction analysis (Figure 2). The crystal parameters are presented in Tables S1–S7 in the Supporting Information. The asymmetric unit contains one crystallographically independent molecule. The central phosphorus atom exhibits a distorted tetrahedral coordination, forming bonds with two nitrogen atoms of the fused heterocyclic system, one exocyclic oxygen atom (P=O), and one carbon atom of the bridging acetylene group.

The bond angles at P1 deviate significantly from ideal tetrahedral values: O1–P1–N1 = 118.92(8)°, N1–P1–N3 = 102.96(8)°, and O1–P1–C9 = 111.34(9)°, which is characteristic of a phosphoryl group (P=O). The P1–O1 bond length is 1.4792(14) Å, consistent with a double bond. The P1–C9 bond length is 1.767(2) Å, typical of a P–C(sp3) single bond. The C9–C10 bond is triple (1.204(3) Å) and serves as a bridge between the phosphoryl moiety and the cyclic system. The C10–C11 bond length (1.442(3) Å) corresponds to a conjugated enyne system. The torsion angles in the five-membered ring (N1–C1–C2–N2 and N1–C4–C3–N2) are 56.4(2)° and –60.8(2)°, respectively, indicating an ‘envelope’ conformation. The dihedral angles characterizing the orientation of the phosphoryl group relative to the piperazine ring (O1–P1–N1–C1 = 79.94(17)°, O1–P1–N1–C4 = –62.71(16)°) suggest a nearly perpendicular arrangement, which minimizes steric strain and facilitates hydrogen bonding.

### 2.2. Computational Studies

#### 2.2.1. ADMET Prediction

Evaluation of ADMET (absorption, distribution, metabolism, excretion, and toxicity) characteristics during the synthesis of new compounds is a mandatory step for drug development. Although in vitro tests are conducted at the preclinical stage [30], many virtual tools are available to researchers already at the early development stage [31], which allows one to significantly reduce the risk of failure and research costs. In our work we used SwissADME, a freely available web tool for evaluating pharmacokinetics, physicochemical properties, and drug-likeness of small molecules in silico [32]. For this, the structures of the synthesized compounds were converted to the canonical SMILES, which is required for generating the ADMET properties.

The bioavailability radar, used in SwissADME for rapid assessment of drug-likeness, relies on six physicochemical properties: lipophilicity, size, polarity, solubility, flexibility, and saturation. It looks like a diagram with the optimal ranges for each property mentioned (lipophilicity: XLOGP3 between −0.7 and +5.0; size: MW between 150 and 500 g/mol; polarity: TPSA between 20 and 130 Å^2^; solubility: log *S* ≤ 6; saturation: fraction of carbons in the sp^3^ hybridization ≥ 0.25; and flexibility: rotatable bonds ≤ 9). So the ADMET properties were predicted (Table 1), and the diagrams were obtained for each synthesized compound (Figures S68–S80).

According to the calculated data, most compounds show optimal values for all six properties, indicating good oral bioavailability. However, one derivative (**4g**) has high lipophilicity, determined by its structure, which includes two long alkyl chains resulting in an excessive number of rotatable bonds.

We also focused on two pharmacokinetic characteristics associated with the lipophilicity and polarity of the molecules. The first, gastrointestinal (GI) absorption, shows how effectively an oral drug will be absorbed into the body to calculate the dosage and, in case of low absorption, develop an alternative route of administration. All the synthesized compounds were predicted to have high GI absorption. The second, brain penetration, is important parameter for the drug to interact with therapeutic targets inside the brain. As expected, it was predicted that compound **4g** would not penetrate BBB. However, compound **6c** was indicated as not permeating BBB, while other structures are promising candidates for interacting with brain proteins.

The presence of phosphorus and a triple bond are distinctive features of the proposed compounds; however, the Brenk filters indicated these as two alerts. No alerts were observed in the PAINS scores for any of the structures. In general, the drug-likeness of the considered acetylene-containing phosphonates was confirmed, although for compound **4g** this was only true only when using the Lipinsky method.

#### 2.2.2. Virtual Screening

One of the ways to predict the biological target of a synthesized compounds is through virtual screening (VS), which avoids testing millions of real molecules in the laboratory. Molecular docking methods are widely used as part of a structure-based approach to in silico target identification [33]. This method involves ‘fitting’ a molecule to three-dimensional models of target proteins and calculating parameters that allow one to assess the strength of the binding.

Based on a literature review, our scientific background, and accumulated experience, as well as computer-based biological activity prediction using the Pass online web resource [34], we hypothesized that all compounds can exhibit some antineoplastic activity. According to these predictions, compounds **6e** and **6f** were of the greatest interest. They yield maximum probabilities (0.868–0.892) with high statistical significance (*p* = 0.001–0.005), indicating a practical candidate for the treatment of non-Hodgkin’s lymphoma. Also, according to initial screening using PASS, compounds in series **4** can simultaneously inhibit the proteasome, GST, hydrolases, and proteases, making it more difficult to develop resistance to a drug that acts on several independent pathways in the context of tumor therapy.

Therefore, we placed the greatest emphasis on anticancer activity during screening and conducted virtual screening of the synthesized compounds for the most popular anticancer targets used in rational drug design, as well as some specific targets from predictions. These targets are key signaling proteins regulating cell function, survival, and proliferation, represented by both receptor and cytoplasmic protein kinases, as well as other important enzymes, including the repair system and transport proteins [35,36,37]. However, for a complete picture, we also included several more antiviral targets, as phosphonates are known to confer antiviral activity [38].

As can be seen from Table 2, the diester derivatives show moderate to significant affinity for kinase and nonkinase targets, except for **4d**. Interestingly, the minimum values of the analyzed scoring function (Docking Score) were obtained for PDGFRA, which is known to interact with acetylenic inhibitors [39].

**Figure 3:**
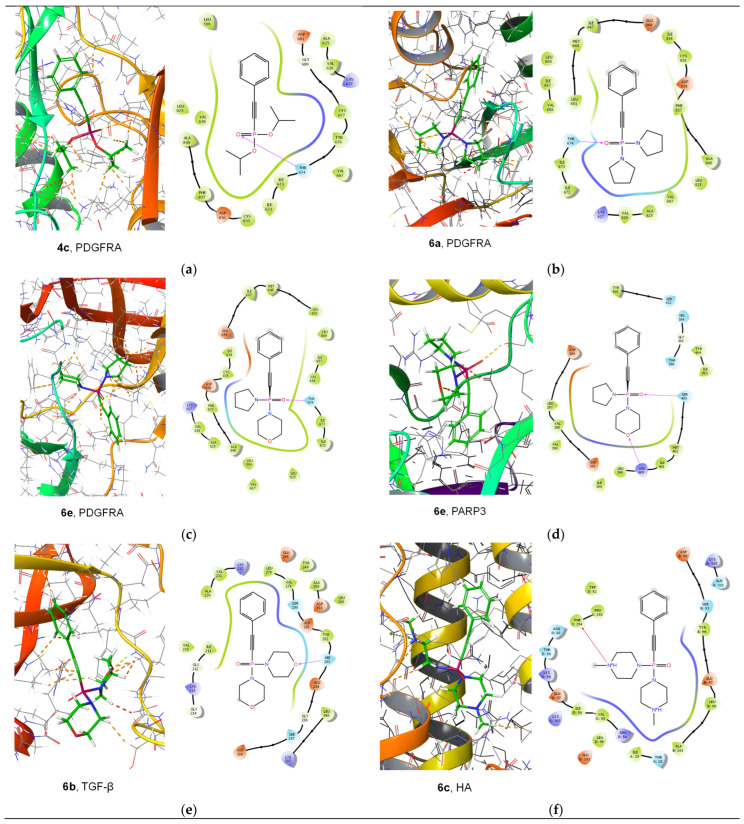
Virtual screening results for **4c**, **6a**, **6b**, **6c**, and **6e** with the target proteins (PDGFRA, PDB id: 6JOL; PARP3, PDB id: 4GV0; TGF-β, PDB id: 1PY5; HA, PDB id: 3EYM).

Virtual screening of diamine derivatives resulted in generally lower Docking Score values for the same protein targets (Table 3). However, another pattern was observed for this group of compounds towards nonkinase targets and antiviral proteins, demonstrating higher calculated activity compared with diester derivatives. The most interesting results for both studied groups of compounds are visualized in Figure 3.

Altogether, the virtual screening data indicate that the method of molecular docking can effectively be used for proposing the target and further optimizing the studied compounds, which are usually expected to react like classical organophosphorus compounds through covalent interactions [40,41,42].

### 2.3. Biological Testing

#### 2.3.1. Antiviral Activity

Studying the antiviral activity of phosphonates is a fairly common practice, since it often leads to good results [43,44]. There are also examples when the replacement of the carboxyl function with the phosphonate one led to an increase in antiviral activity [45]. Antiviral activity is often studied in conjunction with anticancer activity, including evaluation of the selectivity index. We have previously conducted similar combinations of studies of the biological activity of other structures, and interesting results were obtained [46].

During primary screening, the antiviral and cytotoxic activity of the synthesized phosphinoxane derivatives containing diamine fragments **4a**–**g** and alkynyl substituents **6a**–**f** was evaluated. Studies were conducted in the MDCK cell line (Madin–Darby canine kidney cells) and for influenza A virus (H1N1) strain A/Puerto Rico/8/34. Among the **4a**–**g** compounds tested, most exhibited high cytotoxicity (Table 4). The CC_50_ values for compounds **4a**, **4c**, **4d**, **4e**, and **4g** ranged from 4.1 to 59.9 μg/mL. However, their IC_50_ values exceeded the maximum tested concentrations (>3–33 μg/mL), which prevented SI calculation or resulted in values less than 1. Compound **4f** stood out, with the lowest cytotoxicity (CC_50_ > 300 μg/mL), but its antiviral activity also failed to reach measurable levels (IC_50_ > 100 μg/mL).

A different pattern was observed for compounds **6a–f**. Compound **6b** is of the greatest interest, with recorded values of CC_50_ > 300 μg/mL and IC_50_ = 56.9 μg/mL, corresponding to SI = 5, which is the maximum value among all the tested samples. Compounds **6e** and **6f** also demonstrated low cytotoxicity (CC_50_ > 300 μg/mL) and moderate antiviral activity (IC_50_ = 140 and 71.7 μg/mL, respectively), with SI values of 2 and 4. For compound **6c**, both parameters exceeded 300 μg/mL, indicating low activity within the tested concentration range. Compounds **6a** and **6d** showed high cytotoxicity (CC_50_ = 56.2 and 39.8 μg/mL, respectively) and low antiviral activity, resulting in SI = 1. Thus, compound **6b** possessed the most promising activity profile according to the primary assessment of the antiviral activity.

#### 2.3.2. Anticancer Activity

The anticancer activity is firstly attributed to the presence of the acetylene fragment. The triple bond in the structure of the compounds can reasonably be considered a pharmacophore imparting anticancer activity [47,48,49,50]. So, the antiproliferative potential of the synthesized compounds **4a–g** and **6a–f** in human erythroleukemia (K562), cervical carcinoma (HeLa), and melanoma (Sk-mel-2) cell lines was evaluated by the standard MTS assay for 24 and 72 h in vitro. The results of this study are summarized in Table 5 as IC_50_ values of the tested diesters and diamides.

**Figure 4:**
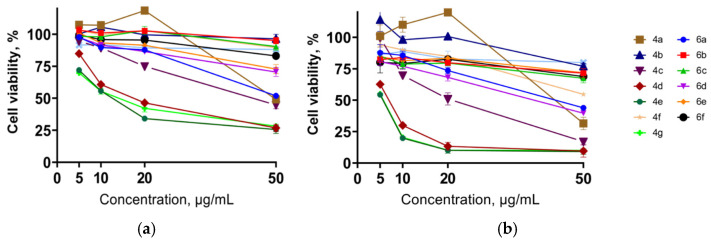
Cytotoxicity of compounds **4a**–**g** and **6a**–**f** in the K562 cell line after treatment for 24 (**a**) and 72 h (**b**).

**Figure 5:**
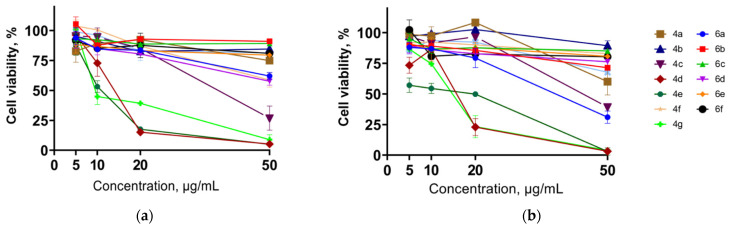
Cytotoxicity of compounds **4a**–**g** and **6a**–**f** in the HeLa cell line after treatment for 24 (**a**) and 72 h (**b**).

**Figure 6:**
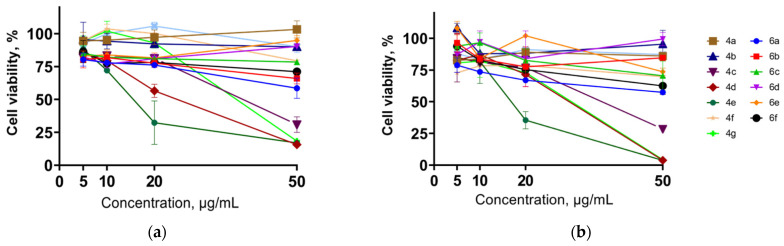
Cytotoxicity of compounds **4a**–**g** and **6a**–**f** in the Sk-mel-2 cell line after treatment for 24 (**a**) and 72 h (**b**).

It should be noted that the revealed antiproliferative effects of the synthesized compounds are comparable with those of cisplatin and doxorubicin [51,52]. As can be seen from the obtained data, diesters **4a–g** generally showed greater cytotoxic activity as compared with diamides **6a–g** in all the tested cell lines. It was found that, among the first group, butyl, isobutyl, and hexyl esters (**4d**, **4e**, and **4g**) were the most active, demonstrating antiproliferative activity with IC_50_ values of about 6 μg/mL in the K562 cell line (Figure 4) and 13–25 μg/mL in both the HeLa (Figure 5) and Sk-mel-2 cell lines (Figure 6) after treatment for 72 h. At the same time, the IC_50_ values for diamides **6a–f** did not exceed 40 μg/mL. The best results were shown for di(tetramethylenamide) **6a** (44 μg/mL) and di(3-aza-3-ethyl-pentamethylenamide) **6d** (40 μg/mL) in the K562 cell line after 72 h of treatment.

Additional experiments were conducted to determine the selectivity index of the most promising compounds of series **4**, which resulted in identification of the candidate **4g**, with the selectivity index up to 19 (Table 6).

#### 2.3.3. Actin Cytoskeleton Changes

Actin plays a vital role in key cellular processes like migration, adhesion, and morphogenesis. The actin cytoskeleton supports various cell activities through its dynamic restructuring, and disruptions in this process can impair cell motility. Actin organization patterns serve as indicators for assessing the metastatic potential of cancer cells [53,54].

**Figure 7:**
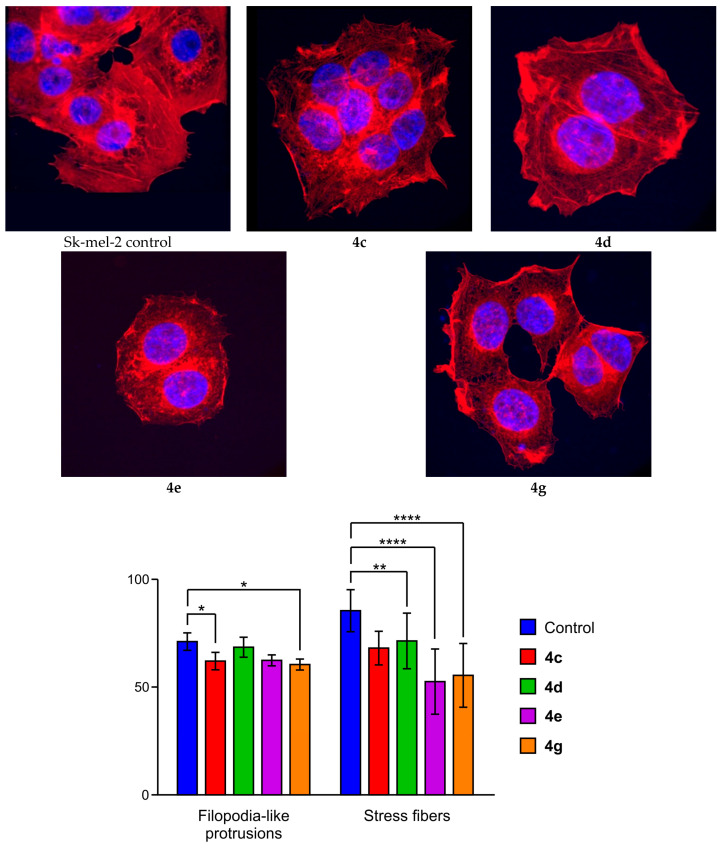
Microscopic images of treated cells and state of the actin cytoskeleton in Sk-mel-2 cells after treatment with compounds **4c**, **4d**, **4e**, and **4g** (10 μg/mL). *p*-value < 0.05 (*), 0.01 (**), 0.001 (****).

**Figure 8:**
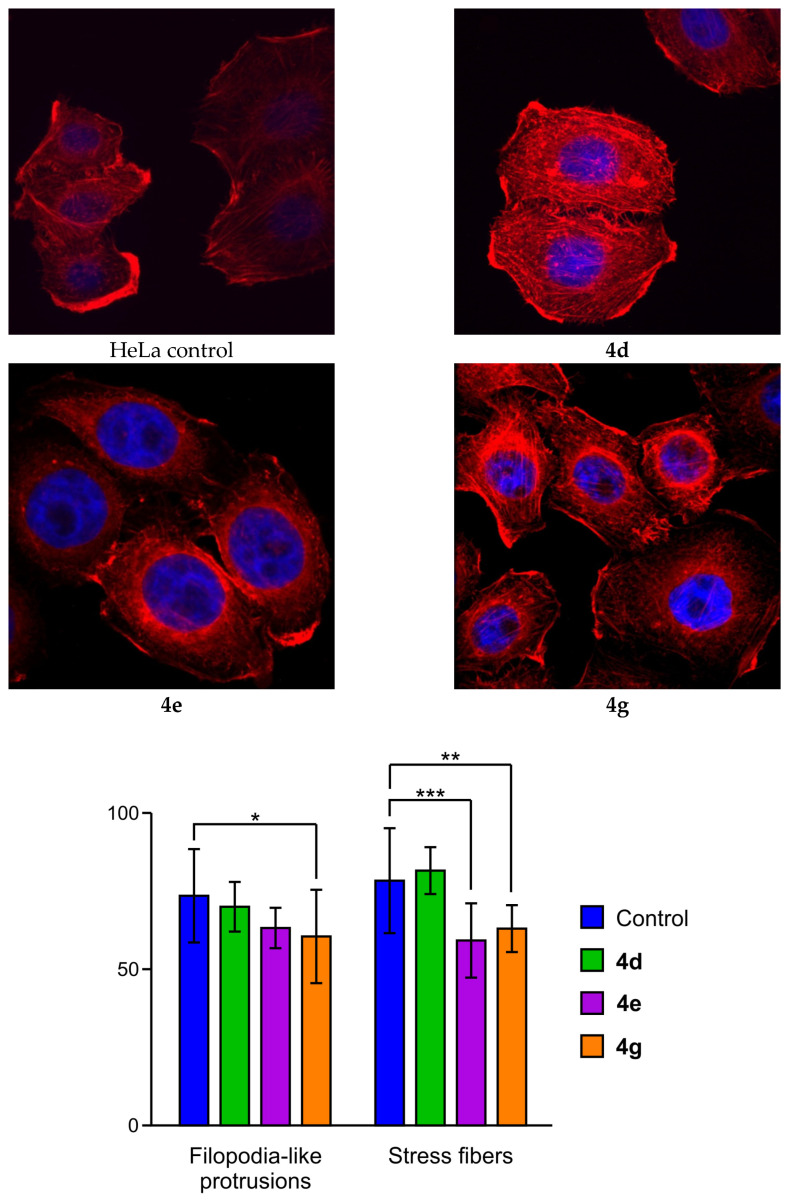
Microscopic images of treated cells and state of the actin cytoskeleton of Sk-mel-2 cells after treatment with compounds **4d**, **4e**, and **4g** (10 μg/mL). *p*-value < 0.05 (*), 0.01 (**), 0.001 (***).

The most promising compounds were evaluated for their ability to modify the actin cytoskeleton structure in HeLa and Sk-mel-2 cells, focusing on the presence of stress fibers and filopodia-like protrusions. Confocal microscopy revealed that incubation with the studied compounds **4c**, **4d**, **4e**, and **4g** induced major alterations in the actin cytoskeleton of both Sk-mel-2 (Figure 7) and HeLa cells (Figure 8). This resulted in the loss of stress fibers, with granular actin dispersing diffusely in the cytoplasm of up to 47% of treated cells for Sk-mel-2 and 52% of treated cells for HeLa. Also, changes in filopodia-like protrusions, represented by a post-incubation increase of up to 55% for Sk-mel-2 cells and 52% for HeLa cells, were noted.

The revealed cytoskeletal alterations may signal shifts in cellular motility. No nuclear fragmentation occurred during the experiment, suggesting a lack of proapoptotic effects. The data on actin cytoskeleton organization, along with percentages of cells showing filopodia-like protrusions and disrupted stress fibers, are presented together in Figure 7 and Figure 8.

#### 2.3.4. Wound-Healing Ability

**Figure 9:**
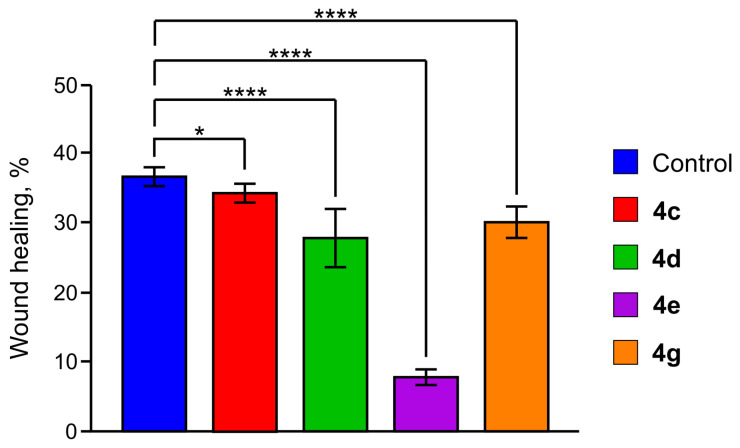
Wound-healing ability of Sk-mel-2 cells after treatment with compounds **4c**, **4d**, **4e**, and **4g**. *p*-value < 0.05 (*), 0.001 (****).

**Figure 10:**
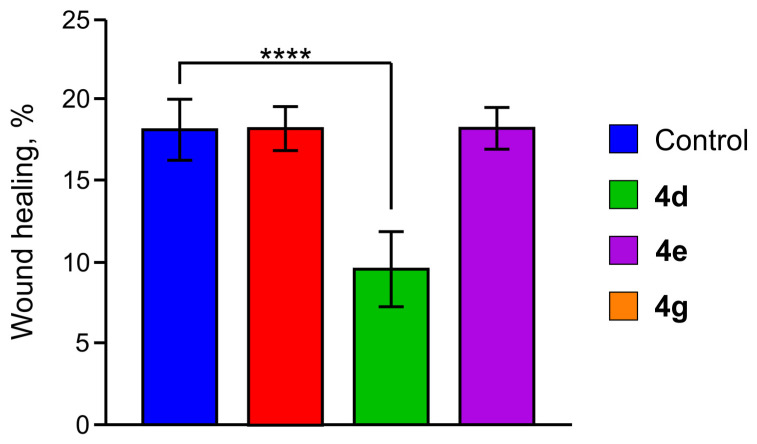
Wound-healing ability of HeLa cells after treatment with compounds **4d**, **4e**, and **4g**. *p*-value < 0.001 (****).

Additionally, a scratch test was performed to study the wound-healing ability of Sk-mel-2 cells treated with compounds **4c**, **4d**, **4e**, and **4g** and HeLa cells treated with **4d**, **4e**, and **4g**. The results are presented in Figure 9 and Figure 10 (see also the Supporting Information, Figures S66 and S67). The measured wound areas in Sk-mel-2 cells after 24 h of the treatment with compounds **4c**, **4d**, **4e**, and **4g** were 35, 30, 7, 20, and 37% for the tested compounds and control sample, respectively. For HeLa cells, the measured wound areas were 27, 7, 28, and 27% for compounds **4d**, **4e**, and **4g** and the control sample, respectively. So, compound **4e**, which led to less stress fiber formation and filopodia-like deformations (as compared with the control), resulted in low wound-healing ability in both cell lines. As can be seen from the obtained data, the wound-healing ability data are consistent with the cytoskeleton data.

## 3. Materials and Methods

### 3.1. Chemicals and Analytical Methods

All chemicals and solvents used in the experiments were purchased from commercial sources; unless otherwise stated, all chemicals and solvents were used without further purification. With TMS as an internal standard, ^1^H NMR and ^13^C NMR spectra were collected in CDCl_3_ on a Bruker Avance III HD 400 NanoBay (400.17 MHz ^1^H, 100.62 MHz ^13^C, 161.98 MHz ^31^P) spectrometer (Bruker Corporation, Billerica, MA, USA) at room temperature, the chemical shifts (δ) were expressed in parts per million (ppm), and J values were given in hertz (Hz). Phosphorus chemical shifts are reported relative to an external standard of 85% phosphoric acid. Melting points were measured on a Kofler hot stage (VEB Wägetechnik Rapido, PHMK 81/2969).

HPLC analysis was performed on an LC-20 Prominence (Shimadzu, Kyoto, Japan) using a Nucleodur PolarTec column (Macherey-Nagel, Dueren, Germany), length 150 mm, internal diameter 3.0 mm, particle size 3 μm, in acetonitrile–0.1% trifluoroacetic acid (70/30, 50/50, 40/60) or acetonitrile–water (70/30), flow rate 0.4 mL/min, oven temperature 40 °C.

Mass spectra were acquired using a TIMS TOF Pro (ESI-TOF) time-of-flight mass spectrometer (Bruker Daltonics, Germany). The mass spectrometric parameters were as follows: MS full-scan mode, positive ion detection in an m/z range of 20–1300, and a scan rate of 0.5 Hz. The ion source conditions were as follows: capillary voltage 4500 V, drying gas flow 8 L/min, drying gas temperature 250 °C, and nebulizer gas pressure 2.2 bar. The transfer parameters were as follows: Funnel 1 RF 200 Vpp, Funnel 2 RF 200 Vpp, isCID energy 0 eV, Multipole RF 200 Vpp, and Deflection Delta 60 V. The quadrupole settings were as follows: ion energy 5 eV and isolation mass set to 60 m/z. The collision cell was operated with a collision energy of 7 eV, an RF of 700 Vpp, and a transfer time of 80 μs. Calibration of the mass spectrometer was performed using a 1 mM sodium formate solution in water.

X-ray diffraction study of compound **6c** was performed at 100(2) K on a Rigaku XtaLAB SuperNova diffractometer (HyPix-3000 type detector) using Cu Kα (λ = 1.54184 Å) radiation. The structure was solved with the ShelXT [55] structure solution program using Intrinsic Phasing and refined with the ShelXL [56] refinement package incorporated into the OLEX2 program package [57], using Least Squares minimization. Empirical absorption correction was applied in the CrysAlisPro [58] program complex using spherical harmonics, implemented in the SCALE3 ABSPACK scaling algorithm. The hydrogen atom positions were fixed geometrically at calculated distances and allowed to ride on the parent atoms.

CCDC 2543042 contains the supplementary crystallographic data for this paper. These data can be obtained free of charge from The Cambridge Crystallographic Data Centre via http://www.ccdc.cam.ac.uk (accessed on 1 April 2026).

### 3.2. Synthetic Procedures 

The dichloroanhydride of 2-phenylethenylphosphonic acid **1** was synthesized according to a previously described procedure [27].

**Dichloroanhydride of 2-phenylethynylphosphonic acid (2)**. A three-necked flask equipped with a mechanical stirrer was charged with 250 mL of anhydrous benzene, 15 g (0.059 mol) of the dichloroanhydride of 2-phenyl-2-chloroethenylphosphonic acid (**1**), and 8.94 g (0.089 mol) of triethylamine. The reaction proceeded at the boiling point of benzene. Upon completion of the reaction, the triethylamine salt formed was filtered off using a sintered glass filter. The benzene was then distilled off, and the residue was distilled under deep vacuum (1 mmHg). The product obtained was a yellow oil. ^1^H NMR (400 MHz, CDCl_3_): δ 7.37–7.44 (m, 3H), 7.56–7.63 (m, 2H). ^13^C NMR (100 MHz, CDCl_3_): δ 89.67 (d, ^1^*J*_CP_ = 236.9 Hz), 99.10 (d, ^2^*J*_CP_ = 38.8 Hz), 121.56–121.67 (m), 129.00, 131.00, 132.00. ^31^P NMR (161 MHz, CDCl_3_): δ −8.48.

**General procedure for the synthesis of symmetrical dialkyl(phenylethynyl)phosphonate (4a–c)**. In a round-bottom flask equipped with a magnetic stirrer, the dichloroanhydride of 2-phenylethynylphosphonic acid (**1**) (0.005 mol) was dissolved in 25 mL of hexane. A mixture of the alcohol (0.01 mol) and potash (0.0125 mol) in 5 mL of tetrachloromethane was added dropwise slowly with constant stirring and cooling at 5 °C. The reaction mixture was subsequently stirred at room temperature for 2–5 h under an argon atmosphere. Then, the reaction mass was filtered, the potash was washed with tetrahydrofuran tetrahydrofuran (3 × 10 mL), and the extract was concentrated. The final product obtained was a yellow oil.

*Dimethyl(phenylethynyl)phosphonate* (**4a**). Yellow viscous oil; yield 95%. ^1^H NMR (400 MHz, CDCl_3_) δ 3.84 (d, ^2^*J*_HP_ = 12.2 Hz, 6H), 7.40 (t, ^3^*J*_HH_ 7.4 Hz, 2H), 7.45 (t, ^3^*J*_HH_ = 7.5 Hz, 2H), 7.56 (d, ^3^*J*_HH_ = 6.9 Hz, 1H). ^13^C NMR (101 MHz, CDCl_3_) δ 53.46 (d, ^2^*J*_CP_ = 5.5 Hz), 76.86 (d, ^1^*J*_CP_ = 302.2 Hz), 99.95 (d, ^2^*J*_CP_ = 53.2 Hz), 119.24 (d, ^4^*J*_CP_ = 5.5 Hz), 128.62, 130.90, 132.69 (d, ^2^*J*_CP_ = 2.6 Hz). ^31^P NMR (161 MHz, CDCl_3_): δ—2.33. HRMS (ESI-TOF) for C_10_H_11_O_3_P [M+H]^+^ m/z calcd 211.1688, obsd. 211.0525.

*Diethyl(phenylethynyl)phosphonate* (**4b**). Yellow viscous oil; yield 94%. ^1^H NMR (400 MHz, CDCl_3_) δ 1.40 (t, *J* = 7.1 Hz, 6H), 4.28–4.17 (m, 4H), 7.37 (t, ^3^*J*_HH_ = 7.8 Hz, 2H), 7.45 (t, ^3^*J*_HH_ = 6.7 Hz, 2H), 7.56 (d, ^3^*J*_HH_ = 8.4 Hz, 1H). ^13^C NMR (101 MHz, CDCl_3_) δ 23.79 (d, ^3^*J*_CP_ = 4.40 Hz), 23.52 (d, ^3^*J*_CP_ = 5.14 Hz), 72.16 (d, ^2^*J*_CP_ = 5.50 Hz), 79.77 (d, ^1^*J*_CP_ = 297.84 Hz), 98.00 (d, ^2^*J*_CP_ = 52.45 Hz), 119.59 (d, ^4^*J*_CP_ = 5.50 Hz), 128.50, 130.50, 132.31 (d, ^2^*J*_CP_ = 2.57 Hz). ^31^P NMR (161 MHz, CDCl_3_): δ—5.85. HRMS (ESI-TOF) for C_12_H_15_O_3_P [M+Na]^+^ m/z calcd 261.0656, obsd. 261.0642.

*Diisopropyl(phenylethynyl)phosphonate* (**4c**). Yellow viscous oil; yield 94%. ^1^H NMR (400 MHz, CDCl_3_) δ 1.26 (dd, ^3^*J*_HP_ = 2.57 Hz, ^3^*J*_HH_ = 6.4 Hz, 12H), 4.66 (dh, ^2^*J*_HP_ = 8.7 Hz, ^3^*J*_HH_ = 6.1 Hz, 2H), 7.21 (t, ^3^*J*_HH_ = 7.3 Hz, 2H), 7.29 (d, ^3^*J*_HH_ = 7.5 Hz, 2H), 7.39 (t, ^3^*J*_HH_ = 7.1 Hz, 1H). ^13^C NMR (101 MHz, CDCl_3_) δ 23.79 (d, ^3^*J*_CP_ = 4.4 Hz), 23.52 (d, ^3^*J*_CP_ = 5.1 Hz), 72.16 (d, ^2^*J*_CP_ = 5.5 Hz), 79.77 (d, ^1^*J*_CP_ = 297.8 Hz), 98.00 (d, ^2^*J*_CP_ = 52.5 Hz), 119.59 (d, ^4^*J*_CP_ = 5.5 Hz), 128.50, 130.50, 132.31 (d, ^2^*J*_CP_ = 2.6 Hz). ^31^P NMR (161 MHz, CDCl_3_): δ—8.81. HRMS (ESI-TOF) for C_14_H_19_O_3_P [M+Na]^+^ m/z calcd 289.0970, obsd. 289.0951.

*Dibutyl(phenylethynyl)phosphonate* (**4d**). Yellow viscous oil; yield 92%. ^1^H NMR (400 MHz, CDCl_3_) δ 0.97 (t, ^3^*J*_HH_ = 7.4 Hz, 6H), 1.48 (h ^3^*J*_HH_ = 7.3 Hz, 4H), 1.79–1.70 (m, 4H), 4.20–4.14 (m, 4H), 7.39 (t, ^3^*J*_HH_ = 7.3 Hz, 2H), 7.47 (t, ^3^*J*_HH_ = 7.5 Hz, 2H), 7.58 (d, ^3^*J*_HH_ = 6.8 Hz, 1H). ^13^C NMR (101 MHz, CDCl_3_) δ 13.58, 18.72, 32.22 (d, ^3^*J*_CP_ = 7.3 Hz), 66.92 (d, ^2^*J*_CP_ = 5.9 Hz), 78.39 (d, ^1^*J*_CP_ = 299.7 Hz), 99.03 (d, ^2^*J*_CP_ = 52.8 Hz), 119.65 (d, ^4^*J*_CP_ = 5.5 Hz), 128.57, 130.66, 132.62 (d, ^2^*J*_CP_ = 2.6 Hz). ^31^P NMR (161 MHz, CDCl_3_): δ—5.59. HRMS (ESI-TOF) for C_16_H_23_O_3_P [M+H]^+^ m/z calcd 295.3359, obsd. 295.1459.

*Diisobutyl(phenylethynyl)phosphonate* (**4e**). Yellow viscous oil; yield 93%^1^H NMR (400 MHz, CDCl_3_) δ 1.00 (d, ^3^*J*_HH_ = 6.7 Hz, 12H), 2.04 (dn, ^2^*J*_HP_ = 13.3, ^3^*J*_HH_ = 6.7 Hz, 2H), 3.93 (td, ^3^*J*_HH_ = 7.1, ^2^*J*_HP_ = 1.3 Hz, 4H), 7.39 (t, ^3^*J*_HH_ = 7.3 Hz, 2H), 7.47 (t, ^3^*J*_HH_ = 7.5 Hz, 2H), 7.57 (d, ^3^*J*_HH_ = 6.8 Hz, 1H). ^13^C NMR (101 MHz, CDCl_3_) δ 18.72 (d, ^4^*J*_CP_ = 2.2 Hz), 29.05 (d, ^3^*J*_CP_ = 7.3 Hz), 73.06 (d, ^2^*J*_CP_ = 6.2 Hz), 78.32 (d, ^1^*J*_CP_ = 300.4 Hz), 99.12 (d, ^2^*J*_CP_ = 52.8 Hz), 119.65 (d, ^4^*J*_CP_ = 5.9 Hz), 128.58, 130.66, 132.62 (d, ^2^*J*_CP_ = 2.6 Hz). ^31^P NMR (161MHz, CDCl_3_): δ—5.50. HRMS (ESI-TOF) for C_16_H_23_O_3_P [M+Na]^+^ m/z calcd 295.3359, obsd. 295.1459.

*Ditert-butyl(phenylethynyl)phosphonate* (**4f**). Yellow viscous oil; yield 88%^1^H NMR (400 MHz, CDCl_3_) δ 1.85 (m, 18H), 7.04-7.36 (m, 4H), 7.40–7.57 (m, 1H). ^13^C NMR (101 MHz, CDCl_3_) δ 25.53, 68.06 (d, ^2^*J*_CP_ = 5.8 Hz), 78.32 (d, ^1^*J*_CP_ = 300.4 Hz), 97.33 (d, ^2^*J*_CP_ = 52.56 Hz), 119.97 (d, ^4^*J*_CP_ = 5.4 Hz), 128.28, 130.13, 132.57 (d, ^2^*J*_CP_ = 2.6 Hz). ^31^P NMR (161 MHz, CDCl_3_): δ—5.30. HRMS (ESI-TOF) for C_16_H_23_O_3_P [M+Na]^+^ m/z calcd 317.1283, obsd. 317.1326. HRMS (ESI-TOF) for C_16_H_23_O_3_P [M+H]^+^ m/z calcd 295.3343, obsd. 295.1458.

*Dihexyl(phenylethynyl)phosphonate* (**4g**). Yellow viscous oil; yield 94%^1^H NMR (400 MHz, CDCl_3_) δ 0.93–0.85 (m, 3H). 1.37–1.27 (m, 4H), 1.43 (p, ^3^*J*_HH_ = 7.1 Hz, 4H), 1.79–1.70 (m, 4H), 4.19–4.12 (m, 4H), 7.38 (t, ^3^*J*_HH_ = 7.4 Hz, 2H), 7.46 (t, ^3^*J*_HH_ = 7.5 Hz, 2H), 7.57 (d, ^3^*J*_HH_ = 7.0 Hz, 1H). ^13^C NMR (101 MHz, CDCl_3_) δ 13.97, 22.53, 25.18, 30.19, 31.31 (d, ^3^*J*_CP_ = 7.0 Hz), 67.26 (d, ^2^*J*_CP_ = 5.9 Hz), 78.40 (d, ^1^*J*_CP_ = 299.3 Hz), 99.04 (d, ^2^*J*_CP_ = 52.8 Hz), 119.64 (d, ^4^*J*_CP_ = 5.9 Hz), 128.56, 130.66, 132.62 (d, ^2^*J*_CP_ = 2.6 Hz). ^31^P NMR (161 MHz, CDCl3): δ—6.13. HRMS (ESI-TOF) for C_20_H_31_O_3_P [M+H]^+^ m/z calcd 351.3301, obsd. 351.2085.

**General procedure for the synthesis of symmetrical phosphonic acid diamides (6a–d)**. In a round-bottom flask equipped with a magnetic stirrer, the dichloroanhydride of 2-phenylethynylphosphonic acid (**1**) (0.005 mol) was dissolved in 25 mL of tetrachloromethane. A mixture of the amine (0.01 mol) and triethylamine (0.0125 mol) in 5 mL of tetrachloromethane was added dropwise slowly with constant stirring and cooling at 5 °C. The reaction mixture was subsequently stirred at room temperature for 13–26 h under an argon atmosphere. Then, the solvent was distilled off, the reaction mixture was extracted with tetrahydrofuran (3 × 10 mL), and the extract was concentrated. The residue was purified by column chromatography (alumina-silica gel, CH_2_Cl_2_/MeOH 95:5). The resulting yellow oils were subsequently recrystallized from a hexane/benzene (99:1) solution. The final product obtained was white crystals.

*1,1’-[(phenylethynyl)phosphoryl]dipyrrolidine* (**6a**). Colorless solid; yield 88%; mp 223–227 °C. ^1^H NMR (400 MHz, CDCl_3_): δ 1.4 –1.44 (m, 8H), 2.69–2.84 (m, 8H), 6.90–6.98 (m, 3H), 7.07–7.09 (m, 2H). ^13^C NMR (100 MHz, CDCl_3_): δ 25.94 (d, ^3^*J*_CP_ = 8.4 Hz), 45.61 (d, ^2^*J*_CP_ = 4.8 Hz), 81.31 (d, ^1^*J*_CP_ = 226.5 Hz), 98.50 (d, ^2^*J*_CP_ = 39.5 Hz), 120.28 (d, ^3^*J*_CP_ = 4.8 Hz), 128.15, 129.61, 131.89 (d, ^4^*J*_CP_ = 2.1 Hz). ^31^P NMR (161 MHz, CDCl_3_): δ 1.17. HRMS (ESI-TOF) for C_16_H_21_N_2_OP [M+H]^+^ m/z calcd 289.1391, obsd. 289.1454.

*4,4’-[(phenylethynyl)phosphoryl]dimorpholine* (**6b**). Colorless solid; yield 90%; mp 66.5–67 °C. ^1^H NMR (400 MHz, CDCl_3_): δ 3.27 (t, ^3^*J*_HH_ = 8.50 Hz, 8H), 3.68 (t, ^2^*J*_HP_ = 4.69 Hz, 8H), 7.26–7.45 (m, 3H), 7.54 (d, ^3^*J*_HH_ = 7.54 Hz, 2H). ^13^C NMR (100 MHz, CDCl_3_): δ 44.16, 66.97 (d, ^2^*J*_CP_ = 6.2 Hz), 79.73 (d, ^1^*J*_CP_ = 237.4 Hz), 101.64 (d, ^2^*J*_CP_ = 40.8 Hz), 119.93 (d, ^3^*J*_CP_ = 4.8 Hz), 128.60, 130.48, 132.4 (d, ^4^*J*_CP_ = 2.2 Hz). ^31^P NMR (161 MHz, CDCl_3_): δ 4.70. HRMS (ESI-TOF) for C_16_H_21_N_2_O_2_P [M+H]^+^ m/z calcd 321.1290, obsd. 321.1354.

*1,1’-[(phenylethynyl)phosphoryl]di(methylpiperazine)* (**6c**). Colorless solid; yield 85%; mp 122–122.4 °C. ^1^H NMR (400 MHz, CDCl_3_): δ 2.30 (s, 6H), 2.43 (t, 8H), 3.21–3.28 (m, 8H), 7.28–7.42 (m, 3H), 7.49–7.51 (m, 2H,). ^13^C NMR (100 MHz, CDCl_3_): δ 15.25, 43.79 (d, ^3^*J*_CP_ = 2.9 Hz), 55.05 (d, ^2^*J*_CP_ = 6.3 Hz), 80.29 (d, ^1^*J*_CP_ = 236.9 Hz), 101.13 (d, ^2^*J*_CP_ = 40.9 Hz), 120.24 (d, ^3^*J*_CP_ = 4.9 Hz), 128.53, 130.26, 132.37 (d, ^4^*J*_CP_ = 2.2 Hz). ^31^P NMR (161 MHz, CDCl_3_): δ 5.09. HRMS (ESI-TOF) for C_18_H_27_N_4_OP [M+H]^+^ m/z calcd 347.1922, obsd. 347.1985.

*1,1’-[(phenylethynyl)phosphoryl]di(ethylpiperazine)* (**6d**). Colorless solid; yield 84%; mp 223–227 °C. ^1^H NMR (400 MHz, CDCl_3_): δ 1.11 (t, 6H), 2.43–2.51 (m, 12H), 3.18–3.31 (m, 8H), 7.34–7.45 (m, 3H), 7.50–7.53 (m, 2H). ^13^C NMR (100 MHz, CDCl_3_): δ 11.69, 43.86 (d, ^3^*J*_CP_ = 2.6 Hz), 52.73 (d, ^2^*J*_CP_ = 6.3 Hz), 80.25 (d, ^1^*J*_CP_ = 236.9 Hz), 101.18 (d, ^2^*J*_CP_ = 40.9 Hz), 120.26 (d, ^3^*J*_CP_ = 4.8 Hz), 128.54, 130.27, 132.39 (d, ^4^*J*_CP_ = 2.2 Hz). ^31^P NMR (161 MHz, CDCl_3_): δ 4.96. HRMS (ESI-TOF) for C_20_H_31_N_4_OP [M+H]^+^ m/z calcd 375.2235, obsd. 375.2297.

**General procedure for the synthesis of asymmetrical phosphonic acid diamides (6e, 6f)**. In a round-bottom flask equipped with a magnetic stirrer, the dichloroanhydride of 2-phenylethynylphosphonic acid (**1**) (0.005 mol) was dissolved in 25 mL of tetrachloromethane. A mixture of the first amine (R^1^) (0.0025 mol) and triethylamine (0.01 mol) in 5 mL of tetrachloromethane was added dropwise slowly with constant stirring and cooling at 5 °C. The reaction mixture was stirred for 2 h at room temperature under an argon atmosphere, after which a solution of the second amine (R^2^) (0.0025 mol) in 5 mL of tetrachloromethane was added dropwise. The reaction mixture was subsequently stirred at room temperature for 13–26 h. The solvent was then distilled off, the reaction mixture was extracted with tetrahydrofuran (3 × 10 mL), and the extract was concentrated. The resulting yellow oils were subsequently recrystallized from a hexane/benzene (99:1) solution. The final product obtained was white crystals.

*4-[(phenylethynyl)(pyrrolidin-1-yl)phosphoryl]morpholine* (**6e**). Colorless solid; yield 75%; mp 223–227 °C. ^1^H NMR (400 MHz, CDCl_3_): δ 1.86–1.90 (m, 4H), 3.22–3.30 (m, 8H), 3.72–3.74 (m, 4H), 7.53–7.45 (m, 3H), 7.54 (d, ^3^*J*_HH_ = 6.85 Hz, 2H). ^13^C NMR (100 MHz, CDCl_3_): δ 26.40 (d, ^3^*J*_CP_ = 8.4 Hz), 44.20, 46.13 (d, ^2^*J*_CP_ = 4.8 Hz), 67.16 (d, ^2^*J*_CP_ = 6.6 Hz), 80.90 (d, ^1^*J*_CP_ = 231.2 Hz), 100.11 (d, ^2^*J*_CP_ = 40.3 Hz), 120.42 (d, ^4^*J*_CP_ = 2.2 Hz), 126.90, 128.52, 130.15, 132.40 (d, ^2^*J*_CP_ = 2.6 Hz). ^31^P NMR (161 MHz, CDCl_3_): δ 2.44. HRMS (ESI-TOF) for C_16_H_21_N_2_O_2_P [M+H]^+^ m/z calcd 305.1341, obsd. 305.1403.

*4-[(phenylethynyl)(pyrrolidin-1-yl)phosphoryl]1-methylpiperazine* (**6f**). Colorless solid; yield 70%; mp 223–227 °C. ^1^H NMR (400 MHz, CDCl_3_): δ 1.80–1.83 (m, 4H), 2.25 (3H), 2.41–2.33 (m, 4H), 3.18–3.24 (m, 8H), 7.27–7.38 (m, 3H), 7.46 (d, ^3^*J*_HH_ = 6.85 Hz, 2H). ^13^C NMR (100 MHz, CDCl_3_): δ 14.08, 26.31 (d, ^3^*J*_CP_ = 2.93 Hz), 43.86 (d, ^3^*J*_CP_ = 2.6 Hz), 45.96 (d, ^2^*J*_CP_ = 4.8 Hz), 55.14 (d, ^2^*J*_CP_ = 6.6 Hz), 81.16 (d, ^1^*J*_CP_ = 231.3 Hz), 99.83 (d, ^2^*J*_CP_ = 40.2 Hz), 120.51 (d, ^4^*J*_CP_ = 2.1 Hz), 126.85, 128.43, 129.99, 132.30 (d, ^2^*J*_CP_ = 2.1 Hz). ^31^P NMR (161 MHz, CDCl_3_): δ 2.71. HRMS (ESI-TOF) for C_17_H_24_N_3_OP [M+H]^+^ m/z calcd 318.1657, obsd. 318.1701.

### 3.3. Antiviral Activity Evaluation

Influenza A virus (IAV, strain A/Puerto Rico/8/1934 (H1N1)) and permissive cell line MDCK (ATCC #CCL-34) were obtained from the collection of the Pasteur Institute (St. Petersburg, Russia).

MDCK cells were cultured in α-MEM medium supplemented with 100 units/mL penicillin, 100 μg/mL streptomycin, and 5% fetal bovine serum (Gibco, USA) in a CO_2_ incubator at 36 °C with 5% CO_2_. IAV was propagated in MDCK cells maintained in α-MEM medium supplemented with penicillin/streptomycin and 1 μg/mL trypsin (Sigma, Germany). Stocks of the compounds tested were dissolved in DMSO.

In experiments assessing the antiviral activity of the compounds, MDCK cells were seeded into 96-well microtiter plates at a density of 10 × 10^3^ cells per well in 100 μL of complete medium and allowed to grow and adhere to the wells for 24 h at 37 °C. Thereafter, the media were removed, and serial dilutions of compounds (300 μg/mL–3.7 μg/mL) in maintenance medium were added to the wells containing MDCK monolayers. Virus control wells received maintenance medium without test compounds. The plates were incubated for 1 h in a CO_2_ incubator at 36 °C with 5% CO_2_. After 1 h, all of the cells were infected with IAV (M.O.I. 0.01), except for the cell control wells and compound cytotoxicity control wells. The plates were incubated for 48 h in a CO_2_ incubator at 36 °C with 5% CO_2_, after which cell viability in all wells was assessed via MTT assay, and the optical density in each well (540 nm) was measured using Multiskan FC (Thermo Scientific) [59].

The cytoprotective activity of the compounds was determined by their ability to increase optical density values in wells with test compounds compared to virus control wells. Based on the obtained optical density values in the wells, the 50% inhibitory concentration (denoted as IC_50_) was calculated using GraphPad Prism software (version 6.01)—the concentration of a compound that reduces the death of infected cells by 50% compared to the virus control. Using optical density values from cytotoxicity control wells, the 50% cytotoxic concentration (denoted as CC_50_) for each compound was calculated—the concentration at which cell viability in the absence of virus is reduced by 50%. The selectivity index (SI) was calculated from the CC_50_ and IC_50_ values using the formula SI = CC_50_/IC_50_, where CC_50_ is the 50% cytotoxic concentration, IC_50_ is the 50% inhibitory concentration, and SI is the selectivity index.

### 3.4. Molecular Docking

Virtual screening of the behavior of the obtained compounds towards a number of protein targets was performed by molecular docking. Products **4a**–**g** and **6a**–**f** were drawn in ChemBioDraw and processed by the LigPrep tool integrated into Schrodinger Suite 2020-4 in the OPLS3e force field, generating possible ionization states at pH = 7 ± 0.5 and stereoisomers [60].

Protein models extracted from the RCSB Protein Data Bank were preprocessed using the Protein Preparation Wizard tool by hydrogen addition, H-bond assignment, and restrained minimization in the OPLS3e force field. The following protein models were considered: epidermal growth factor receptor (EGFR, PDB id: 4HJO) [61], platelet-derived growth factor receptor A (PDGFRA, PDB id: 6JOL), B-Raf proto-oncogene (PDB id: 5CSW) [62], focal adhesion kinase (FAK, PDB id: 2IJM), AMP-activated protein kinase (AMPK, PDB id: 7JHG) [63], dual-specificity tyrosine-phosphorylation-regulated kinase 1A (DYRK1A, PDB id: 7O7K) [64], dual-specificity tyrosine-phosphorylation-regulated kinase 1B (DYRK1B, PDB id: 8C2Z), Janus kinase 1 (JAK1, PDB id: 6N7A) [65], Janus kinase 2 (JAK2, PDB id: 8EX1) [66], homeodomain interacting protein kinase 2 (HIPK2, PDB id: 6P5S) [67], homeodomain interacting protein kinase 3 (HIPK3, PDB id: 7O7J) [64], cyclin-dependent kinase 4 (CDK4, PDB id: 7SJ3) [68], cyclin-dependent kinase 7 (CDK7, PDB id: 8S0T) [69], casein kinase 2 alpha 1 (CSNK2A1, PDB id: 3PE1) [70], casein kinase 2 alpha 2 (CSNK2A2, PDB id: 6HMB) [71], human myosin light chain kinase 4 (MYLK4, PDB id: 2X4F), haploid germ cell–specific nuclear protein kinase (Haspin, PDB id: 3IQ7) [72], proto-oncogene protein kinase Pim-1 (PDB id: 4XHK), proto-oncogene protein kinase Pim-2 (PDB id: 4X7Q) [73], tyrosine kinase 2 (TYK2, PDB id: 6NZP) [74], Aurora kinase B (AURKB, PDB id: 4AF3) [75], phosphodiesterase type 5 (PDE5, PDB id: 4MD6) [76], metabotropic glutamate receptor 3 (mGluR3, PDB id: 8TR0) [77], multidrug resistance protein 1 (MDR1, PDB id: 7A65) [78], poly (ADP-ribose) polymerase 1 (PARP1, PDB id: 7KK4) [79], poly (ADP-ribose) polymerase 2 (PARP1, PDB id: 4TVJ) [80], poly (ADP-ribose) polymerase 3 (PARP3, PDB id: 4GV0) [81], transforming growth factor-β (TGF-β, PDB id: 1PY5) [82], influenza surface glycoprotein hemagglutinin (HA, PDB id: 3EYM) [83], Zike virus RNA-dependent RNA polymerase (RdRp, PDB id: 6LD3) [84], COVID-19 main protease (Mpro, PDB id: 6LU7) [85], and beta-actin (PDB id: 8DNH) [86].

Receptor grids were generated based on the ligand position in the considered protein complexes, except for MDR1, for which a previously reported binding site was used [87]. Calculations were performed using the Glide program in flexible docking mode with the standard precision (SP) algorithm, recording five poses per ligand [88]. To assess the affinity of the synthesized compounds for the considered targets, the Docking Score scoring function expressed in kcal/mol was used. The Maestro graphical user interface was used to launch the calculations and visualize the results.

### 3.5. Cell Culture and Culturing Conditions

The human cervical carcinoma (HeLa) and erythroleukemia (K-562) cell lines were obtained from the Bank of Cell Cultures of the Institute of Cytology of the Russian Academy of Sciences. The human melanoma (Sk-mel-2) cell line was obtained from the Bank of Cell Cultures of the Institute of Cytology and Genetics, Siberian Branch of Russian Academy of Sciences. K-562 cells were cultured in RPMI medium (Hyclone) supplemented with fetal bovine serum (FBS, 10% *v*/*v*, Hyclone) and gentamicin. HeLa and Sk-mel-2 cells were cultured in Dulbecco’s Modified Eagle’s Medium (DMEM) with the same supplements. All the cell lines were maintained under controlled conditions: a humid atmosphere with 5% CO_2_ at 37 °C.

### 3.6. Cell Proliferation Assay

Cell viability was measured in vitro using the 3-(4,5-dimethylthiazol-2-yl)-5-(3-carboxymethoxyphenyl)-2-(4-sulfophenyl)-2*H*-tetrazolium (MTS) assay. In short, cells were seeded into 96-well microtiter plates at a density of 5 × 10^3^ cells per well in 100 μL of complete medium and allowed to grow and adhere to the wells for 24 h at 37 °C. Then, the cells were treated with various concentrations of the compounds for a period of 24 or 72 h. After treatment, 20 μL of MTS reagent stock solution was added to each well, and the plates were incubated at 37 °C for 2 h in a humidified, 5% CO_2_ atmosphere. Finally, the absorbance was recorded at 495 nm using a 96-well plate reader ‘Multiskan GO’. All samples were measured in triplicate.

### 3.7. Actin Cytoskeleton Staining

Sk-mel-2 or HeLa cells were seeded into a Petri dish with cover slips at a density of 2 × 10^5^ cells per dish and incubated for 24 h. Then, the cells were treated with the chosen compounds (10 μg/mL) for 24 h. The medium was removed, and the cells were fixed with 4% paraformaldehyde, washed with PBS three times, and permeabilized with 0.3% Triton-X100. The cells were then rinsed with PBS three times. Actin filaments (microfilaments) were stained at 37 °C for 15 min with rhodamine-phalloidin. The samples were rinsed with PBS three times, followed by embedding in Fluoroshield medium. The intensity of the staining of the preparations was estimated using an AxioObserver Z1 confocal microscope.

### 3.8. Evaluation of Cell Motility by Scratch Test

Cells were seeded into Petri dishes at a density of 5 × 10^5^ cells per dish and grown to confluency. Scratch wounds were made using a 200 μL pipette tip, and detached cells were removed by washing with PBS. The culture media was replaced with serum-free DMEM in order to inhibit cell proliferation. The compounds to be screened were added to the cultures at a 10 μg/mL concentration and incubated for 24 h. Different fields were analyzed under a bright field, and each scratch area was photographed at 0 and 24 h. Images were captured using an Axio Observer Z1 confocal microscope (Carl Zeiss MicroImaging GmbH, Jena, Germany). The percent of wound closure in five randomly chosen fields was calculated with NIH ImageJ software.

## 4. Conclusions

In this work, a series of novel acetylene-containing phosphonates was successfully obtained via selective, catalyst-free synthesis based on 2-phenylethynylphosphonic dichloride. The structures of the obtained products resulting from nucleophilic substitution of 2-phenylethynylphosphonic dichloride with aliphatic amines and alcohols were confirmed by NMR spectroscopy and high-resolution mass spectrometry. Additionally, single-crystal X-ray diffraction analysis was performed for compound **6c**.

The results of biological studies of the synthesized series revealed a clear structure–activity relationship. While dialkylphosphonates **4a–g** exhibited pronounced cytotoxicity in the studied cancer cell lines and generally lacked selectivity for the influenza virus, diamide derivatives **6a–f** demonstrated a more favorable profile in terms of antiviral activity. In particular, compound 6b proved to be promising candidates, with low cytotoxicity (CC50 > 300 μg/mL) and moderate activity (IC_50_ = 56.9 μg/mL) against influenza A (H1N1) virus, resulting in a selectivity index of 5.

Further antiproliferative assays identified the butyl, isobutyl, and hexyl esters **4d**, **4e**, and **4g** as the most potent analogs, with IC_50_ values as low as 6 μg/mL in K562 erythroleukemia cells. The most potent antiproliferative compounds from the dialkylphosphonate series demonstrated the ability to disrupt actin cytoskeleton organization, leading to a significant loss of stress fibers and reduced cell motility, as confirmed by confocal microscopy and wound-healing assays.

Virtual screening of the synthesized compounds for the most popular anticancer targets demonstrated differing patterns towards kinase and nonkinase protein targets, while indicating the applicability of the molecular docking method for proposing the target and performing further optimization. Collectively, the obtained results provide a solid basis for the targeted design and optimization of novel, more potent, and selective anticancer agents based on acetylenic phosphonates.

## Figures and Tables

**Table 1:** Calculated physicochemical characteristics of **4a**–**g** and **6a**–**f**. Values outside the optimal range are highlighted in bold.

Compound	XLOGP3	MW	TPSA	ESOL Log S	Fraction Csp^3^	RB
**4a**	2.56	210.17	45.34	0.928	**0.20**	2
**4b**	3.34	238.22	45.34	0.363	0.33	4
**4c**	4.12	266.27	45.34	0.084	0.43	4
**4d**	4.51	280.30	45.34	0.053	0.47	7
**4e**	4.61	294.33	45.34	0.019	0.50	6
**4f**	4.90	294.33	45.34	0.038	0.50	4
**4g**	**6.46**	350.43	45.34	0.002	0.60	**12**
**6a**	2.70	288.32	33.36	0.074	0.50	2
**6b**	1.17	320.32	51.82	0.664	0.50	2
**6c**	0.24	346.41	39.84	0.301	0.56	2
**6d**	1.02	374.46	39.84	0.106	0.60	4
**6e**	1.93	304.32	42.59	0.223	0.50	2
**6f**	1.47	317.37	36.60	0.150	0.53	2

**Table 2:** Virtual screening results represented by the Docking Scores (kcal/mol) for **4a–g** towards anticancer protein targets. Maximum calculated activity values (minimum Docking Scores) are highlighted in gray.

Entry	Target	Ligand						
		4a	4b	4c	4d	4e	4f	4g
1	EGFR	−6.2	−6.2	−5.5	−4.4	−5.6	−5.6	−4.4
2	PDGFRA	−7.5	−7.6	−8.2	−5.3	−6.1	−5.7	−7.1
3	B-RAF	−6.2	−6.2	−5.5	−4.4	−5.6	−5.6	−4.4
4	FAK	−5.3	−5.4	−5.5	−3.6	−5.0	−5.9	−4.1
5	AMPK	−6.1	−5.5	−5.6	−3.5	−5.2	−5.1	−3.6
6	DYRK1A	−6.3	−5.8	−5.2	−3.5	−5.6	−6.3	−5.0
7	DYRK1B	−6.8	−6.9	−6.7	−5.1	−7.3	−6.6	−6.7
8	JAK1	−6.4	−5.9	−5.3	−2.6	−5.1	−5.2	−4.5
9	JAK2	−6.2	−5.5	−6.3	−4.2	−6.1	−6.1	−5.8
10	HIPK2	−6.4	−5.9	−6.3	−4.2	−5.8	−6.5	−5.7
11	HIPK3	−6.6	−6.5	−6.8	−4.1	−6.2	−6.9	−5.5
12	CDK4	−5.8	−5.6	−5.8	−3.7	−4.9	−6.0	−5.2
13	CDK7	−4.8	−4.9	−5.0	−2.8	−4.2	−5.1	−3.9
14	CSNK2A1	−6.1	−6.0	−6.6	−4.6	−5.6	−6.9	−5.6
15	CSNK2A2	−6.3	−6.1	−6.4	−4.1	−5.6	−6.8	−5.7
16	MYLK4	−6.8	−6.5	−5.9	−3.5	−5.1	−5.4	−4.3
17	HASPIN	−5.6	−5.7	−5.2	−3.5	−5.2	−5.5	−4.3
18	PIM1	−6.4	−6.1	−6.4	−5.3	−5.5	−5.1	−4.8
19	PIM2	−6.3	−5.6	−5.4	−3.8	−4.8	−5.4	−4.4
20	TYK2	−6.5	−5.8	−6.0	−3.0	−5.1	−5.1	−5.2
21	AURKB	−6.3	−5.9	−6.0	−4.0	−5.5	−5.6	−4.8
22	PDE5	−5.5	−4.4	−4.7	−3.8	−4.0	−4.7	−3.7
23	mGluR3	−5.6	−4.4	−4.6	−2.8	−4.6	−4.6	−4.6
24	MDR1	−6.0	−5.6	−5.8	−3.8	−4.7	−5.3	−5.5
25	PARP1	−6.0	−5.5	−6.2	−4.6	−6.1	−5.9	−5.8
26	PARP2	−6.4	−6.1	−6.4	−4.4	−5.7	−6.0	−6.5
27	PARP3	−6.9	−6.8	−7.0	−5.0	−6.5	−7.4	−6.6
28	TGF-*β*	−5.6	−5.4	−6.2	−3.8	−5.8	−5.6	−5.2
29	HA	−5.6	−5.2	−5.7	−3.9	−5.5	−5.6	−5.7
30	RdRp	−6.3	−6.3	−6.5	−4.2	−6.3	−5.9	−5.7
31	MPro	−3.7	−3.4	−3.1	−0.9	−3.1	−3.4	−2.5
32	Actin	−6.0	−5.5	−4.2	−2.8	−5.1	−4.4	−4.7

**Table 3:** Virtual screening results represented by Docking Scores (kcal/mol) for **6a–f** towards anticancer protein targets. Maximum calculated activity values (minimum Docking Scores) are highlighted in gray.

Entry	Target	Ligand					
		6a	6b	6c	6d	6e	6f
1	EGFR	−5.4	−5.5	−5.0	−5.2	−6.2	−5.4
2	PDGFRA	−8.5	−7.6	−7.3	−7.1	−8.1	−6.4
3	B-RAF	−5.5	−5.6	−4.8	−5.2	−6.2	−5.4
4	FAK	−5.9	−5.8	−6.3	−6.3	−6.0	−6.1
5	AMPK	−4.5	−4.8	−4.9	−5.2	−4.9	−5.6
6	DYRK1A	−6.2	−6.4	−5.9	−6.0	−6.5	−6.8
7	DYRK1B	−6.7	−7.6	−7.2	−6.4	−7.3	−7.5
8	JAK1	−4.9	−5.2	−5.5	−5.5	−5.2	−5.9
9	JAK2	−6.2	−6.3	−5.4	−5.5	−6.3	−6.0
10	HIPK2	−6.4	−6.4	−6.5	−6.3	−6.5	−6.3
11	HIPK3	−7.1	−6.4	−7.4	−7.2	−7.3	−7.3
12	CDK4	−6.1	−6.3	−6.9	−6.6	−6.4	−6.5
13	CDK7	−5.1	−5.3	−5.8	−5.7	−5.4	−5.9
14	CSNK2A1	−5.8	−6.3	−5.2	−5.4	−5.9	−6.1
15	CSNK2A2	−7.0	−6.6	−5.8	−6.3	−6.5	−6.6
16	MYLK4	−6.5	−5.4	−6.3	−6.2	−5.9	−6.2
17	HASPIN	−6.4	−5.6	−7.4	−6.9	−6.0	−7.0
18	PIM1	−5.4	−6.2	−6.7	−6.8	−5.9	−7.2
19	PIM2	−5.4	−6.1	−6.8	−6.3	−5.6	−6.8
20	TYK2	−5.0	−4.6	−6.0	−5.8	−5.6	−5.6
21	AURKB	−6.4	−6.3	−5.8	−5.6	−6.5	−7.0
22	PDE5	−6.3	−5.7	−6.6	−6.6	−6.6	−6.5
23	mGluR3	−4.1	−4.5	−4.1	−4.3	−4.4	−4.6
24	MDR1	−6.2	−5.9	−6.2	−5.9	−6.2	−6.2
25	PARP1	−5.9	−6.4	−6.6	−7.4	−6.6	−6.7
26	PARP2	−6.0	−6.5	−6.5	−6.5	−6.5	−6.9
27	PARP3	−6.9	−6.9	−6.6	−6.8	−7.7	−7.1
28	TGF-*β*	−6.9	−7.9	−6.1	−6.4	−6.5	−6.9
29	HA	−6.4	−5.9	−8.1	−7.7	−6.7	−7.3
30	RdRp	−5.8	−5.6	−4.9	−4.8	−5.8	−5.0
31	MPro	−2.6	−3.1	−3.2	−3.0	−3.4	−3.4
32	Actin	−4.8	−4.5	−4.7	−5.6	−5.0	−4.8

**Table 4:** Cytotoxic and antiviral properties of **4a**–**g** and **6a**–**f** against the virus A/PR/8/34 (H1N1) in MDCK cells. The compound with the maximum selectivity index is highlighted in gray.

Compound	CC_50_, μM ^1^	IC_50_, μM ^2^	SI ^3^
**4a**	58.0	>33	<1
**4b**	213.1	>100	<2
**4c**	59.9	>33	<2
**4d**	13.7	>11	<1
**4e**	13.1	>11	<1
**4f**	>300	>100	<3
**4g**	4.1	>3	<1
**6a**	56.2	53	1
**6b**	>300	56.9	5
**6c**	>300	>300	1
**6d**	39.8	>33	1
**6e**	>300	140	2
**6f**	>300	71.7	4

^1^ CC_50_—50% cytotoxic concentration. ^2^ IC_50_—50% inhibitory concentration. ^3^ SI—Selectivity index, defined as SI = CC_50_/IC_50_.

**Table 5:** IC_50_ values for compounds **4a–g** and **6a–f** against the K562, HeLa, and Sk-mel-2 cell lines.

Compound	IC_50_, μg/mL					
	K562		HeLa		Sk-mel-2	
	24 h	72 h	24 h	72 h	24 h	72 h
**4a**	>40	>40	>40	>40	>40	>40
**4b**	>40	>40	>40	>40	>40	>40
**4c**	>40	21 ± 3	>40	>40	36 ± 4	35 ± 4
**4d**	26 ± 2	6 ± 2	13 ± 1	13 ± 3	27 ± 4	27 ± 3
**4e**	18 ± 2	6 ± 1	10 ± 1	20 ± 3	12 ± 3	14 ± 3
**4f**	>40	>40	>40	>40	>40	>40
**4g**	18 ± 3	6 ± 1	14 ± 3	15 ± 3	32 ± 4	26 ± 3
**6a**	>40	>40	>40	>40	>40	>40
**6b**	>40	>40	>40	>40	>40	>40
**6c**	>40	>40	>40	>40	>40	>40
**6d**	>40	40 ± 5	>40	>40	>40	>40
**6e**	>40	>40	>40	>40	>40	>40
**6f**	>40	>40	>40	>40	>40	>40
**Cisplatin**	24 ± 4	5 ± 1	16 ± 4	6 ± 1	24 ± 4	5 ± 1
**Doxorubicin**	16 ± 2	1 ± 0.5	4 ± 1	0.5± 0.1	3 ± 1	0.6± 0.1

**Table 6:** SI values for the most promising anticancer compounds **4c–g**.

Compound	HEK293T	MDCK	HeLa	SI		K562	SI		Sk-mel-2	SI	
				HEK293T	MDCK		HEK293T	MDCK		HEK293T	MDCK
**4c**	39	-	>40	<1	-	21.3	1.8	-	34.8	1.1	-
**4d**	49	26.5	12.7	3.8	2.1	6.3	7.8	4.2	27.2	1.8	1.0
**4e**	10	28.2	19.7	0.5	1.4	5.6	1.8	5.0	14.3	0.7	2.0
**4g**	110	7.1	15.1	7.3	0.5	5.7	19.3	1.2	25.6	4.3	0.3

## Data Availability

The original contributions presented in this study are included in the article/Supplementary Material. Further inquiries can be directed to the corresponding authors.

## Supplementary Materials

The following supporting information can be downloaded at: https://www.mdpi.com/article/10.3390/molecules31111861/s1, Figures S1–S39: ^31^P, ^13^C and ^1^H NMR spectra of compounds **4a–g**, **6a–f**; Figures S40–S52: HR ESI–MS spectra of compounds **4a–g**, **6a–f**; Figures S53–S65: HPLC chromatograms of compounds **4a–g**, **6a–f**; Figure S66: Wound-healing ability of Sk-mel-2 cells after incubation with compounds **4c**, **4d**, **4e**, and **4g**; Figure S67: Wound-healing ability of HeLa cells after incubation with compounds **4d**, **4e**, and **4g**; Figure S68–S80: The Bioavailability Radar for compounds **4a–g**, **6a–f**; Table S1: Crystal data and structure refinement for **6c**; Table S2. Fractional atomic coordinates and equivalent isotropic displacement parameters for **6c**; Table S3: Anisotropic displacement parameters for **6c**; Table S4: Bond lengths for **6c**; Table S5: Bond angles for **6c**; Table S6: Torsion angles for **6c**; Table S7: Hydrogen atom coordinates and isotropic displacement parameters for **6c**; Table S8: Retention time of compounds and chromatographic conditions; Table S9: Docking Score values for redocking of the ligand to the corcystalized protein model.
