# Supplementary material for: Synthesis of Novel Acetylene-Containing Phosphonates, Their Antiviral Activity, and Their Cytotoxicity to Different Cancer Cell Lines
Source: Molecules. 2026 May 28;31(11):1861. doi: 10.3390/molecules31111861 (PMC13257472; doi:10.3390/molecules31111861)
Supplement: Supplementary file 1 [file molecules-31-01861-s001.zip › molecules-4297799-supplementary.pdf]

Supplementary Materials

Table of Experimental Data

Section	Description	Pages
1	X-ray data	S2–S6
2	NMR data	S7–S26
3	HRMS (ESI-TOF) data	S27–S33
4	HPLC analysis	S34–S38
5	Wound-healing ability (scratch-test)	S39–S40
6	ADMET prediction	S41–S47
7	Molecular docking	S48

1. X-ray data

Table S1. Crystal data and structure refinement for **6c**.

Identification code	3
Empirical formula	$C_{18}H_{27}N_4OP$
Formula weight	346.40
Temperature/K	100(2)
Crystal system	orthorhombic
Space group	$P2_12_12_1$
a/Å	10.94220(10)
b/Å	11.82670(10)
c/Å	13.90420(10)
α/°	90
β/°	90
γ/°	90
Volume/Å^3	1799.34(3)
Z	4
ρ_{calc}g/cm^3	1.279
μ/mm^{-1}	1.448
F(000)	744.0
Crystal size/mm^3	0.22 × 0.2 × 0.18
Radiation	Cu Kα (λ = 1.54184)
2Θ range for data collection/°	9.818 to 138.176
Index ranges	$-13 \leq h \leq 13$, $-14 \leq k \leq 14$, $-16 \leq l \leq 16$
Reflections collected	9643
Independent reflections	3356 [R_{int} = 0.0306, R_{sigma} = 0.0303]
Data/restraints/parameters	3356/0/220
Goodness-of-fit on F^2	1.036
Final R indexes [I>=2σ (I)]	R_1 = 0.0268, wR_2 = 0.0706
Final R indexes [all data]	R_1 = 0.0275, wR_2 = 0.0721
Largest diff. peak/hole / e Å^{-3}	0.19/-0.26
Flack parameter	0.011(11)

Table S2. Fractional atomic coordinates (×10^4) and equivalent isotropic displacement parameters (Å^2×10^3) for **6c.** U_{eq} is defined as 1/3 of the trace of the orthogonalised U_{IJ} tensor.

**Atom**	***x***	***y***	***z***	**U(eq)**
P1	2526.8(4)	3551.8(4)	4744.1(3)	13.98(14)
O1	1920.0(13)	3240.2(12)	5657.1(10)	20.2(3)
N1	3718.8(15)	4405.2(13)	4776.8(12)	16.6(3)
N2	5665.8(15)	5976.2(14)	5044.7(12)	18.4(4)
N3	1578.8(15)	4214.1(14)	4019.7(12)	16.2(3)
N4	34.0(15)	5749.3(15)	3016.6(12)	17.6(4)
C1	4953.9(17)	4005.6(16)	5026.2(14)	18.7(4)
C2	5897.5(18)	4838.7(15)	4660.5(15)	18.2(4)
C3	4456.6(18)	6346.8(16)	4744.0(15)	19.9(4)
C4	3502.4(18)	5554.2(16)	5147.8(15)	19.2(4)
C5	1941.7(18)	4649.0(17)	3078.0(14)	17.6(4)
C6	1367.6(17)	5803.1(17)	2912.9(14)	17.3(4)
C7	-295.1(18)	5320.7(17)	3968.5(14)	18.0(4)
C8	252.6(17)	4158.8(16)	4137.5(15)	15.9(4)
C9	3095.5(18)	2345.9(16)	4141.4(14)	16.8(4)
C10	3332.3(17)	1456.3(17)	3764.7(14)	17.2(4)
C11	3584.7(18)	363.8(16)	3348.5(13)	16.1(4)
C12	2617.7(19)	-285.9(16)	2986.2(14)	19.1(4)
C13	2860(2)	-1339.3(17)	2587.0(14)	20.9(4)
C14	4041(2)	-1757.6(17)	2558.9(14)	21.1(4)
C15	4998.5(19)	-1124.7(17)	2934.0(14)	19.9(4)
C16	4781.6(19)	-54.1(17)	3318.2(14)	17.4(4)
C17	6607(2)	6755.1(18)	4726.5(18)	27.1(5)
C18	-500(2)	6864.9(18)	2855.1(16)	24.0(5)

Table S3. Anisotropic displacement parameters ($Å^2×10^3$) for **6c.** The anisotropic displacement factor exponent takes the form: $-2\pi^2[h^2a^{*2}U_{11}+2hka^*b^*U_{12}+\ldots]$.

Atom	U_{11}	U_{22}	U_{33}	U_{23}	U_{13}	U_{12}
P1	13.5(2)	12.2(2)	16.2(2)	0.21(16)	0.58(19)	1.04(18)
O1	21.9(7)	19.8(7)	19.0(7)	2.6(5)	2.1(6)	2.0(6)
N1	16.0(8)	12.8(7)	21.1(8)	-1.4(7)	-0.6(7)	2.1(6)
N2	16.9(8)	15.1(8)	23.3(8)	-4.3(6)	-0.1(7)	-2.3(6)
N3	12.8(7)	17.9(8)	18.0(8)	2.4(6)	2.0(6)	1.4(6)
N4	17.5(8)	15.6(8)	19.6(8)	2.5(6)	-0.9(7)	2.6(6)
C1	15.1(9)	16.0(9)	25.1(10)	2.4(8)	-4.0(8)	1.6(7)
C2	16.4(9)	15.1(9)	23.0(10)	-1.6(8)	-0.7(8)	0.7(8)
C3	21.8(10)	13.5(9)	24.5(10)	-2.8(8)	-1.9(8)	1.5(7)
C4	17.2(9)	16.2(9)	24.3(10)	-5.5(8)	-0.5(8)	3.3(8)
C5	15.7(9)	20.2(9)	17.0(9)	0.9(8)	2.6(8)	1.8(8)
C6	18.2(10)	16.9(9)	16.9(9)	1.6(7)	1.3(8)	-2.5(8)
C7	14.7(9)	16.9(10)	22.5(10)	-1.5(8)	1.0(8)	2.0(8)
C8	12.9(9)	15.6(9)	19.3(9)	1.1(8)	1.7(8)	0.1(7)
C9	15.0(9)	16.1(9)	19.4(9)	1.5(8)	0.1(8)	-1.0(7)
C10	14.2(8)	18.6(10)	18.7(9)	2.1(8)	0.2(7)	-1.2(8)
C11	18.8(10)	14.3(9)	15.3(9)	1.5(7)	1.0(7)	0.9(8)
C12	17.4(9)	18.7(9)	21.3(9)	0.9(7)	0.6(9)	-0.6(8)
C13	25.7(10)	16.2(9)	20.9(9)	0.8(8)	-2.0(8)	-5.2(8)
C14	31.2(11)	13.5(9)	18.7(9)	0.0(8)	3.4(9)	0.4(8)
C15	20.7(10)	19.3(10)	19.5(9)	2.5(8)	4.0(8)	4.8(8)
C16	17.2(10)	17.6(9)	17.3(9)	1.2(7)	-0.5(7)	-1.3(8)
C17	24.1(11)	19.0(9)	38.0(12)	-4.4(9)	5.0(10)	-6.0(8)
C18	28.7(12)	18.8(10)	24.4(10)	2.8(8)	-2.4(9)	6.8(9)

Table S4. Bond lengths for **6c**.

Atom	Atom	Length/Å		Atom	Atom	Length/Å
P1	O1	1.4792(14)		N4	C18	1.460(3)
P1	N1	1.6498(17)		C1	C2	1.515(3)
P1	N3	1.6444(17)		C3	C4	1.511(3)
P1	C9	1.767(2)		C5	C6	1.520(3)
N1	C1	1.473(2)		C7	C8	1.517(3)
N1	C4	1.473(2)		C9	C10	1.204(3)
N2	C2	1.469(2)		C10	C11	1.442(3)
N2	C3	1.455(3)		C11	C12	1.401(3)
N2	C17	1.451(3)		C11	C16	1.400(3)
N3	C5	1.462(2)		C12	C13	1.389(3)
N3	C8	1.462(2)		C13	C14	1.385(3)
N4	C6	1.468(3)		C14	C15	1.389(3)
N4	C7	1.462(3)		C15	C16	1.395(3)

Table S5. Bond angles for **6c**.

Atom	Atom	Atom	Angle/°		Atom	Atom	Atom	Angle/°
O1	P1	N1	118.92(8)		N1	C1	C2	109.73(15)
O1	P1	N3	111.17(8)		N2	C2	C1	110.84(16)
O1	P1	C9	111.34(9)		N2	C3	C4	109.55(16)
N1	P1	C9	103.20(9)		N1	C4	C3	109.34(16)
N3	P1	N1	102.96(8)		N3	C5	C6	109.81(16)
N3	P1	C9	108.43(9)		N4	C6	C5	110.93(16)
C1	N1	P1	122.38(13)		N4	C7	C8	110.91(16)
C4	N1	P1	116.56(13)		N3	C8	C7	109.52(16)
C4	N1	C1	111.17(16)		C10	C9	P1	170.98(18)
C3	N2	C2	109.16(15)		C9	C10	C11	177.3(2)
C17	N2	C2	110.35(16)		C12	C11	C10	119.39(18)
C17	N2	C3	111.51(16)		C16	C11	C10	120.48(18)
C5	N3	P1	123.02(13)		C16	C11	C12	120.12(18)
C5	N3	C8	112.70(16)		C13	C12	C11	119.43(19)
C8	N3	P1	122.43(13)		C14	C13	C12	120.62(19)
C7	N4	C6	110.41(15)		C13	C14	C15	120.06(18)
C18	N4	C6	110.09(17)		C14	C15	C16	120.32(19)
C18	N4	C7	110.72(16)		C15	C16	C11	119.41(19)

Table S6. Torsion angles for **6c**.

A	B	C	D	Angle/°		A	B	C	D	Angle/°
P1	N1	C1	C2	159.90(14)		C5	N3	C8	C7	-56.6(2)
P1	N1	C4	C3	-155.38(14)		C6	N4	C7	C8	-58.1(2)
P1	N3	C5	C6	-139.10(15)		C7	N4	C6	C5	57.5(2)
P1	N3	C8	C7	138.54(15)		C8	N3	C5	C6	56.1(2)
O1	P1	N1	C1	79.94(17)		C9	P1	N1	C1	-43.88(17)
O1	P1	N1	C4	-62.71(16)		C9	P1	N1	C4	173.47(14)
O1	P1	N3	C5	178.20(15)		C9	P1	N3	C5	-59.09(18)
O1	P1	N3	C8	-18.47(19)		C9	P1	N3	C8	104.24(16)
N1	P1	N3	C5	49.80(17)		C10	C11	C12	C13	-179.87(18)
N1	P1	N3	C8	-146.87(16)		C10	C11	C16	C15	178.41(18)
N1	C1	C2	N2	56.4(2)		C11	C12	C13	C14	1.1(3)
N2	C3	C4	N1	-60.8(2)		C12	C11	C16	C15	-0.6(3)
N3	P1	N1	C1	-156.66(15)		C12	C13	C14	C15	0.2(3)
N3	P1	N1	C4	60.69(16)		C13	C14	C15	C16	-1.6(3)
N3	C5	C6	N4	-55.8(2)		C14	C15	C16	C11	1.9(3)
N4	C7	C8	N3	56.9(2)		C16	C11	C12	C13	-0.8(3)
C1	N1	C4	C3	57.9(2)		C17	N2	C2	C1	177.32(17)
C2	N2	C3	C4	61.6(2)		C17	N2	C3	C4	-176.21(17)
C3	N2	C2	C1	-59.8(2)		C18	N4	C6	C5	-179.97(16)
C4	N1	C1	C2	-55.7(2)		C18	N4	C7	C8	179.72(16)

Table S7. Hydrogen atom coordinates (Å×10^4) and isotropic displacement parameters (Å^2×10^3) for **6c**.

**Atom**	***x***	***y***	***z***	**U(eq)**
H1A	5097.96	3254.1	4733.83	22
H1B	5027.25	3928.88	5732.88	22
H2A	6723.33	4586.29	4856.73	22
H2B	5871.43	4861.5	3949.02	22
H3A	4407.75	6352.88	4033.06	24
H3B	4304.63	7124.73	4978.29	24
H4A	3547.18	5551.51	5858.97	23
H4B	2676.89	5813.51	4957.43	23
H5A	1672.49	4119.43	2568.83	21
H5B	2842.94	4712.13	3046.18	21
H6A	1703.92	6349.61	3382.85	21
H6B	1575.95	6073.05	2259.22	21
H7A	-1195.9	5276.17	4023.12	22
H7B	4.73	5849.88	4467.14	22
H8A	51.4	3897.19	4795.1	19
H8B	-95.71	3612.19	3673.08	19
H12	1803.71	-8.39	3013.37	23
H13	2208.39	-1776.85	2330.83	25
H14	4197.47	-2478.07	2282.94	25
H15	5804.79	-1422.48	2928.75	24
H16	5439.92	387.78	3557.29	21
H17A	7394.05	6533.72	5005.7	41
H17B	6399.94	7522.79	4935.59	41
H17C	6663.69	6734.7	4023.28	41
H18A	-1383.72	6830.55	2964.9	36
H18B	-341.24	7102.85	2191.57	36
H18C	-132.77	7410.11	3300.41	36

2. NMR data

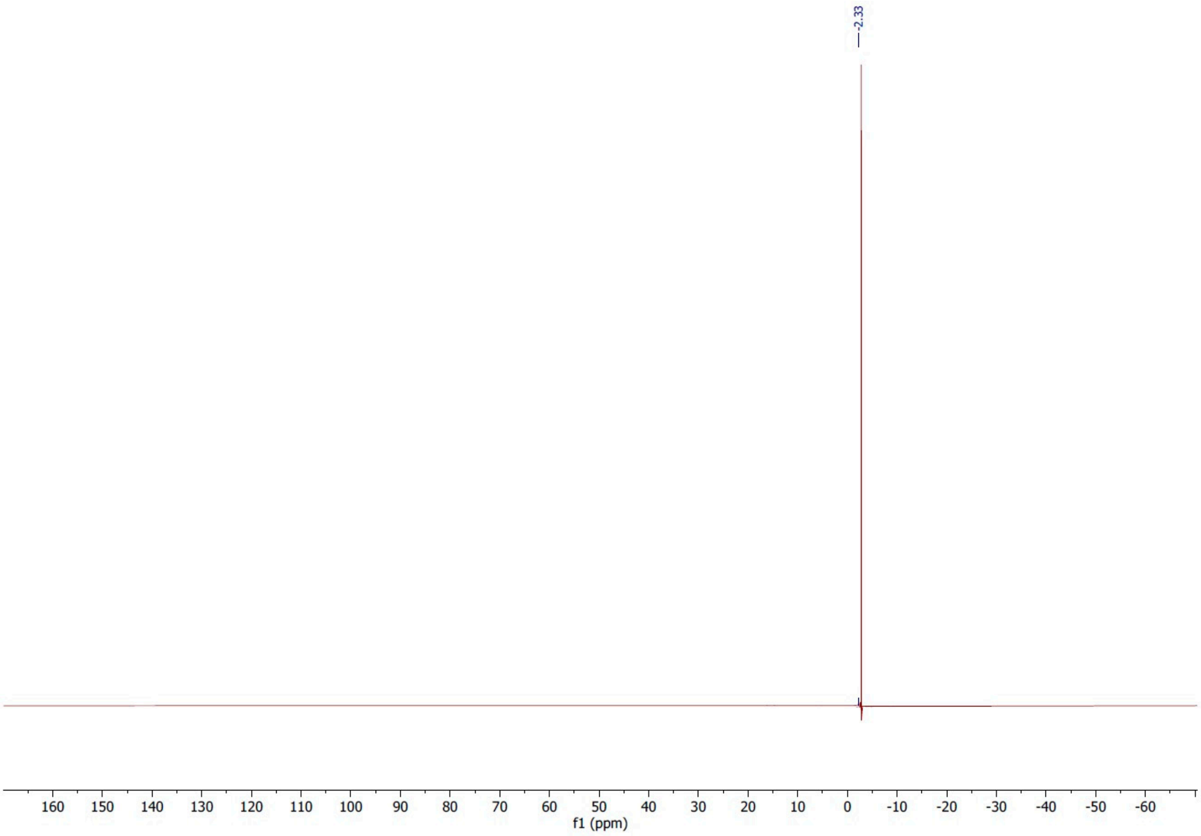

Figure S1. ^{31}P NMR spectra of dimethyl(phenylethynyl)phosphonate (**4a**).

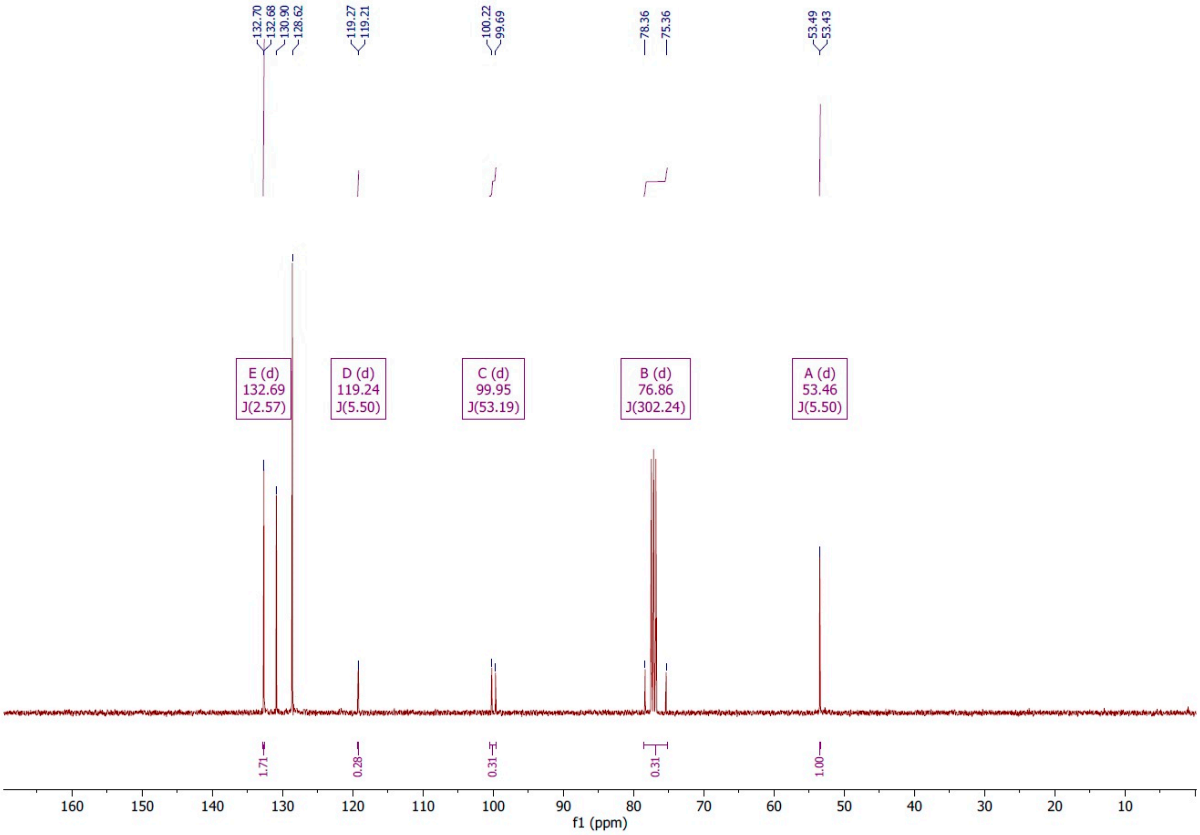

Figure S2. ^{13}C NMR spectra of dimethyl(phenylethynyl)phosphonate (**4a**).

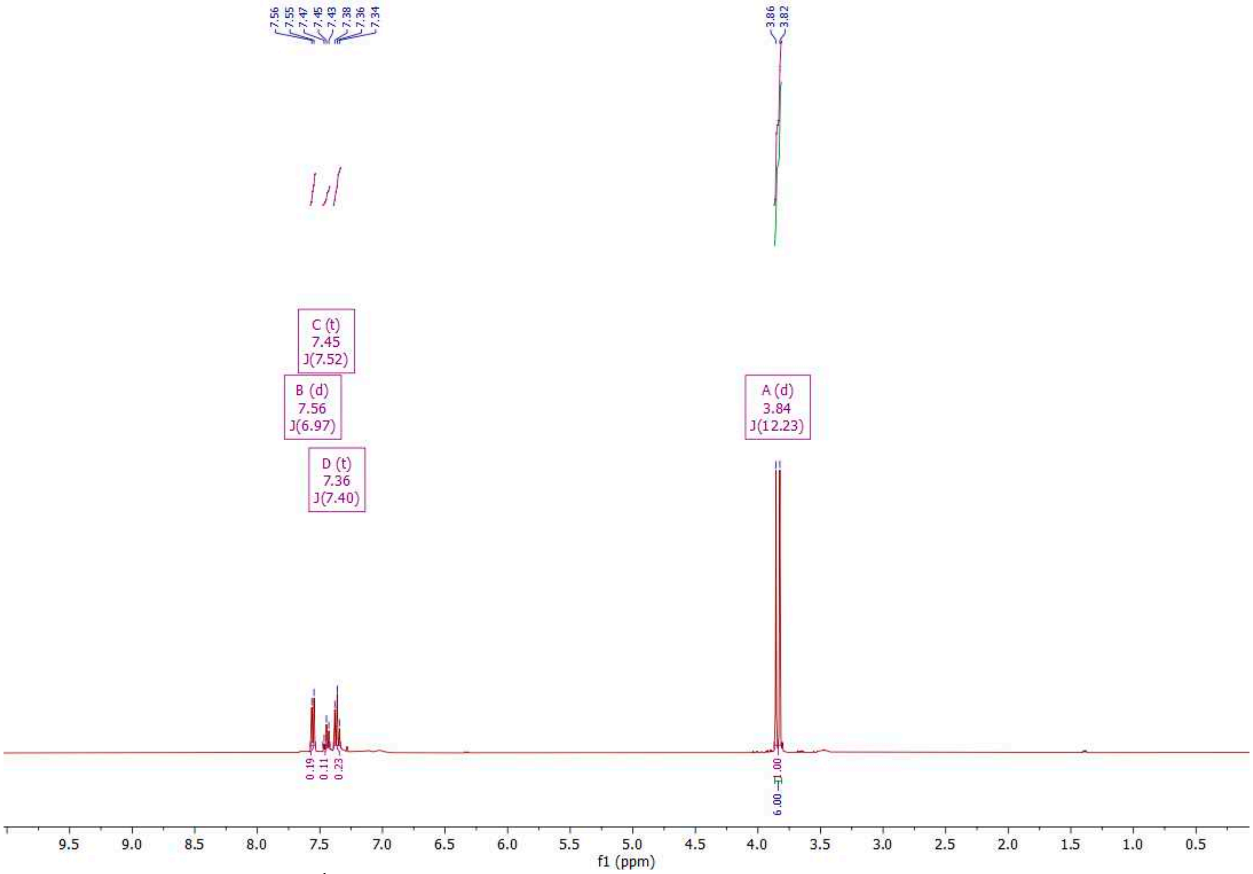

Figure S3. ^{1}H NMR spectra of dimethyl(phenylethynyl)phosphonate (**4a**).

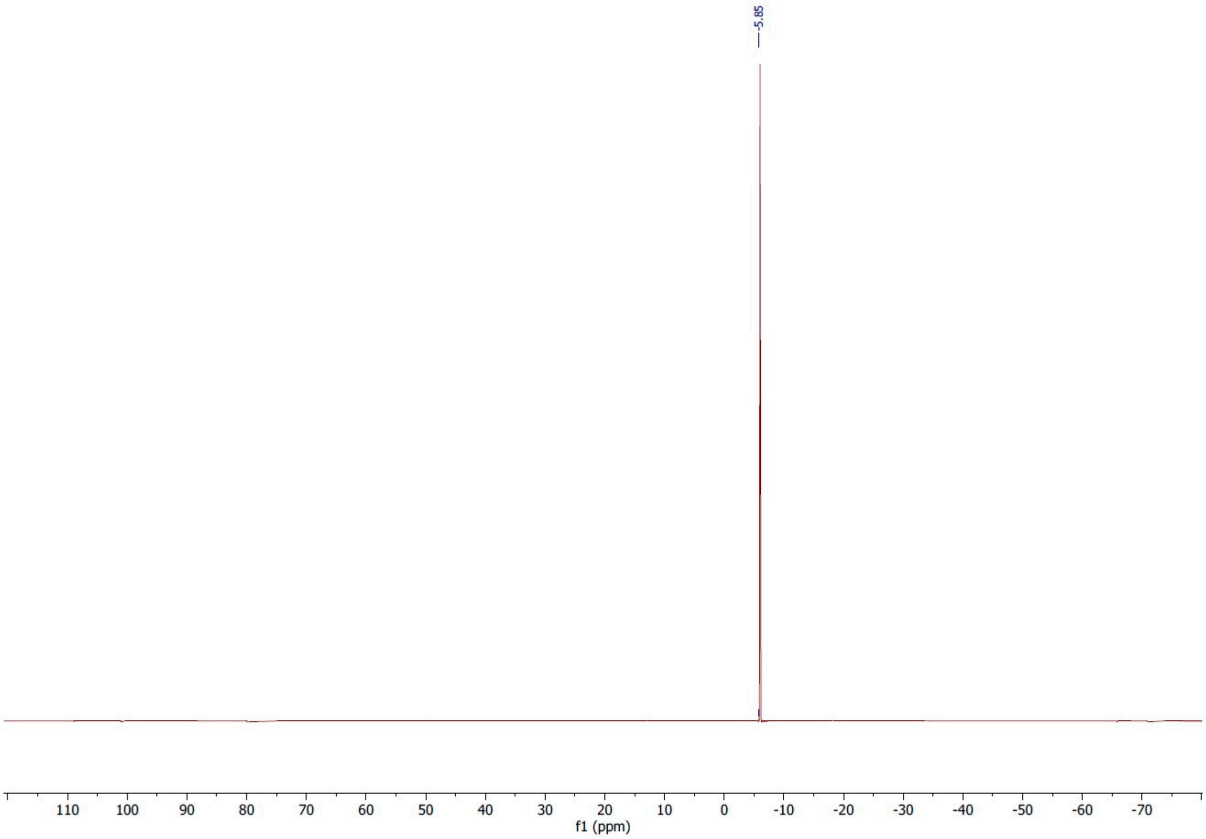

Figure S4. ^{31}P NMR spectra of diethyl(phenylethynyl)phosphonate (**4b**).

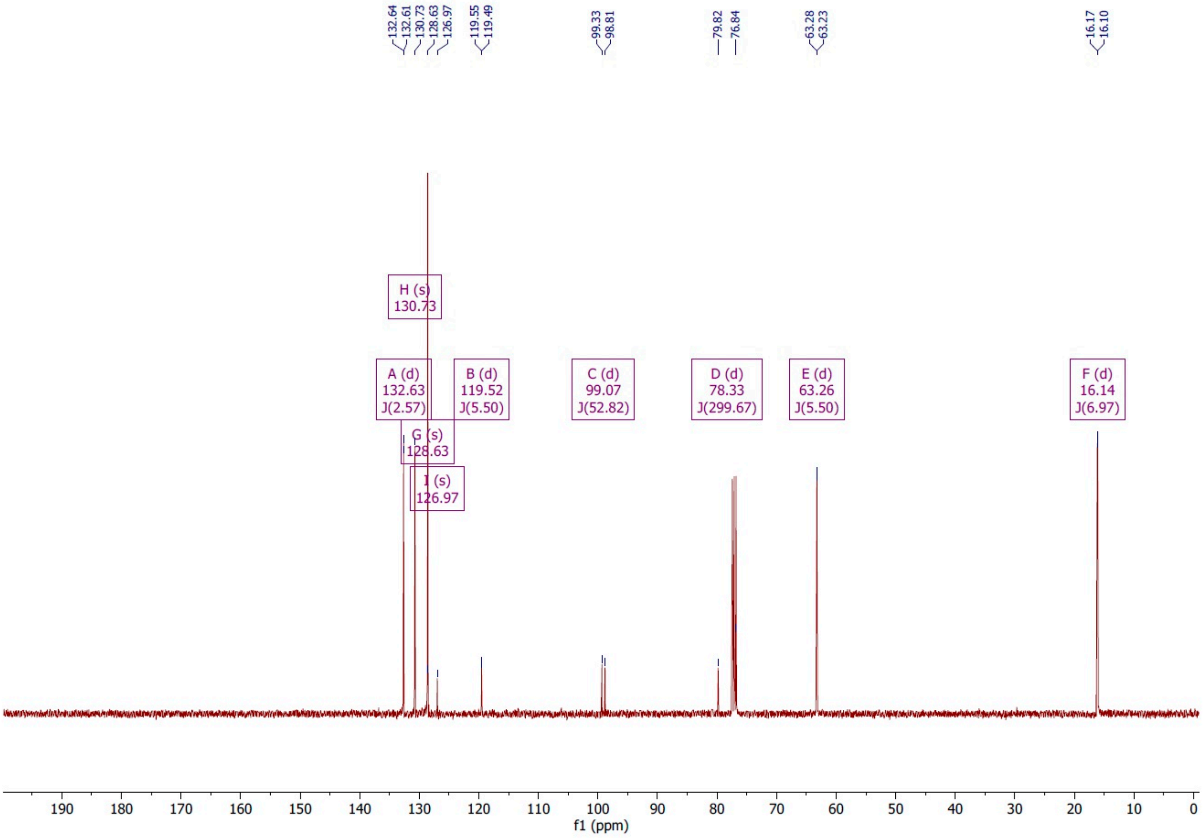

Figure S5. ^{13}C NMR spectra of diethyl(phenylethynyl)phosphonate (**4b**).

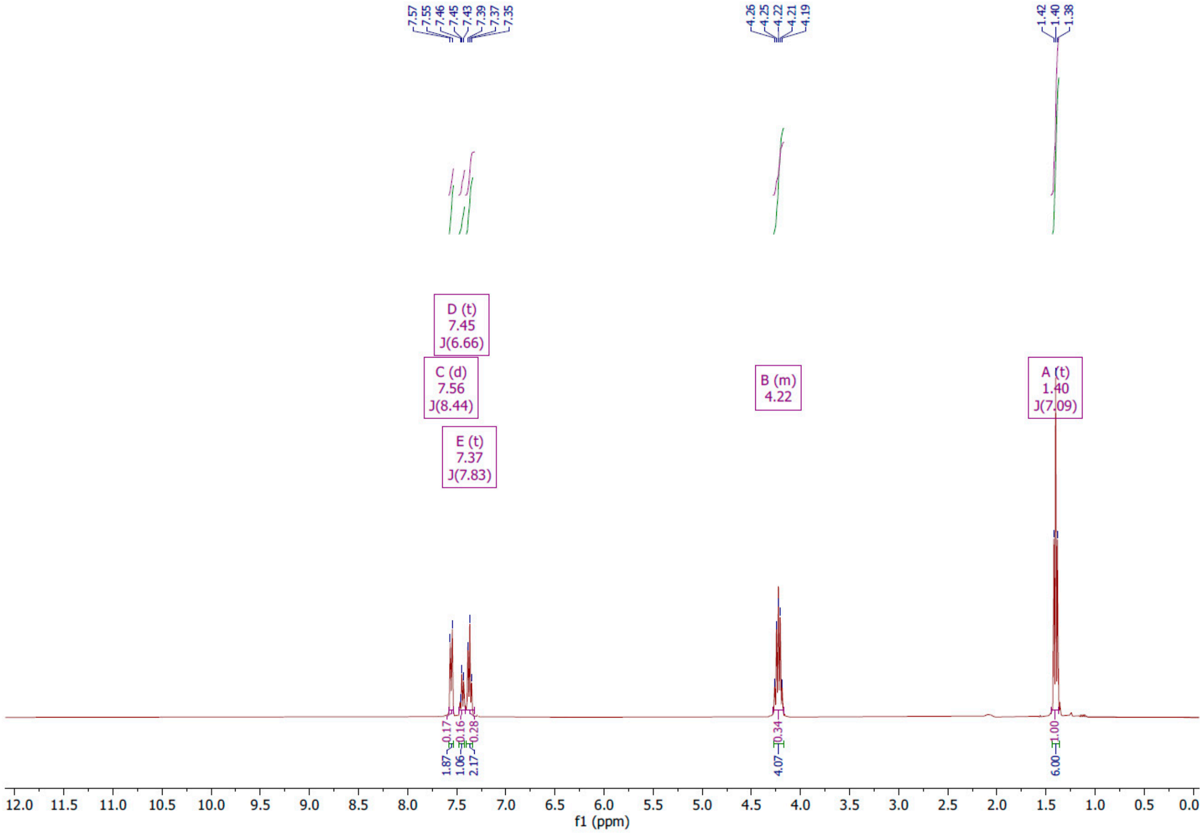

Figure S6. ^{1}H NMR spectra of diethyl(phenylethynyl)phosphonate (**4b**).

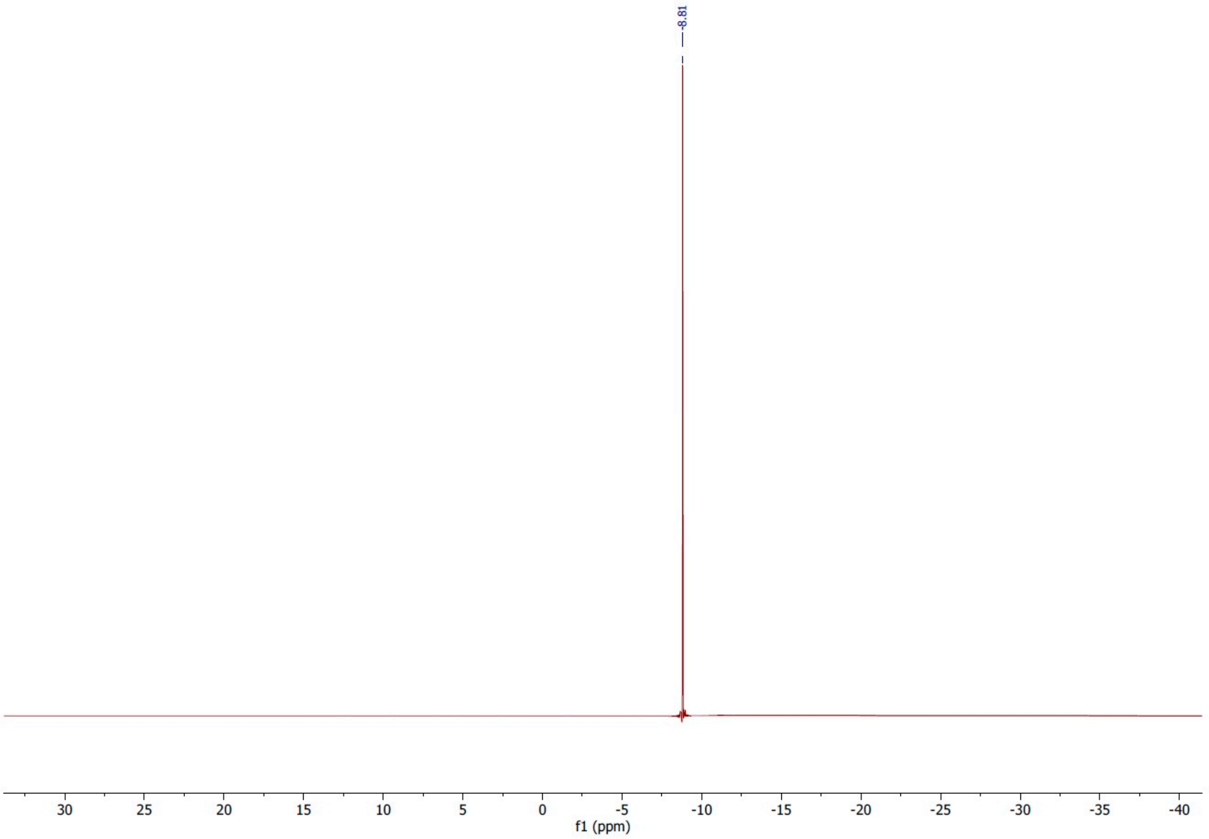

Figure S7. ^{31}P NMR spectra of diisopropyl(phenylethynyl)phosphonate (**4c**).

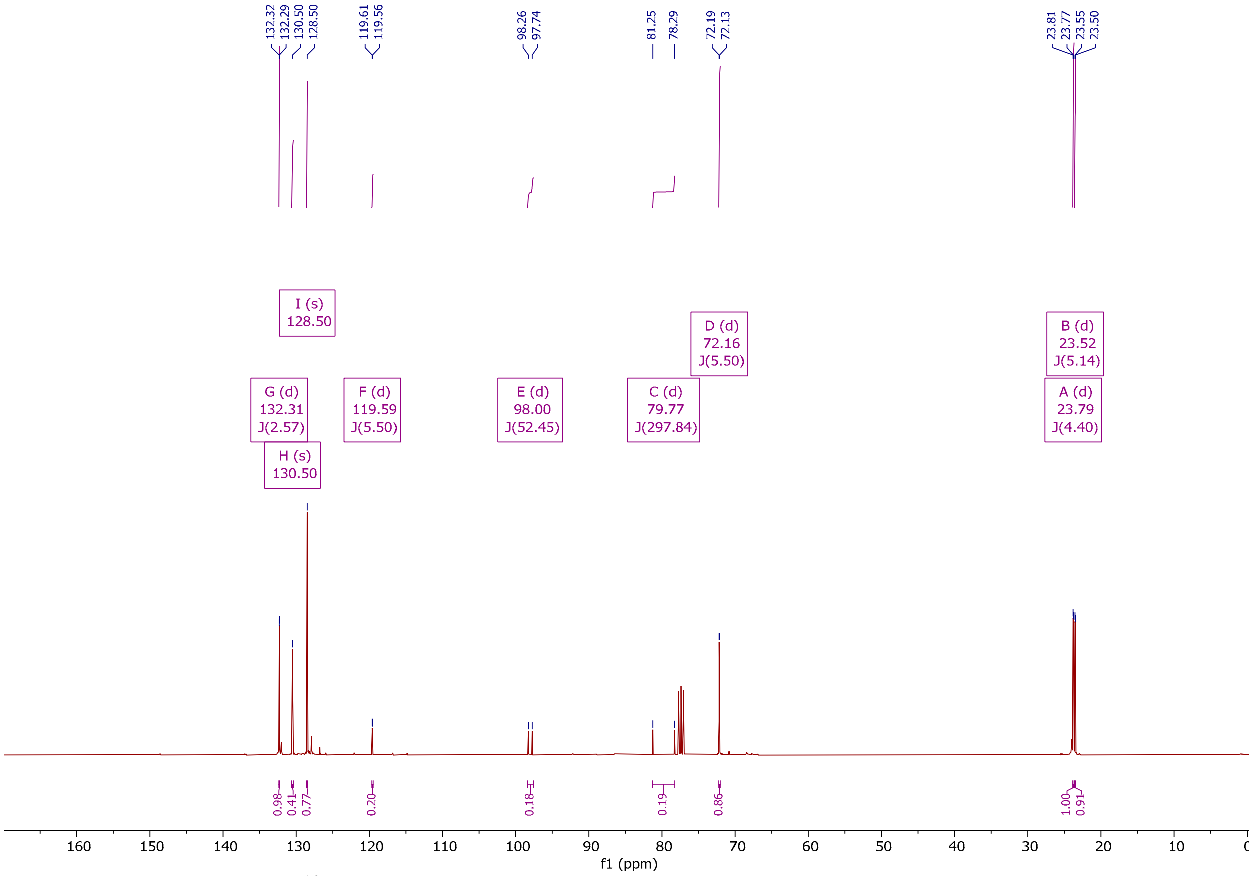

Figure S8. ^{13}C NMR spectra of diisopropyl(phenylethynyl)phosphonate (**4c**).

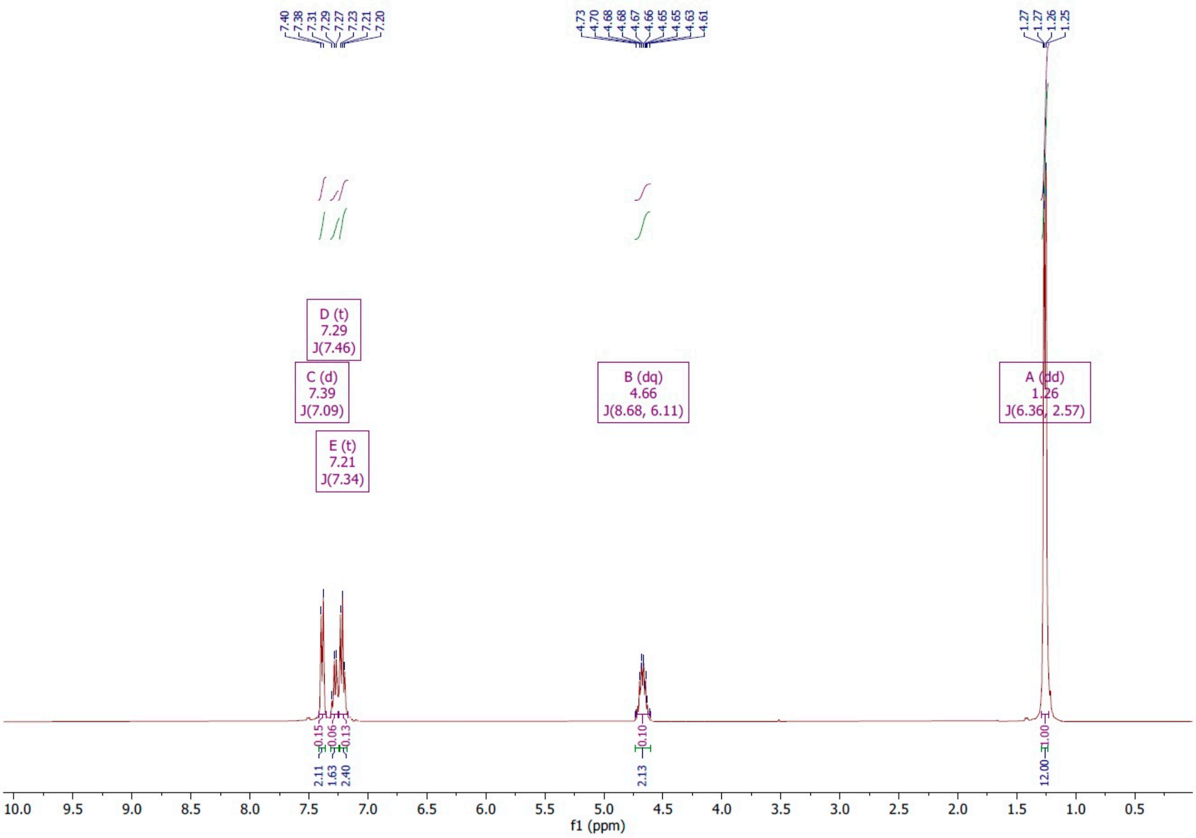

Figure S9. ^{1}H NMR spectra of diisopropyl(phenylethynyl)phosphonate (**4c**).

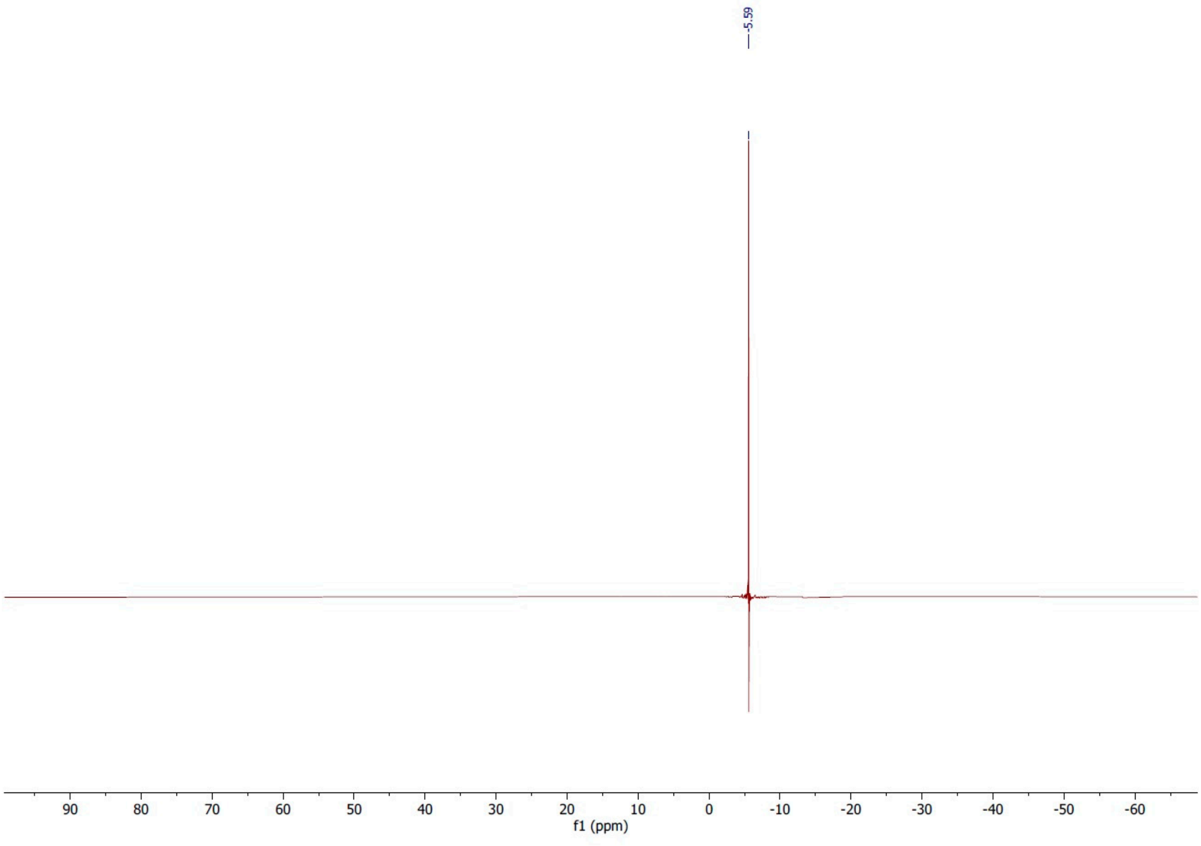

Figure S10. ^{31}P NMR spectra of dibutyl(phenylethynyl)phosphonate (**4d**).

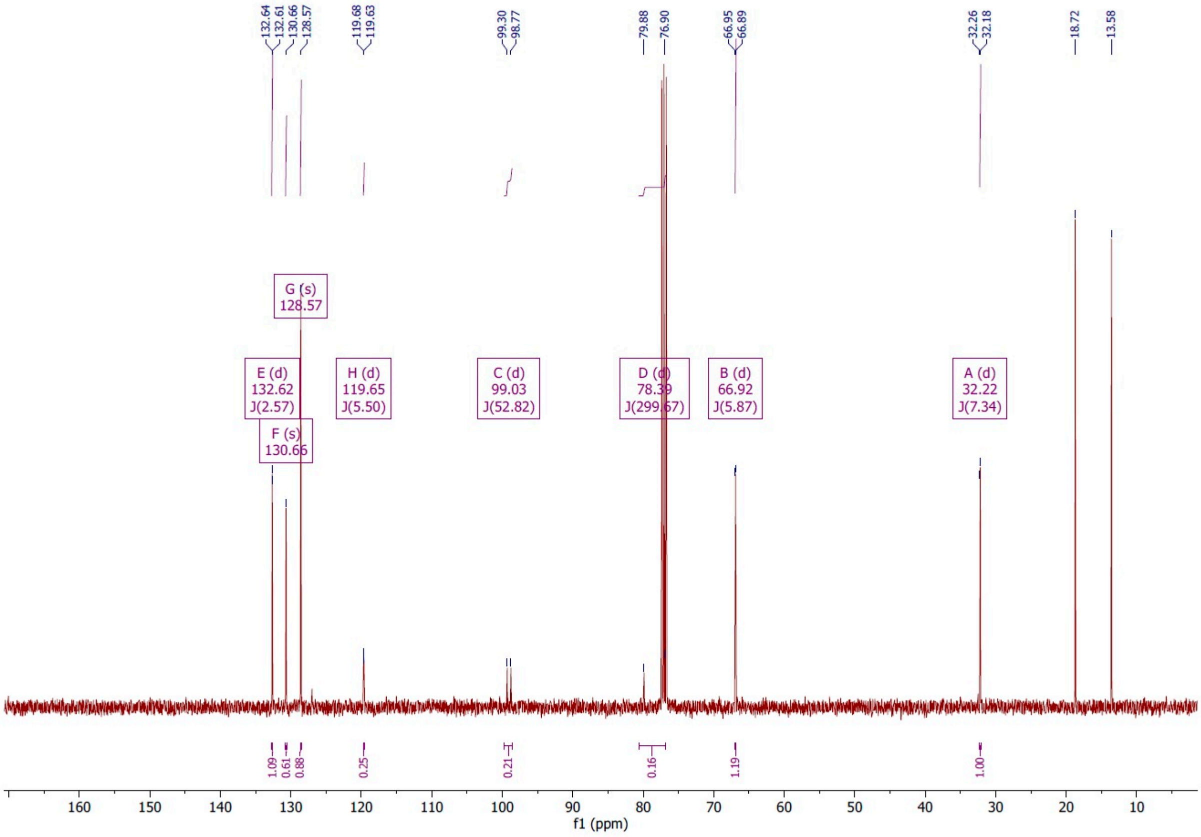

Figure S11. ^{13}C NMR spectra of dibutyl(phenylethynyl)phosphonate (**4d**).

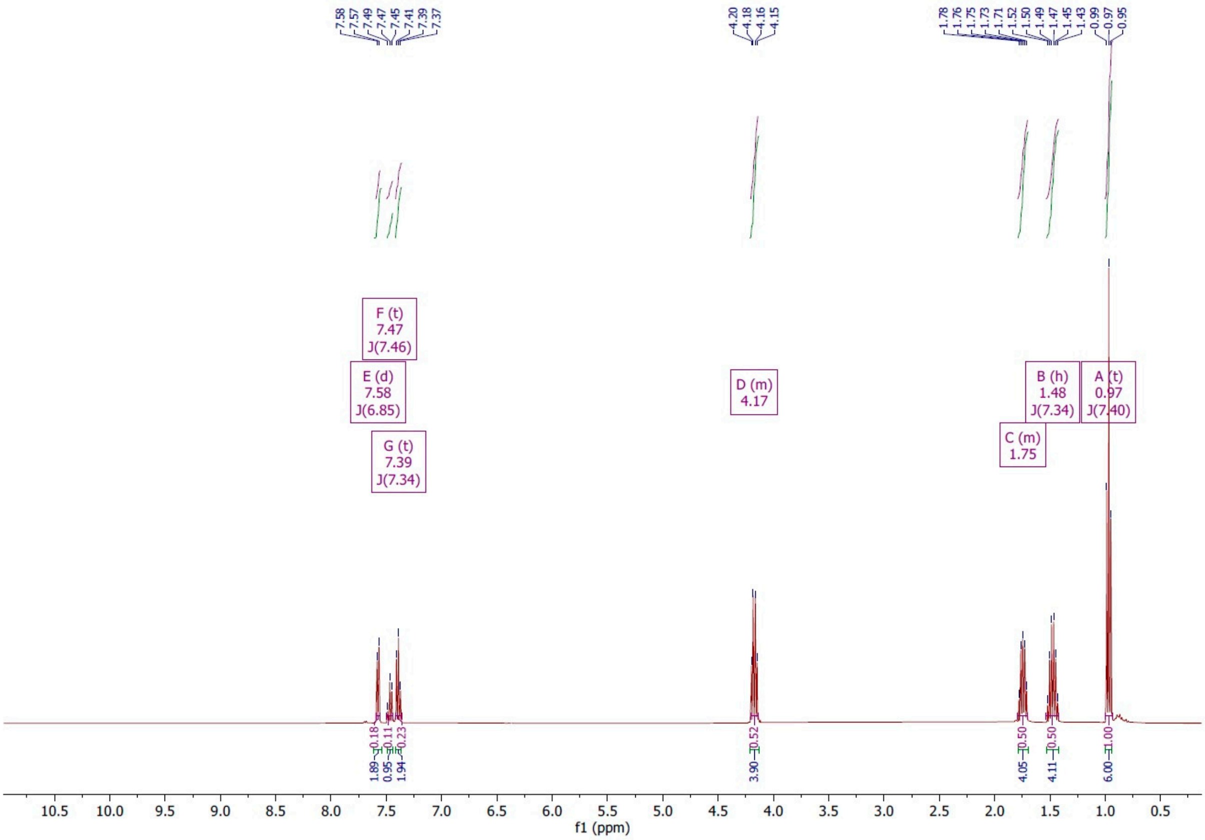

Figure S12. ^{1}H NMR spectra of dibutyl(phenylethynyl)phosphonate (**4d**).

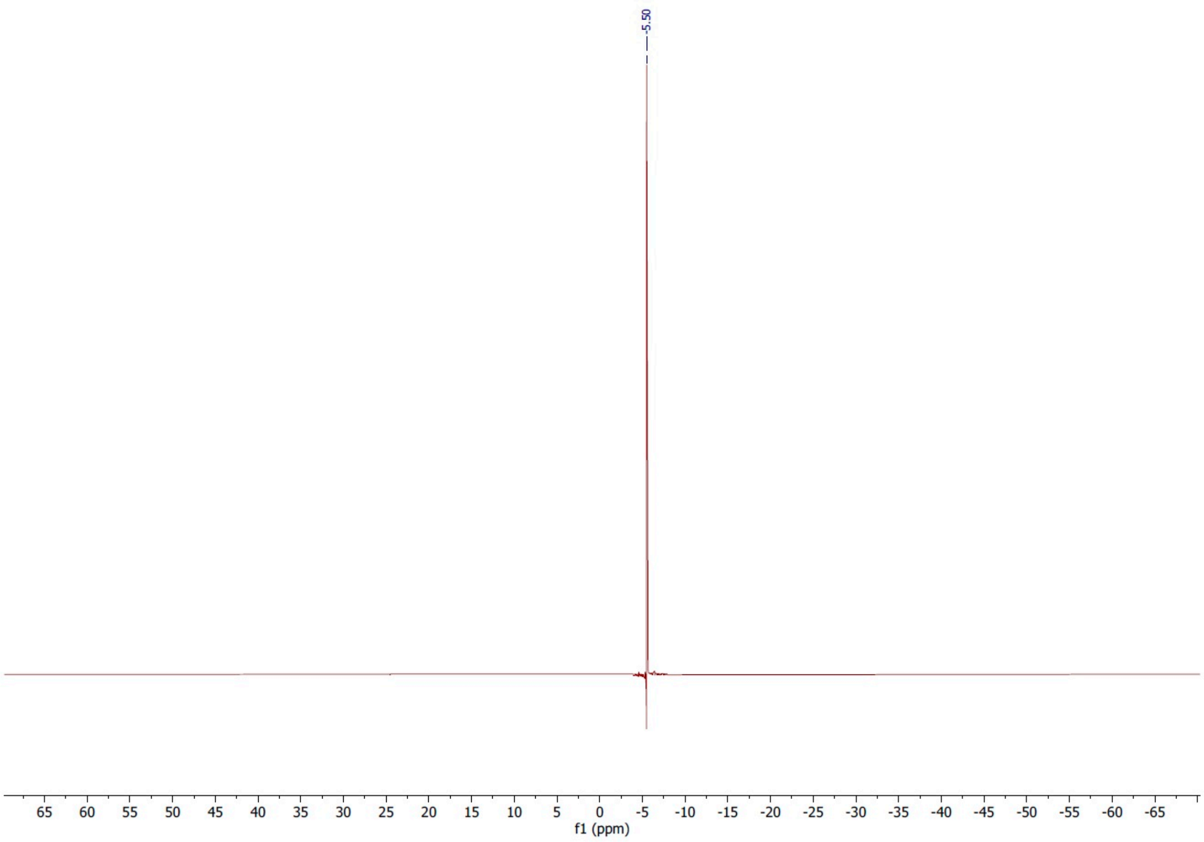

Figure S13. ^{31}P NMR spectra of diisobutyl(phenylethynyl)phosphonate (**4e**).

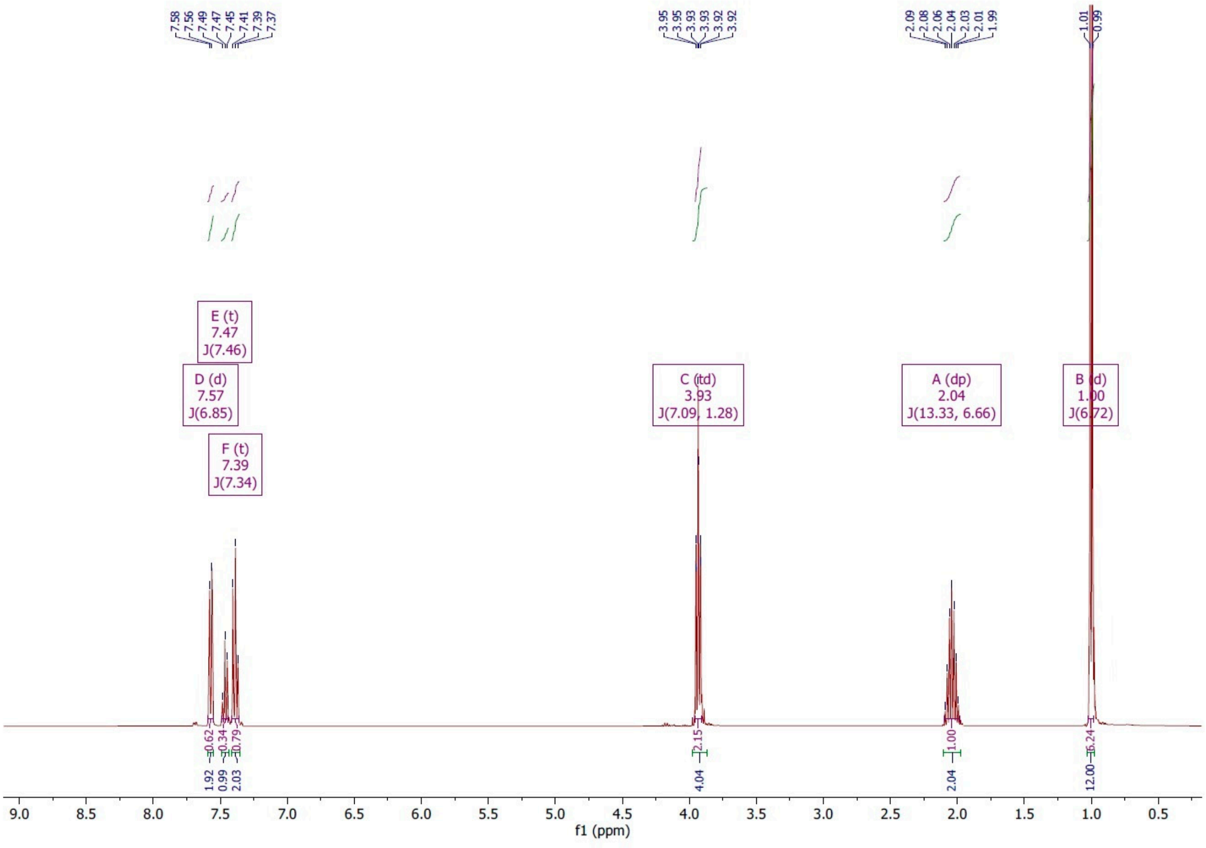

Figure S14. ^{1}H NMR spectra of diisobutyl(phenylethynyl)phosphonate (**4e**).

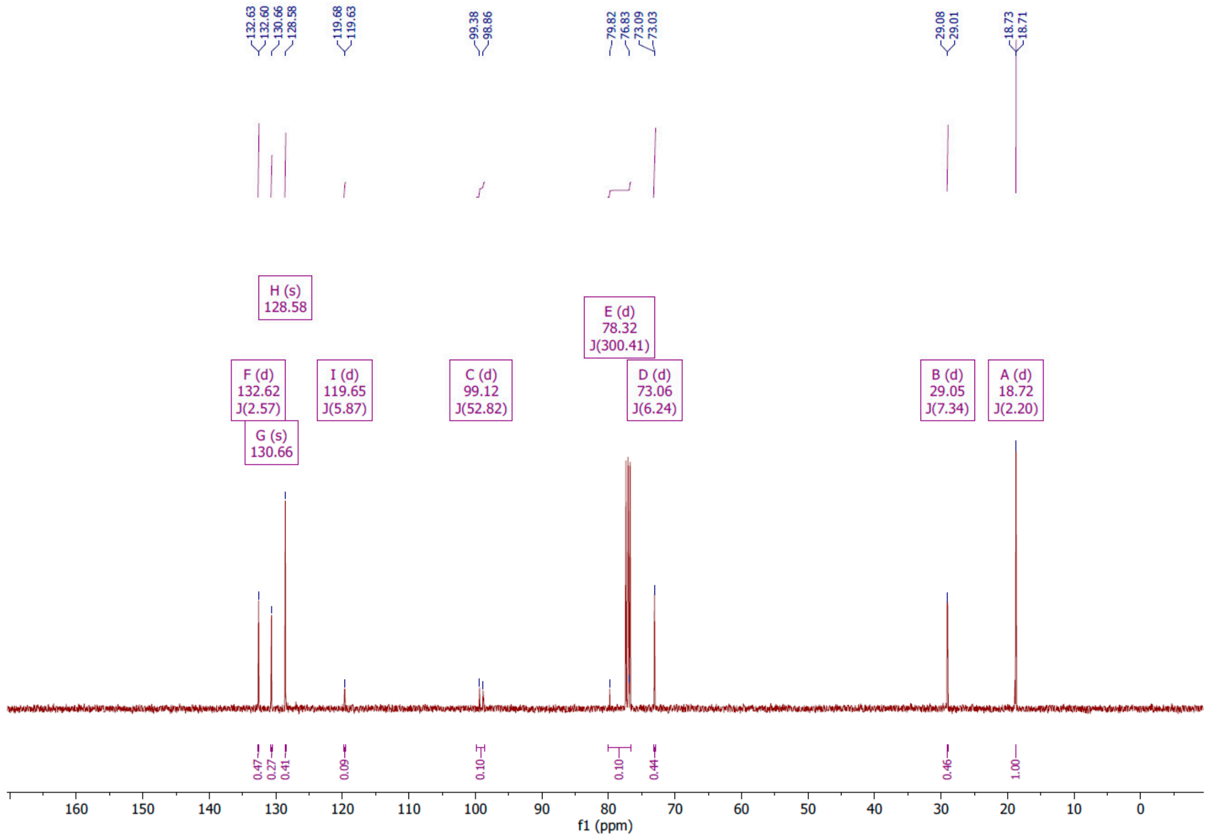

Figure S15. ^{13}C NMR spectra of diisobutyl(phenylethynyl)phosphonate (**4e**).

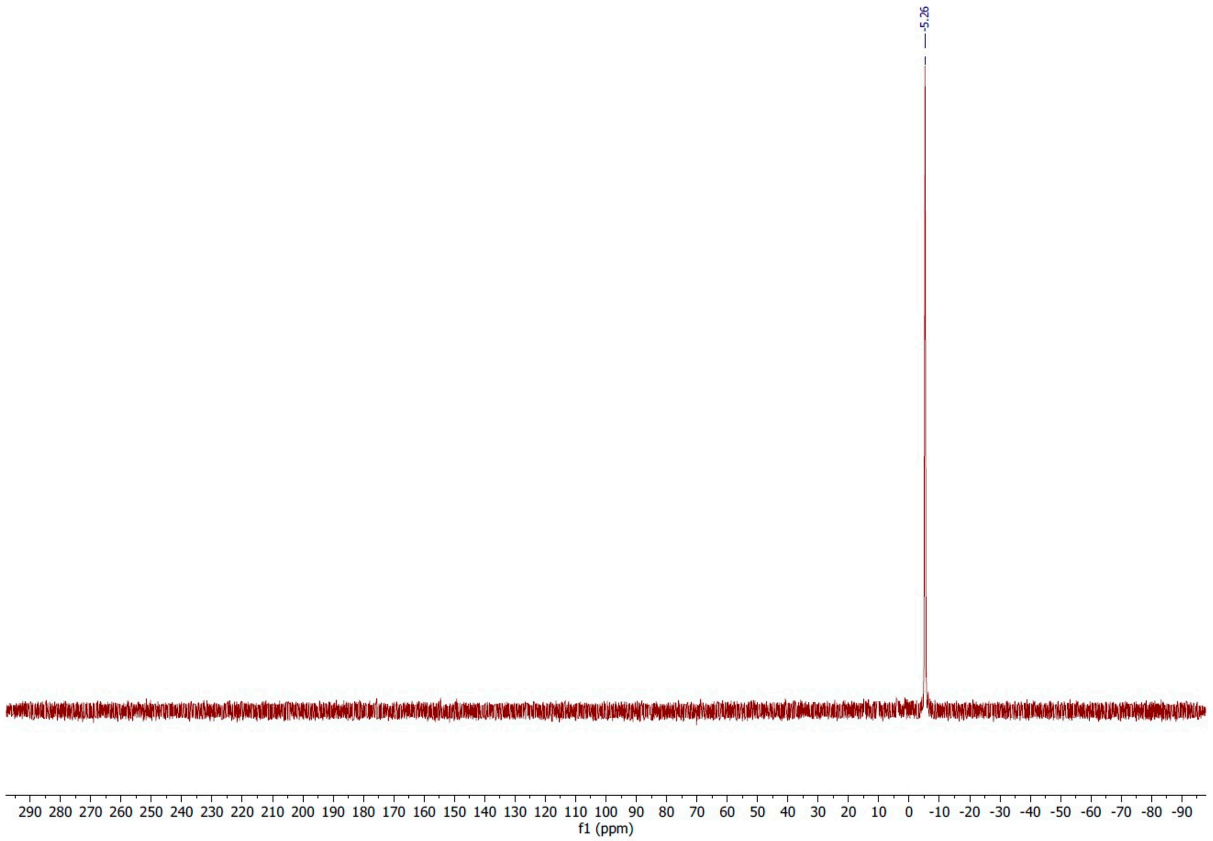

Figure S16. ^{13}C NMR spectra of ditret-butyl(phenylethynyl)phosphonate (**4f**).

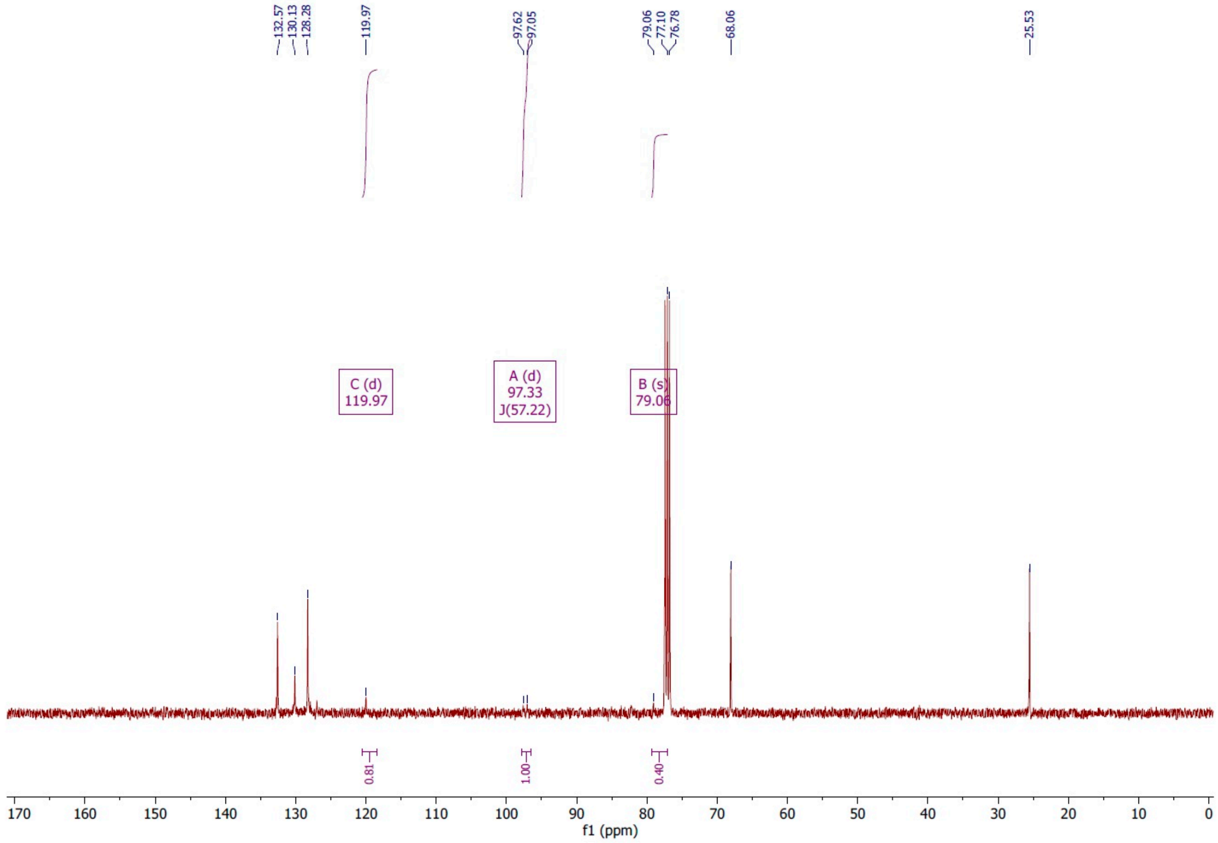

Figure S17. ^{13}C NMR spectra of ditret-butyl(phenylethynyl)phosphonate (**4f**).

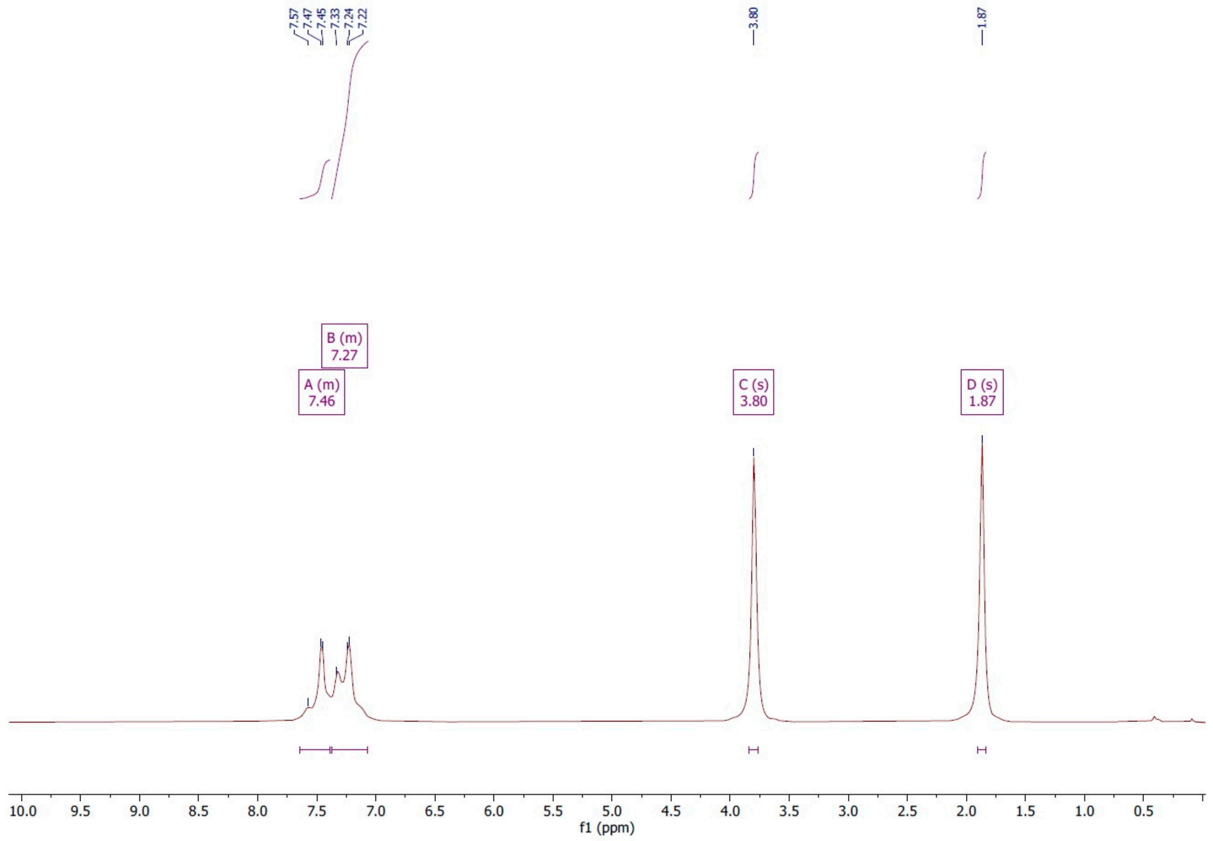

Figure S18. ^{13}C NMR spectra of ditret-butyl(phenylethynyl)phosphonate (**4f**).

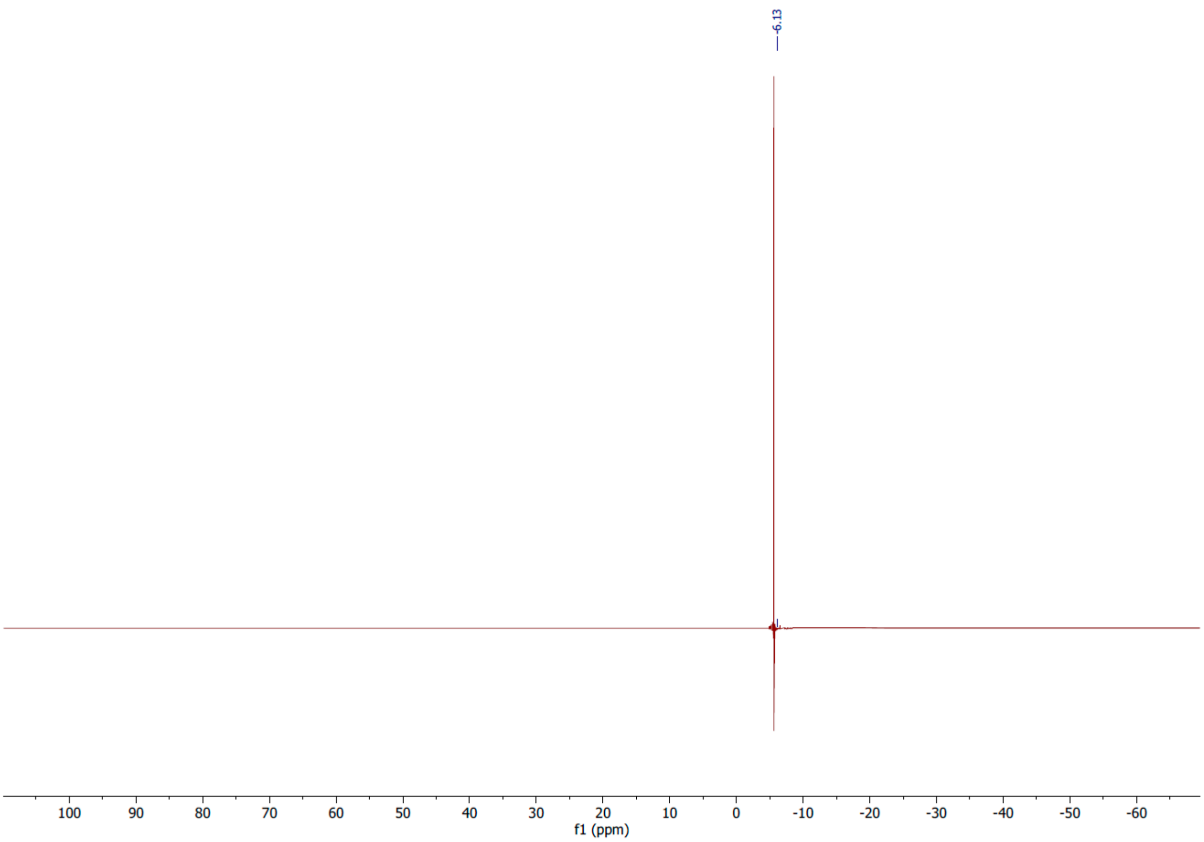

Figure S19. ^{31}P NMR spectra of dihexyl(phenylethynyl)phosphonate (**4g**).

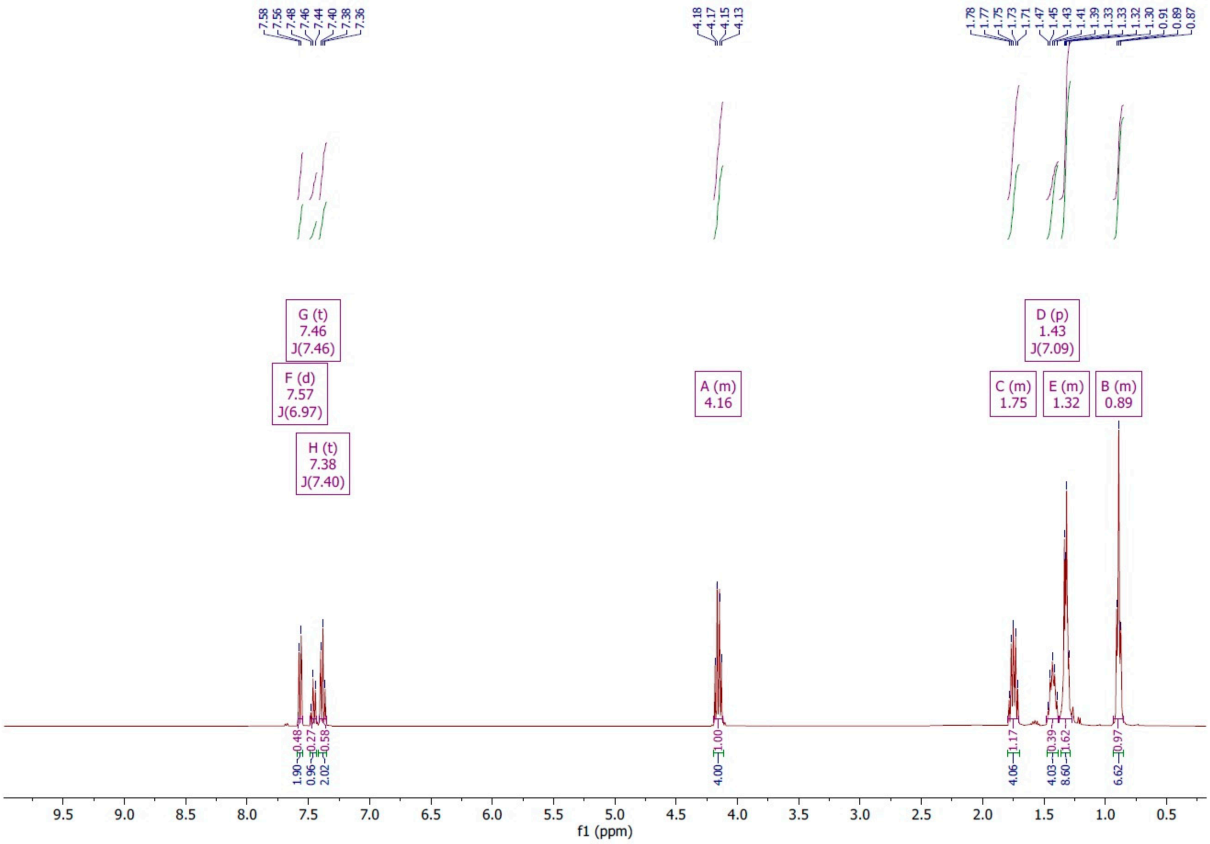

Figure S20. ^{1}H NMR spectra of dihexyl(phenylethynyl)phosphonate (**4g**).

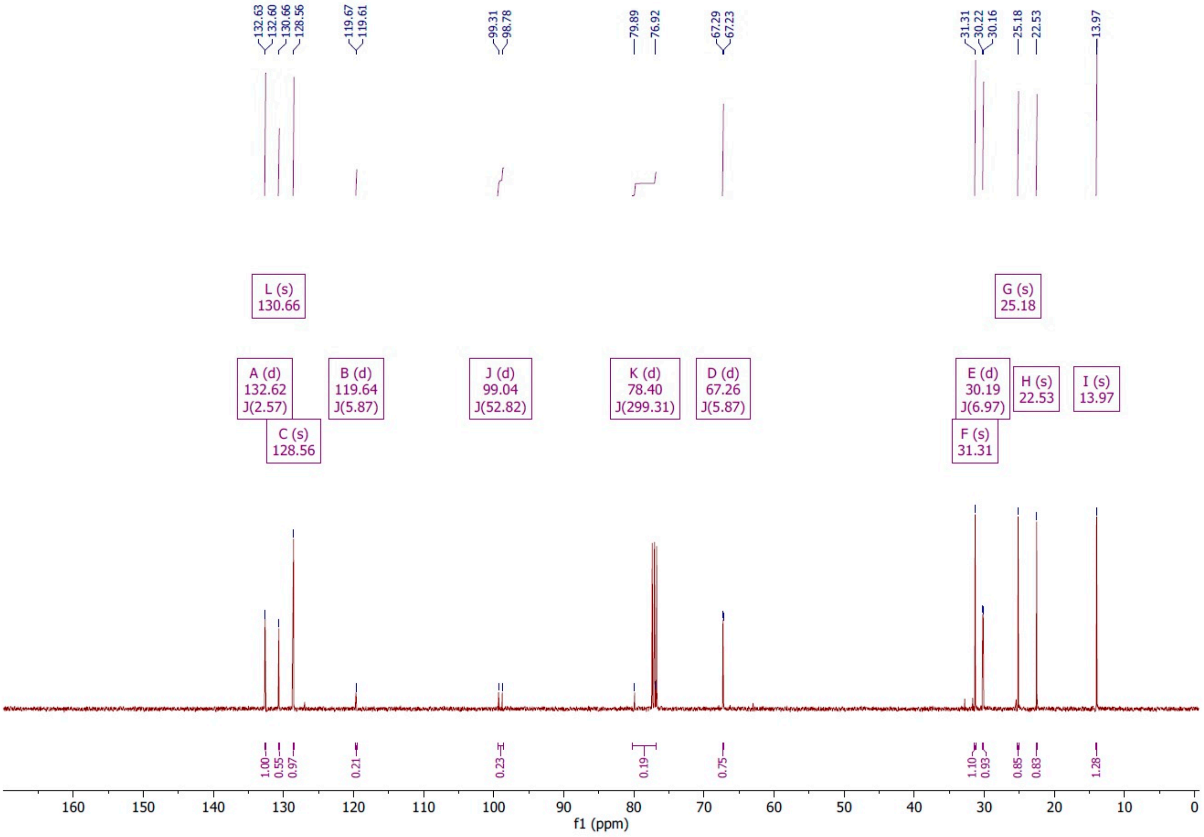

Figure S21. ^{13}C NMR spectra of dihexyl(phenylethynyl)phosphonate (**4g**).

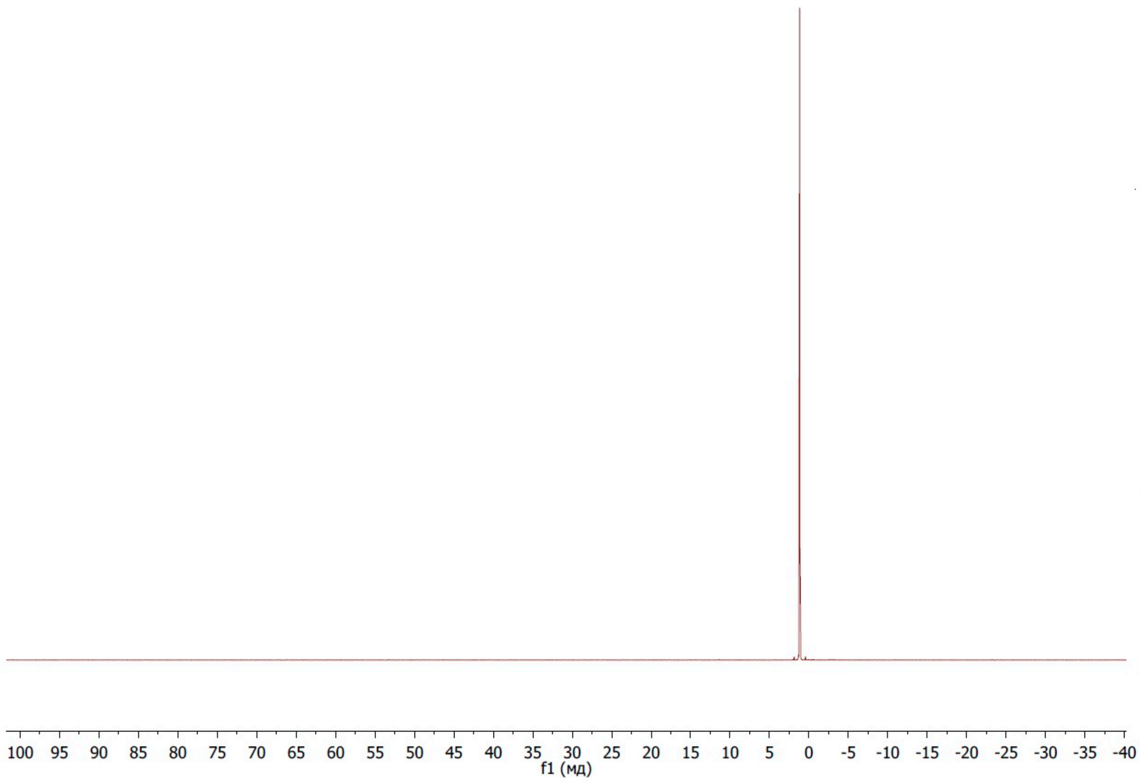

Figure S22. ^{31}P NMR spectra of 1,1'-[(phenylethynyl)phosphoryl]dipyrrolidine (**6a**).

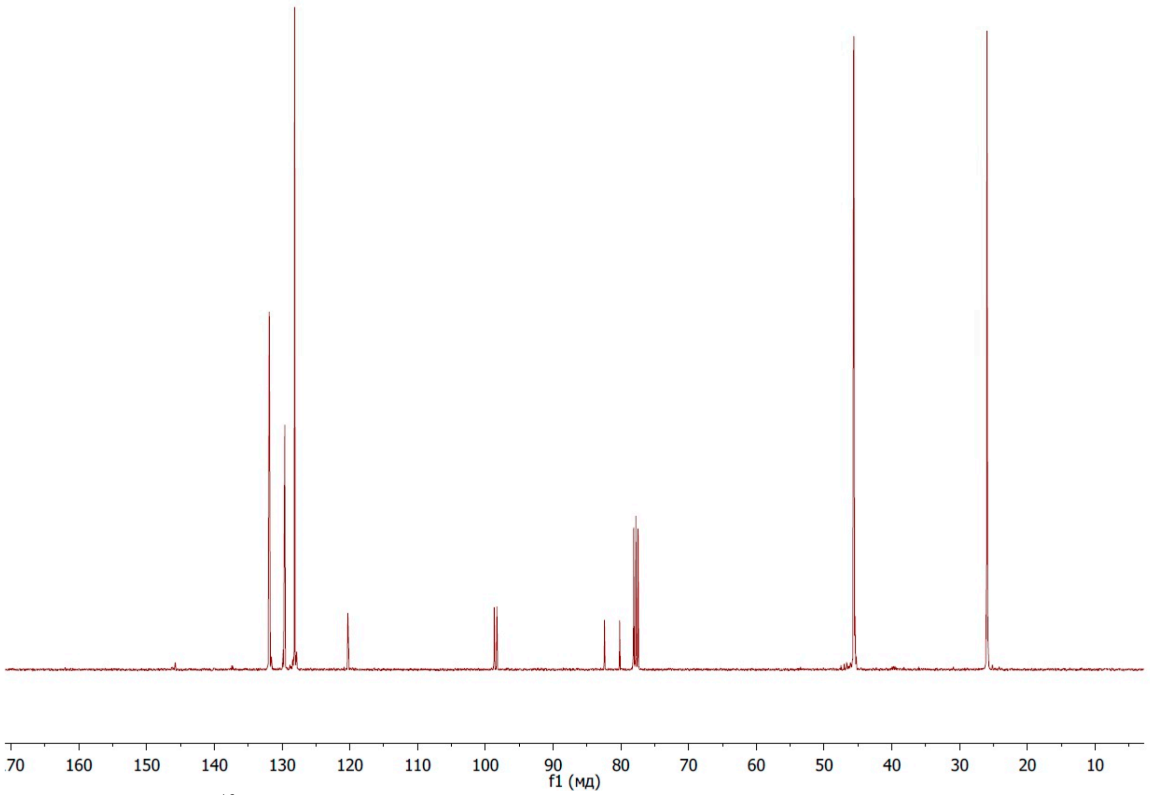

Figure S23. ^{13}C NMR spectra of 1,1'-[(phenylethynyl)phosphoryl]dipyrrolidine (**6a**).

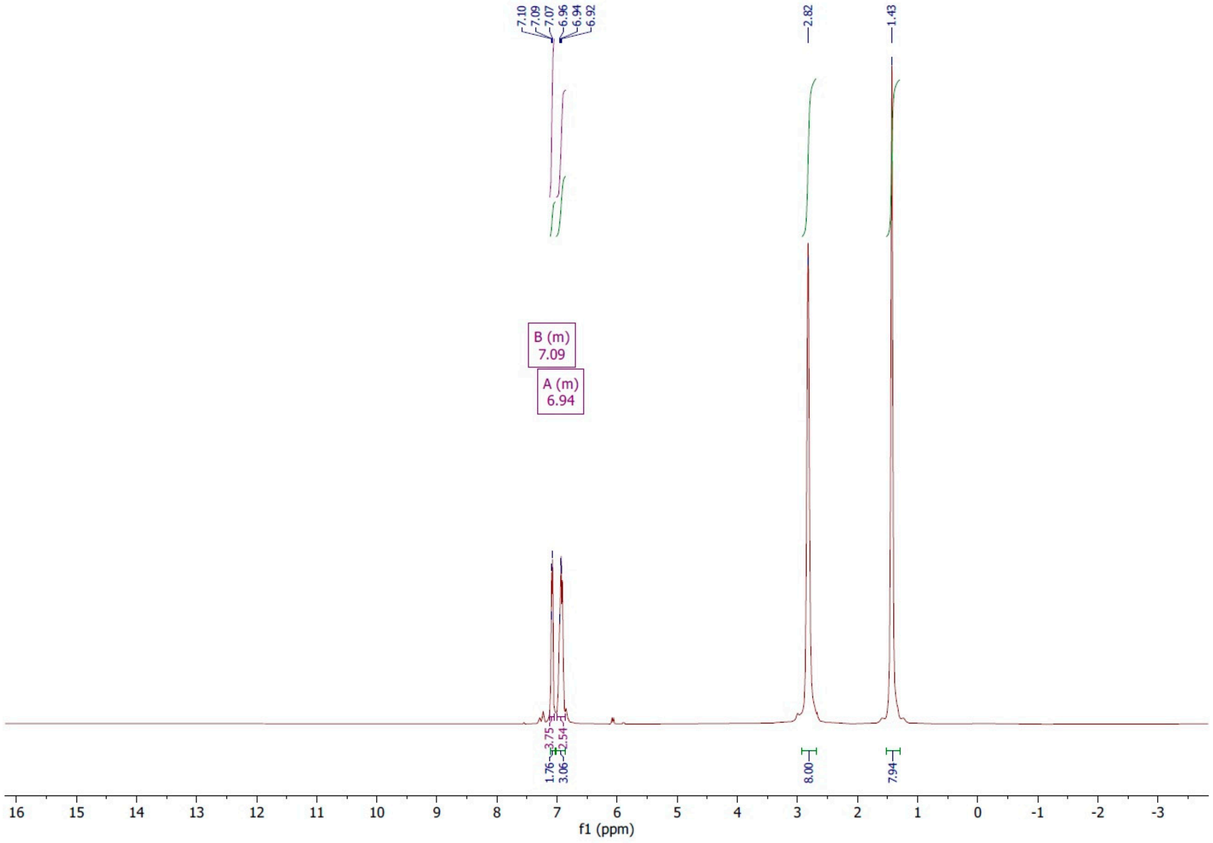

Figure S24. ^{1}H NMR spectra of 1,1'-[(phenylethynyl)phosphoryl]dipyrrolidine (**6a**).

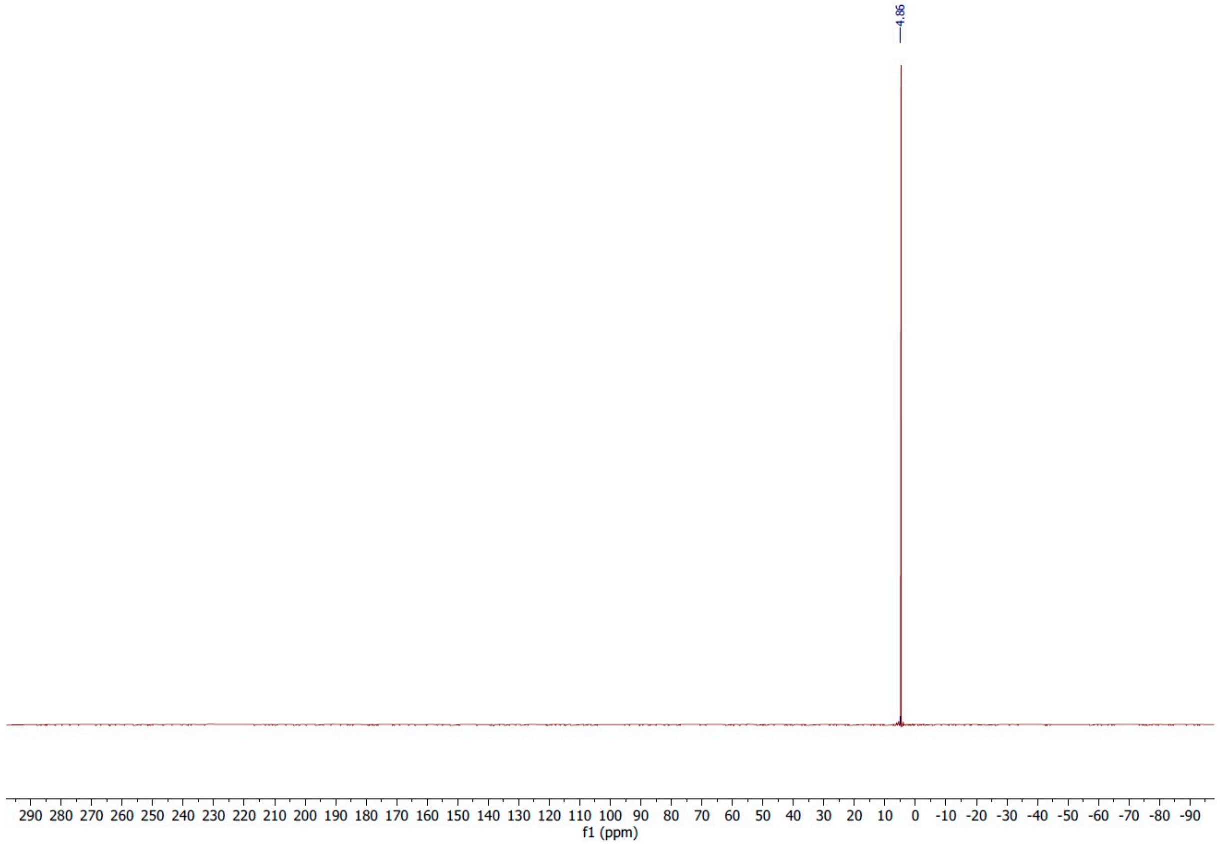

Figure S25. ^{31}P NMR spectra of 4,4'-[(phenylethynyl)phosphoryl]bis(morpholine) (**6b**).

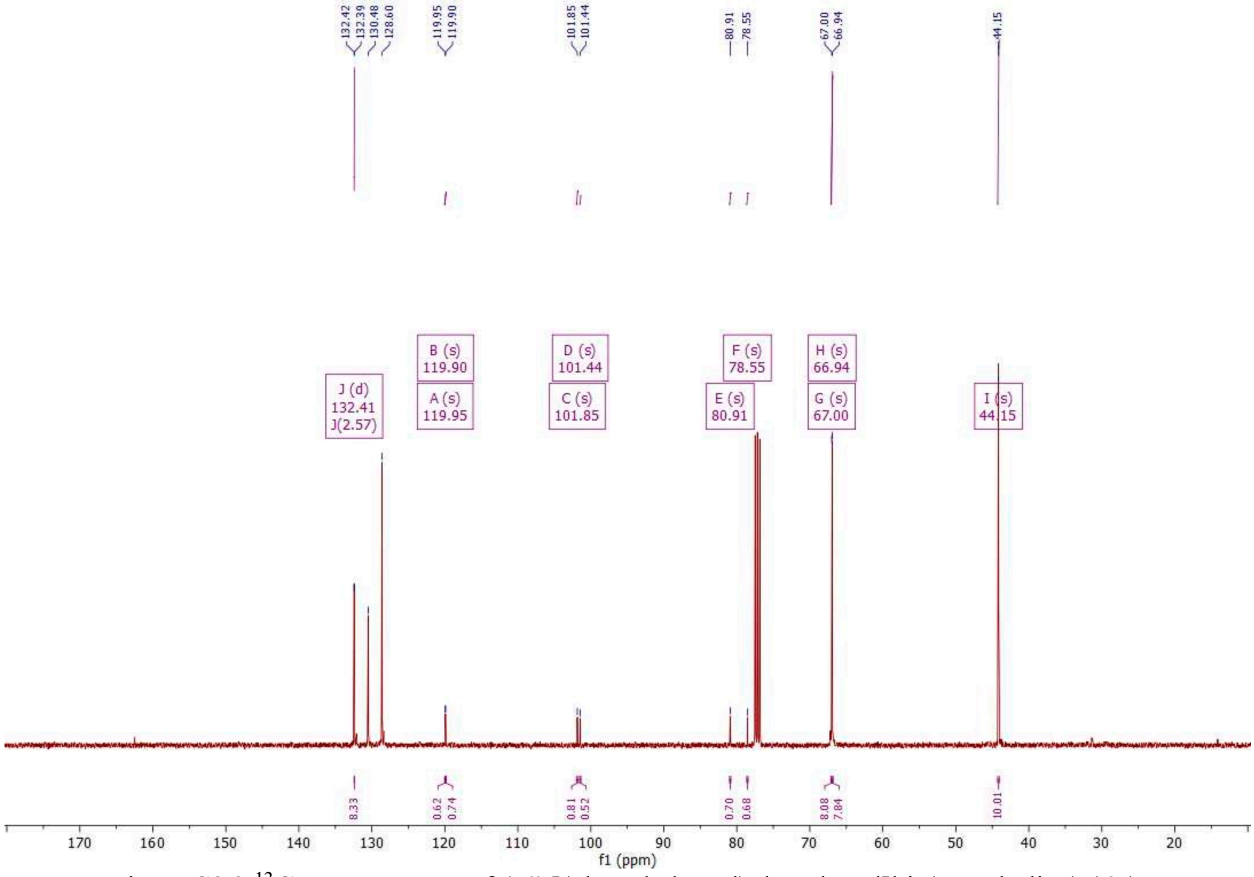

Figure S26. ^{13}C NMR spectra of 4,4'-[(phenylethynyl)phosphoryl]bis(morpholine) (**6b**).

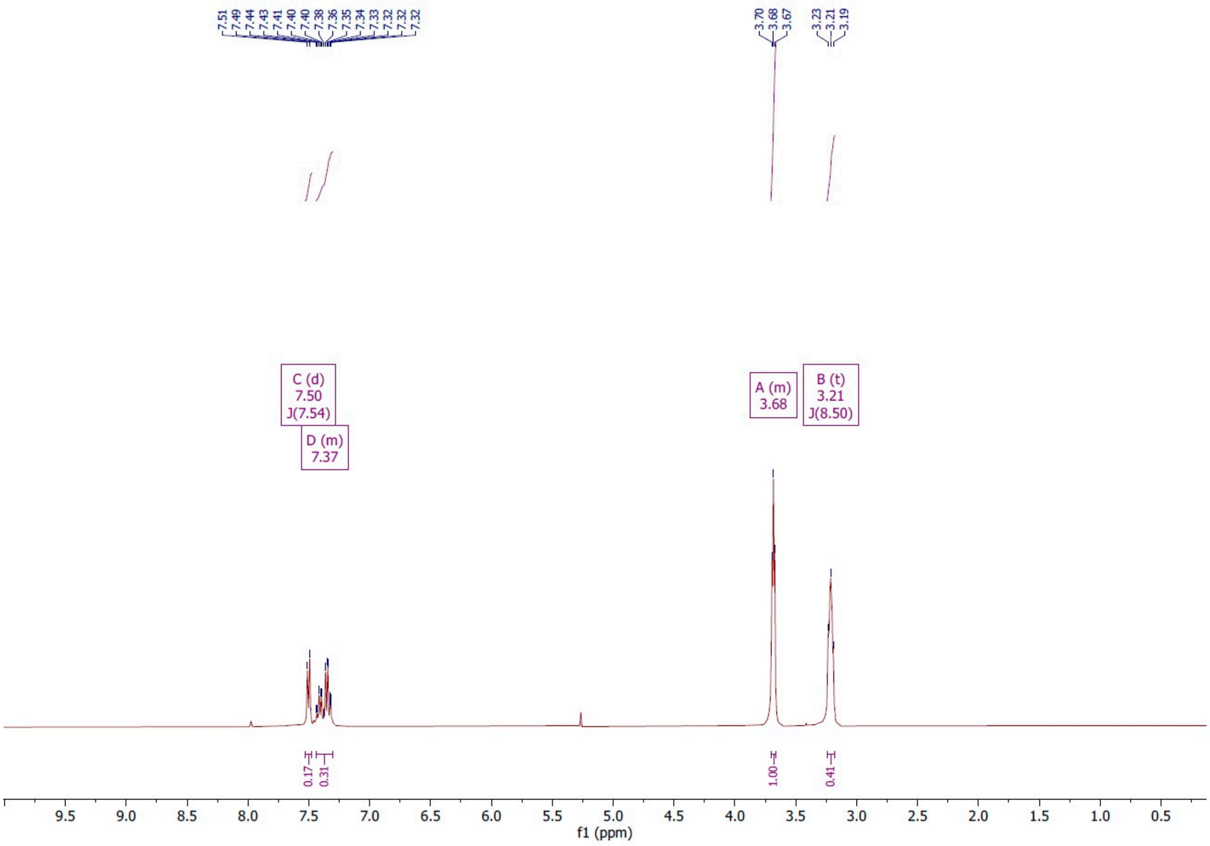

Figure S27. ^{1}H NMR spectra of 4,4'-[(phenylethynyl)phosphoryl]bis(morpholine) (**6b**).

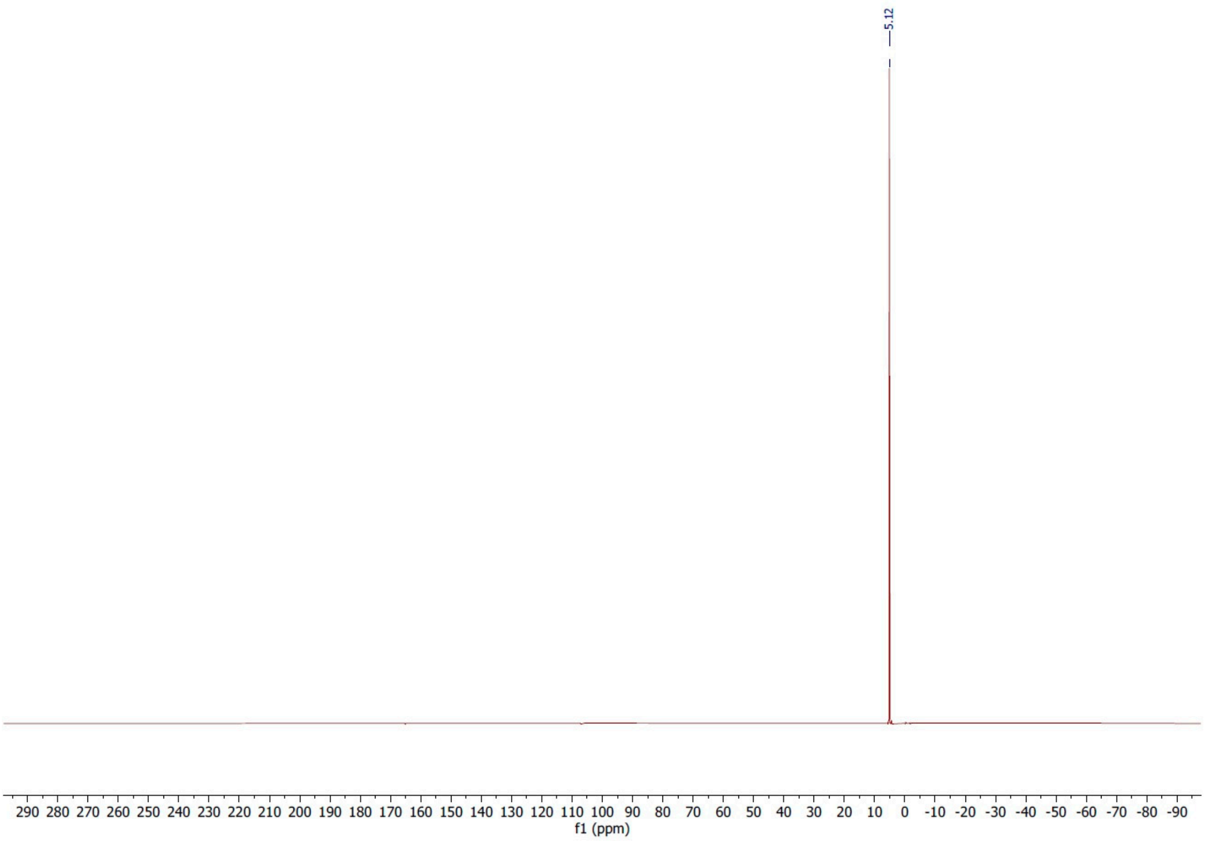

Figure S28. ^{31}P NMR spectra of 1,1'-[(phenylethynyl)phosphoryl]dimethylpiperazine (**6c**).

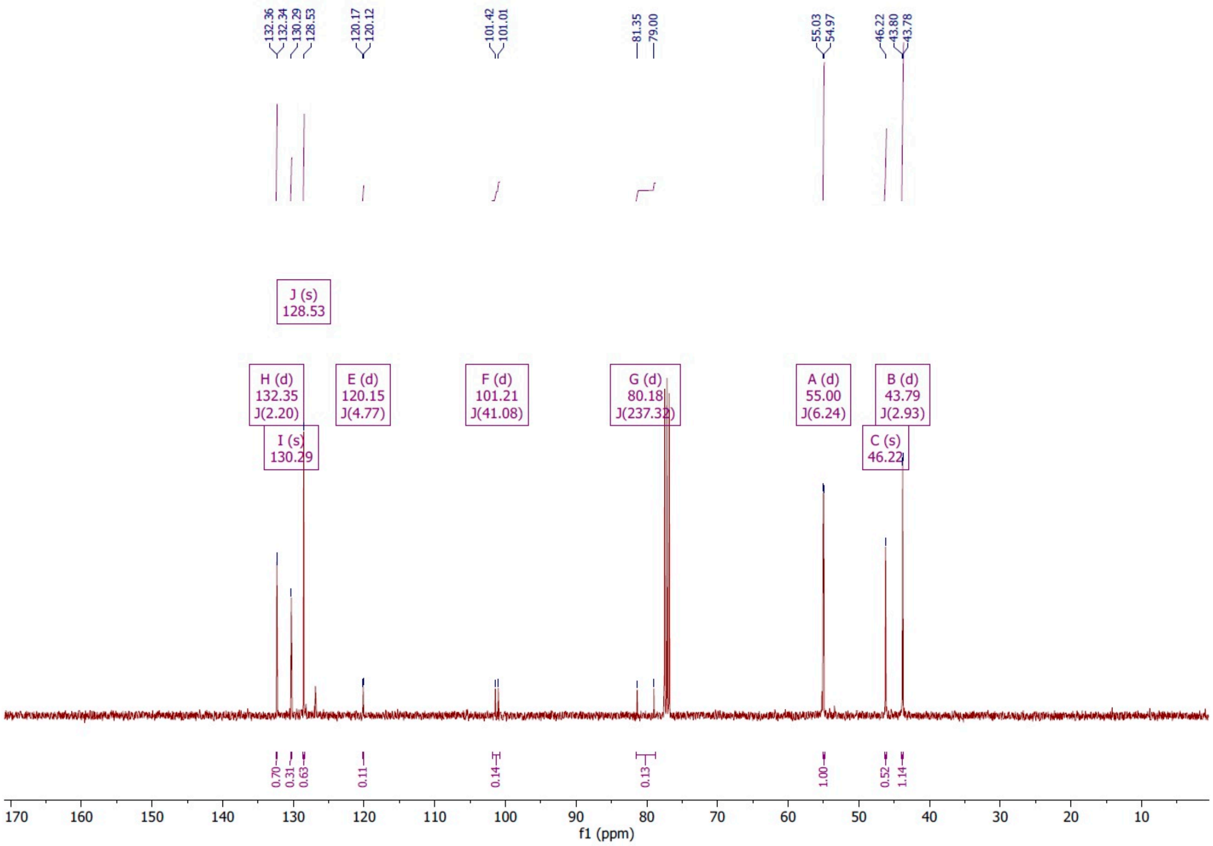

Figure S29. ^{13}C NMR spectra of 1,1'-[(phenylethynyl)phosphoryl]dimethylpiperazine (**6c**).

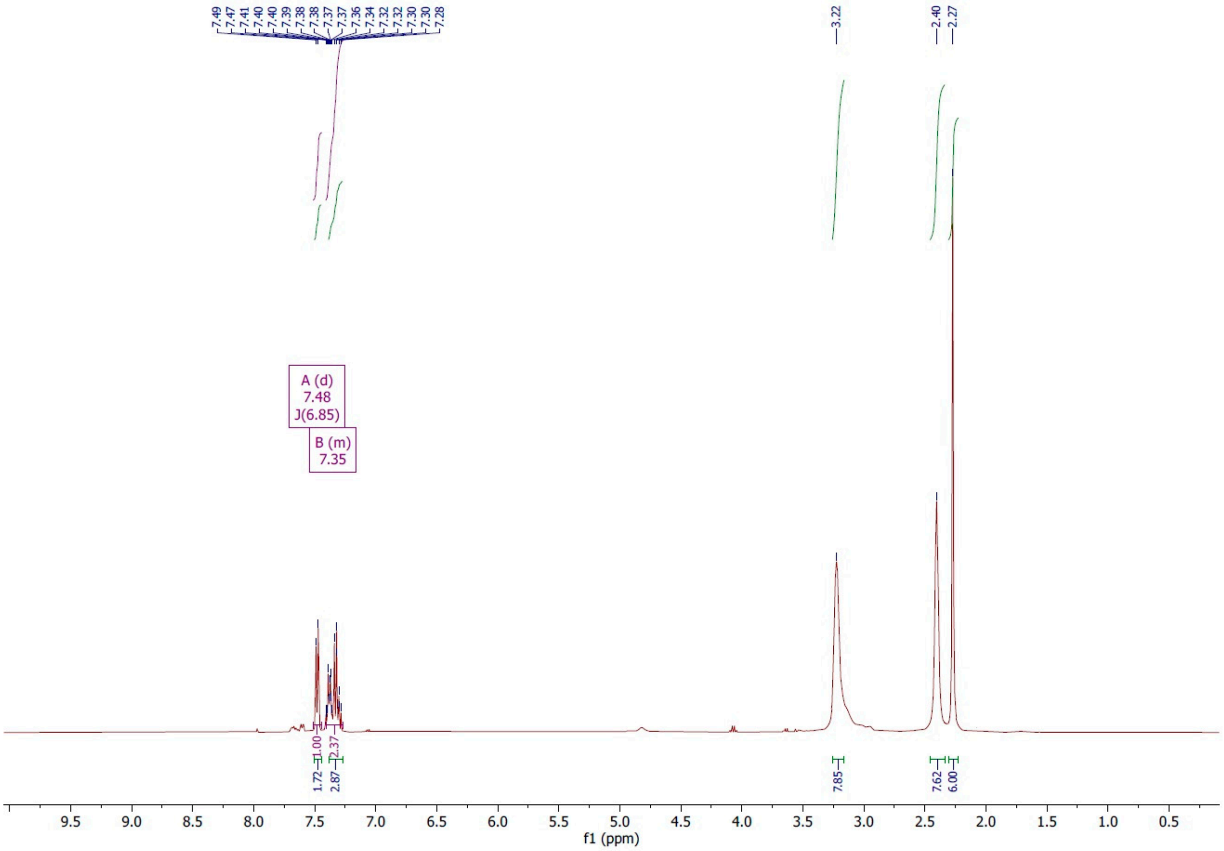

Figure S30. ^{1}H NMR spectra of 1,1'-[(phenylethynyl)phosphoryl]dimethylpiperazine (**6c**).

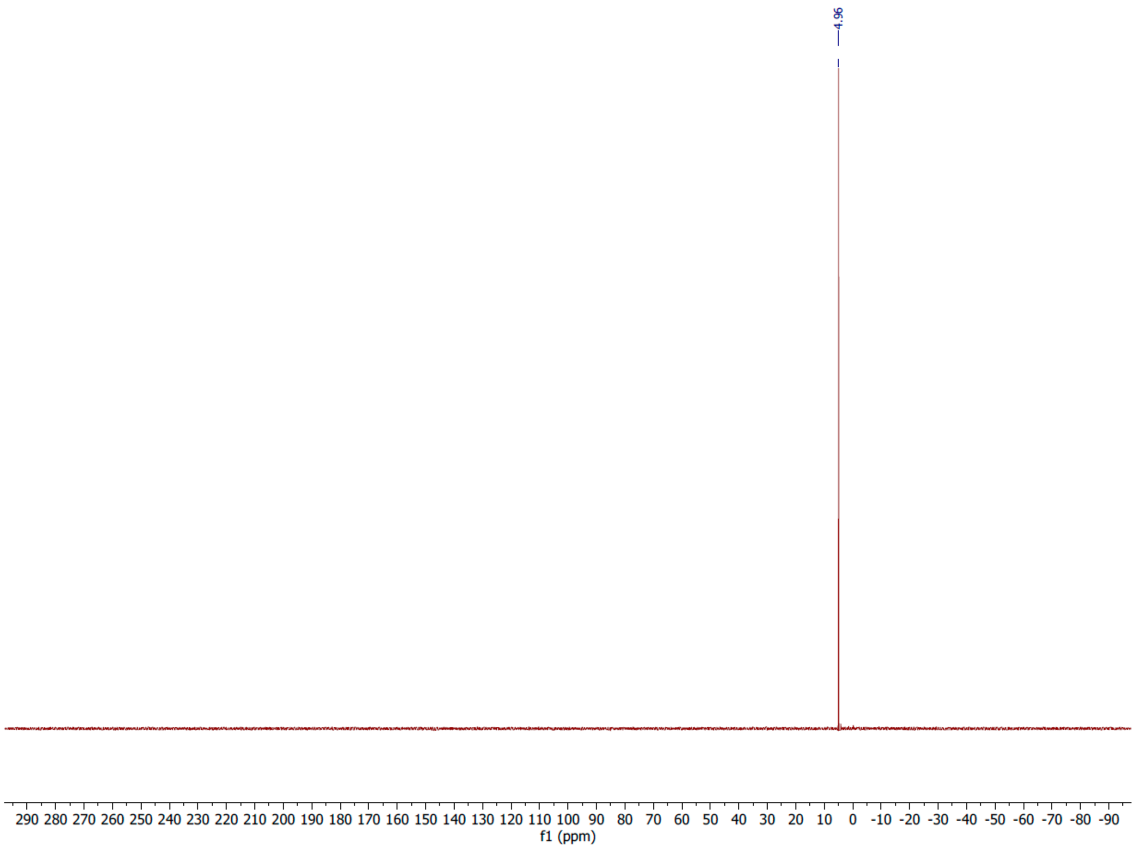

Figure S31. ^{31}P NMR spectra of 1,1'-[(phenylethynyl)phosphoryl]diethylpiperazine (**6d**).

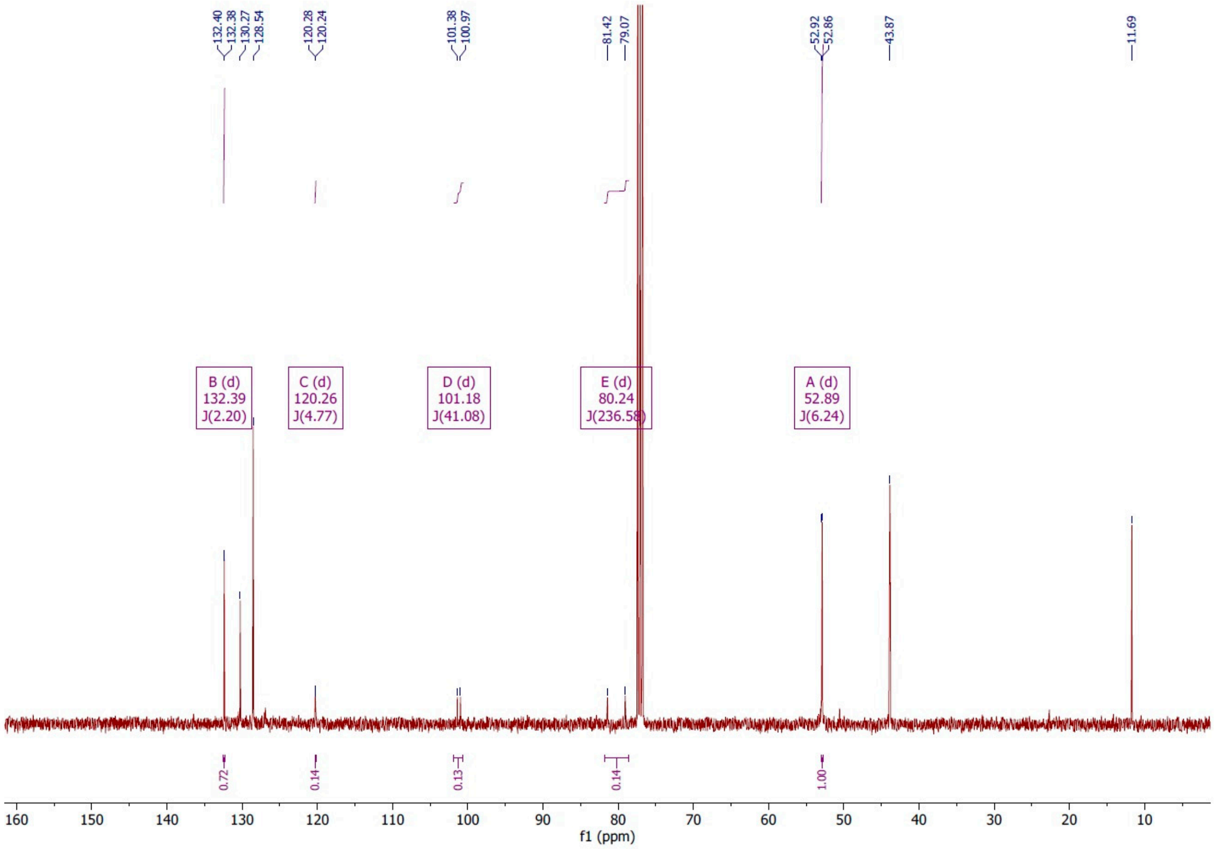

Figure S32. ^{13}C NMR spectra of 1,1'-[(phenylethynyl)phosphoryl]diethylpiperazine (**6d**).

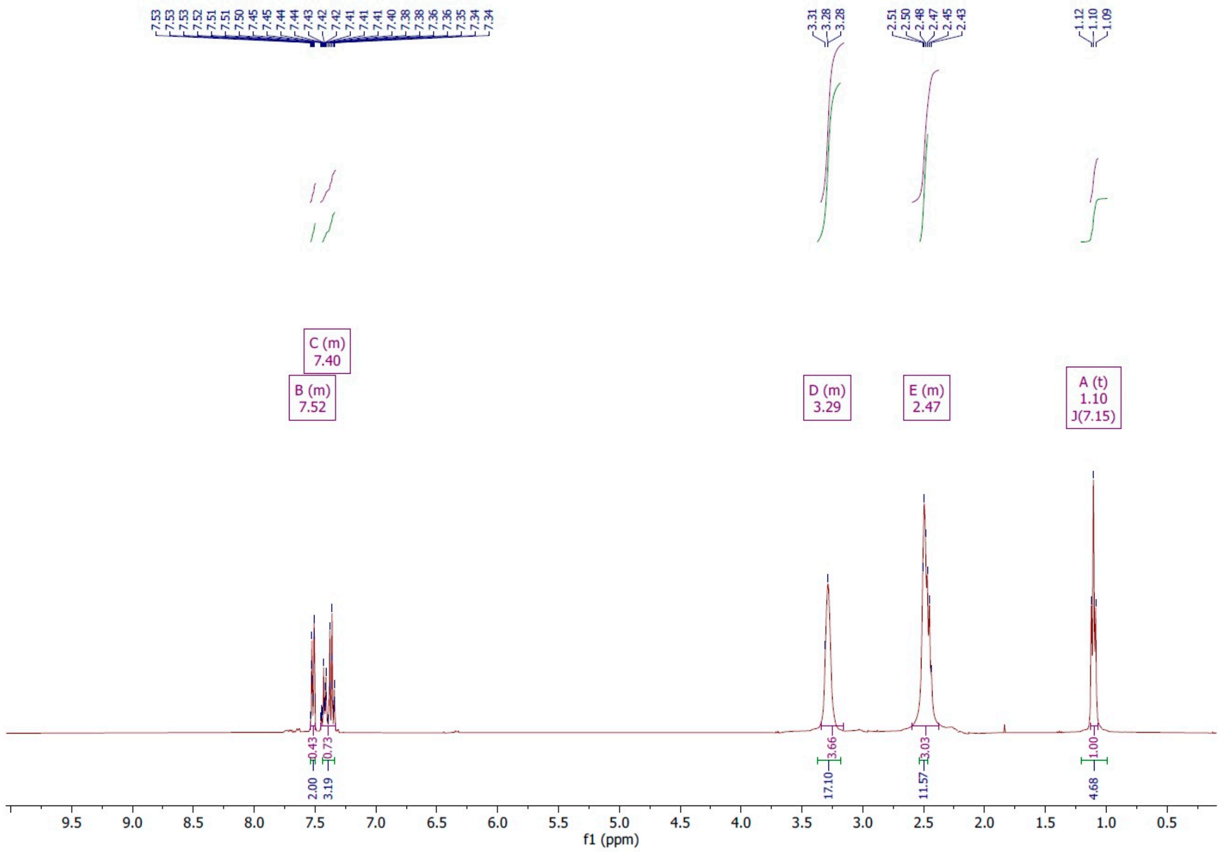

Figure S33. ^{1}H NMR spectra of 1,1'-[(phenylethynyl)phosphoryl]diethylpiperazine (**6d**).

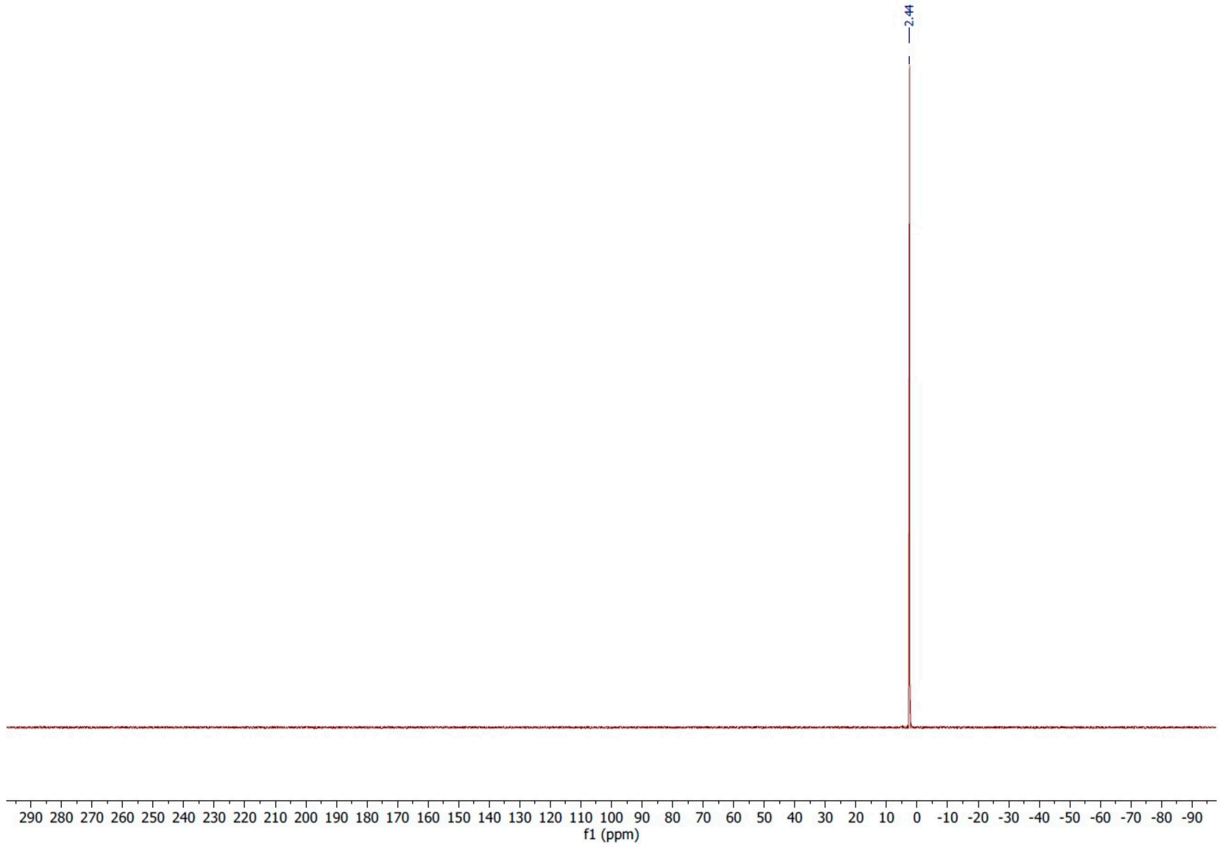

Figure S34. ^{31}P NMR spectra of 4-[(phenylethynyl)(pyrrolidin-1-yl)phosphoryl]morpholine (**6e**).

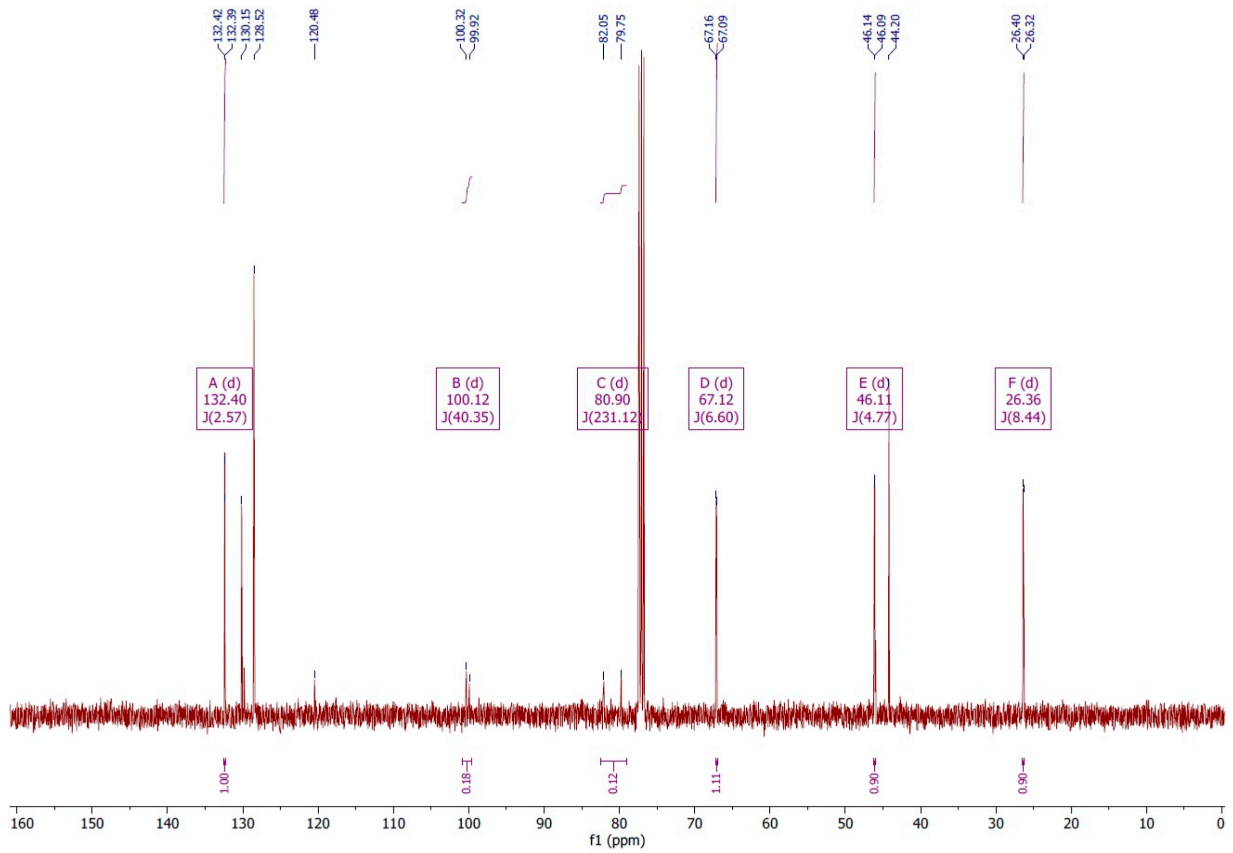

Figure S35. ^{13}C NMR spectra of 4-[(phenylethynyl)(pyrrolidin-1-yl)phosphoryl]morpholine (**6e**).

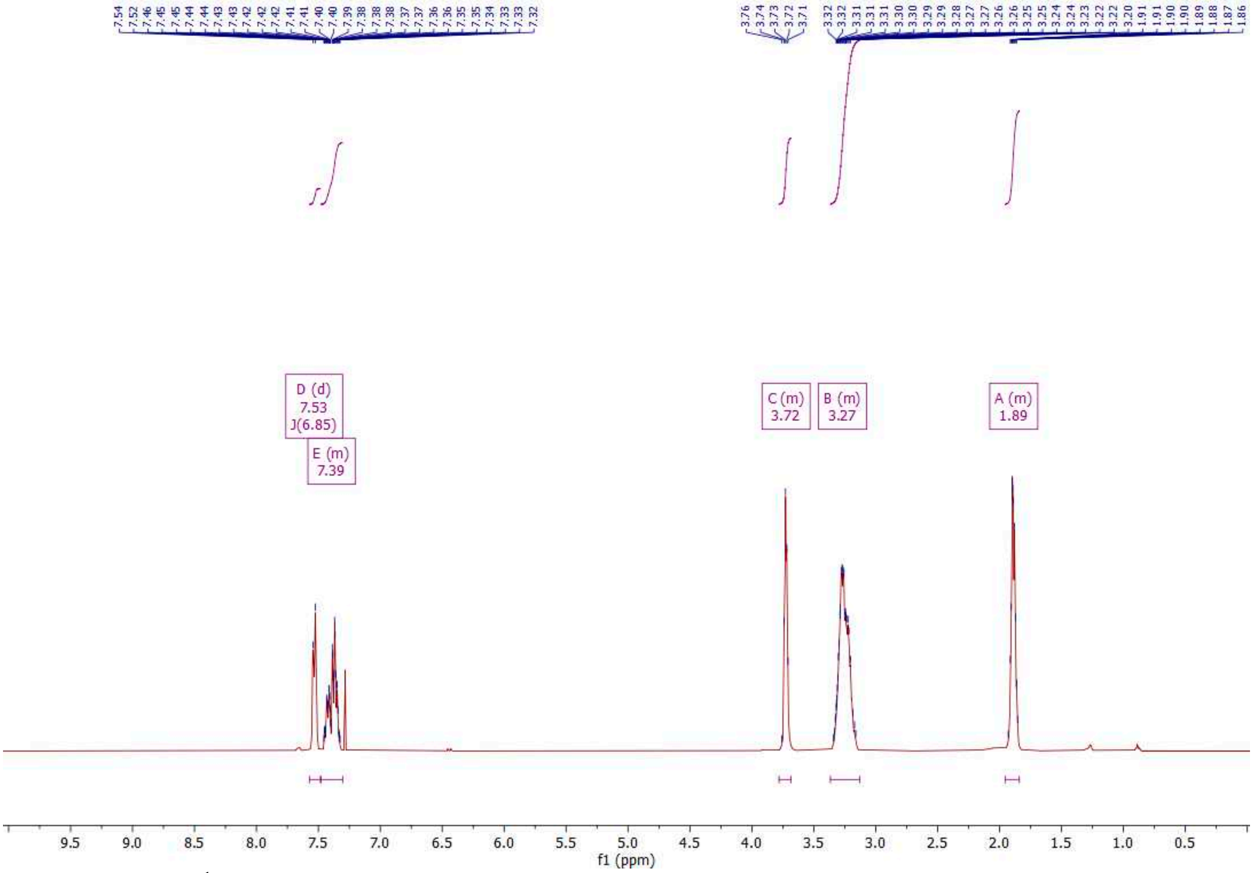

Figure S36. ^{1}H NMR spectra of 4-[(phenylethynyl)(pyrrolidin-1-yl)phosphoryl]morpholine (**6e**).

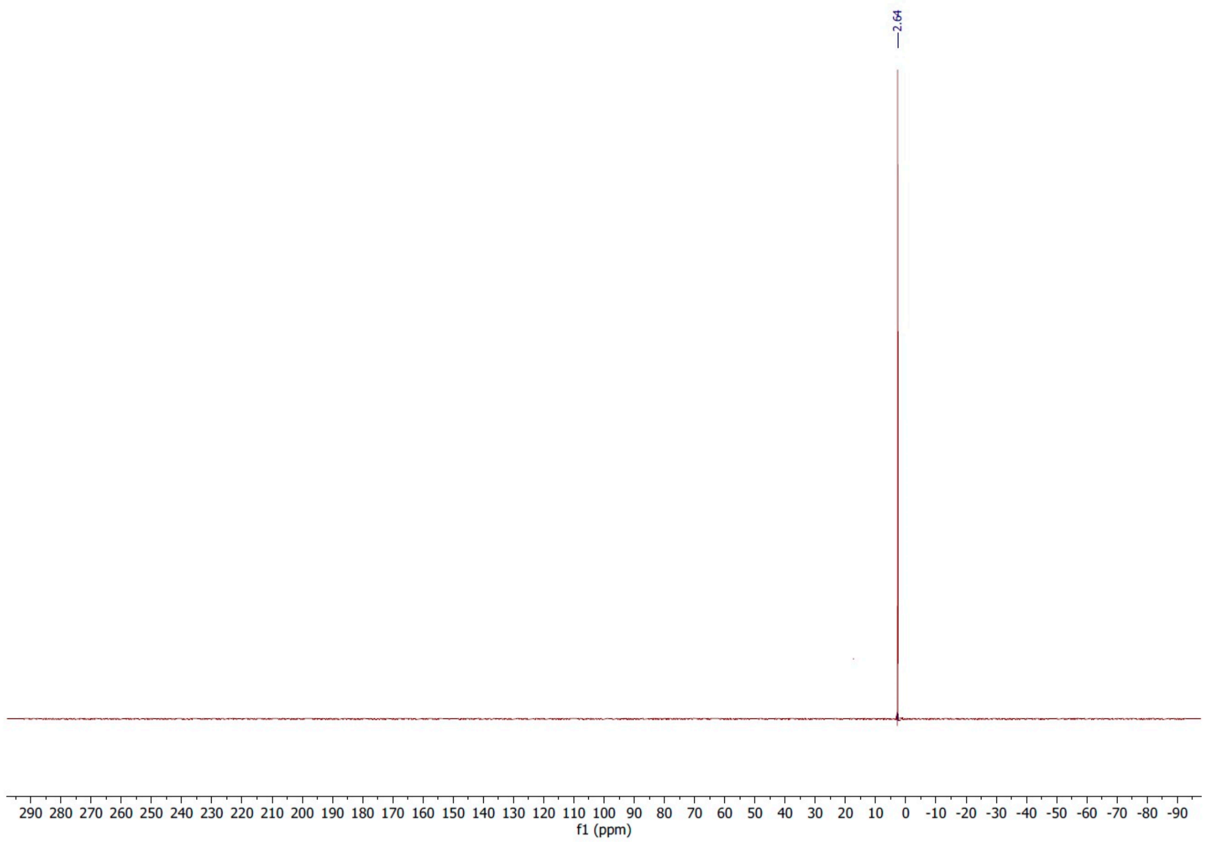

Figure S37. ^{31}P NMR spectra of 4-[(phenylethynyl)(pyrrolidin-1-yl)phosphoryl]1-methylpiperazine (**6f**).

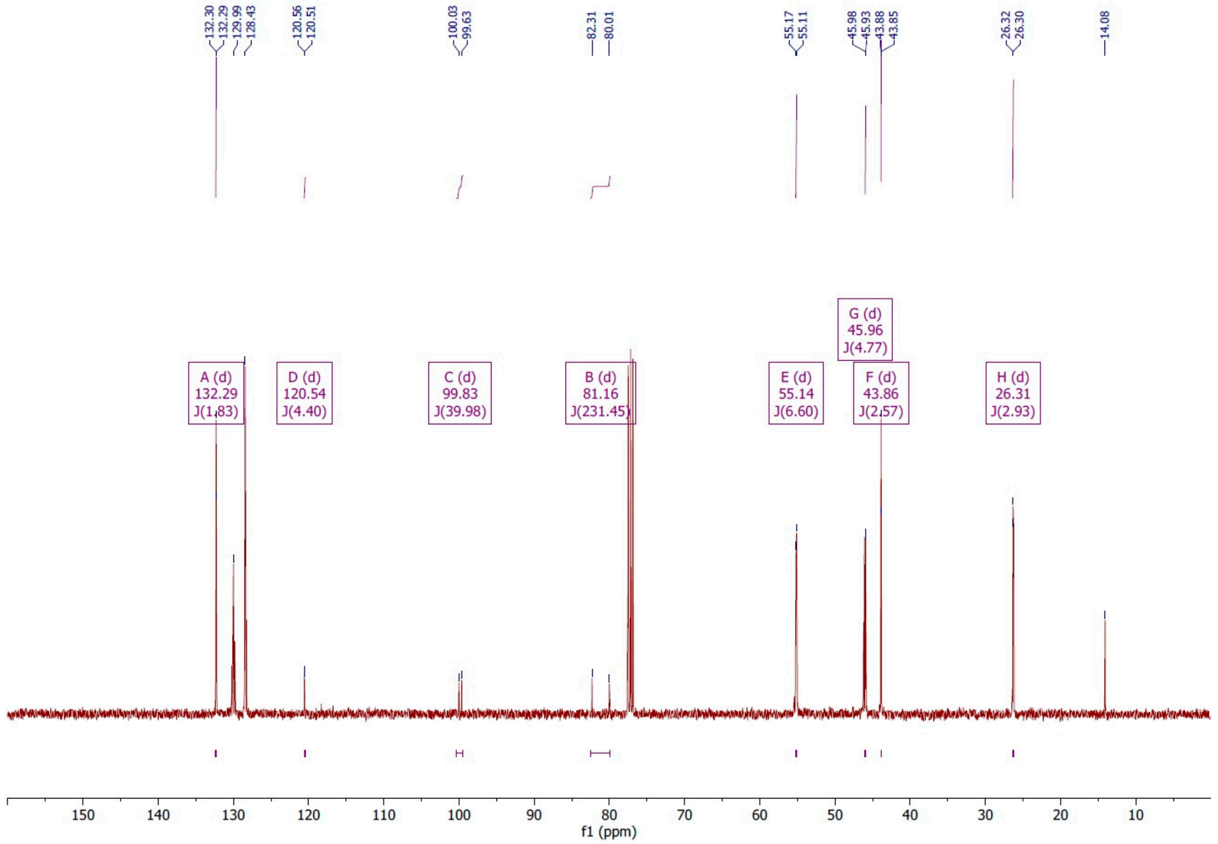

Figure S38. ^{13}C NMR spectra of 4-[(phenylethynyl)(pyrrolidin-1-yl)phosphoryl]1-methylpiperazine (**6f**).

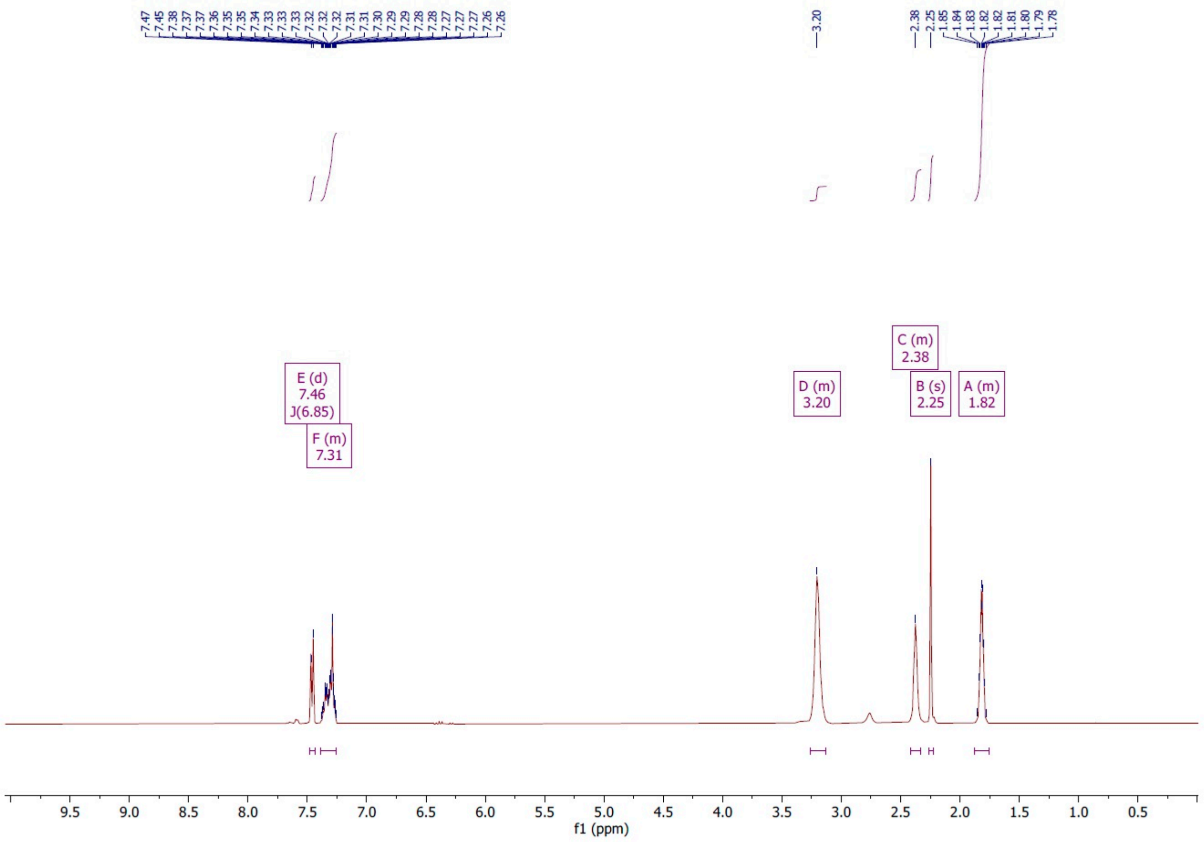

Figure S39. ^{1}H NMR spectra of 4-[(phenylethynyl)(pyrrolidin-1-yl)phosphoryl]1-methylpiperazine (**6f**).

3. HRMS (ESI-TOF) data

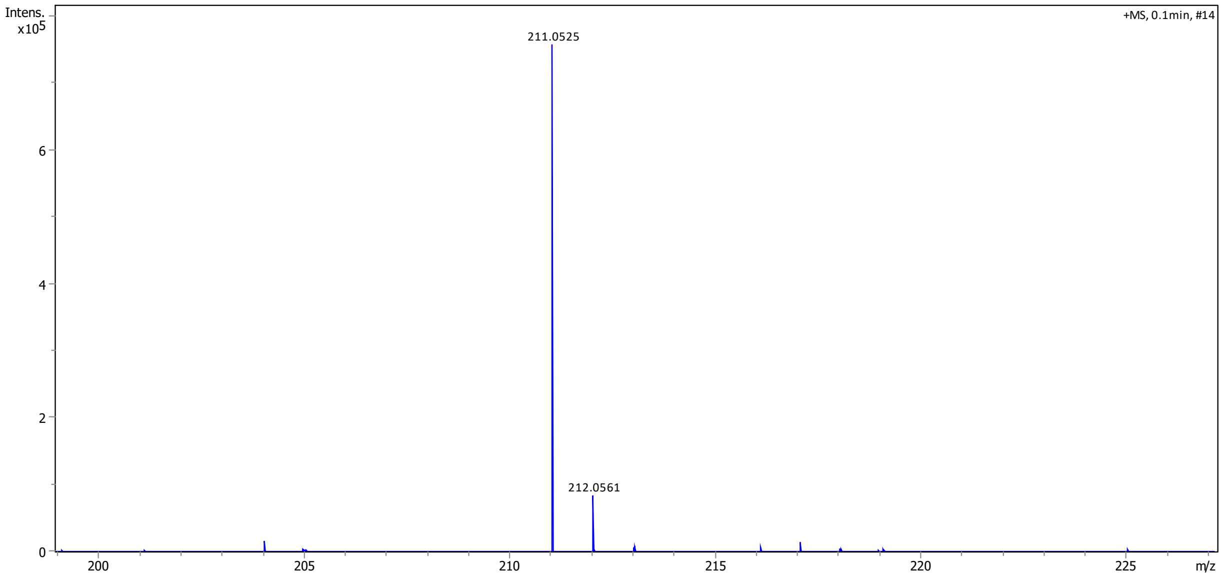

Figure S40. HR ESI–MS spectra of dimethyl(phenylethynyl)phosphonate (**4a**).
HRMS (ESI-TOF) for $C_{10}H_{11}O_3P$ $[M+H]^+$ m/z calcd 211.1688, obsd. 211.0525.

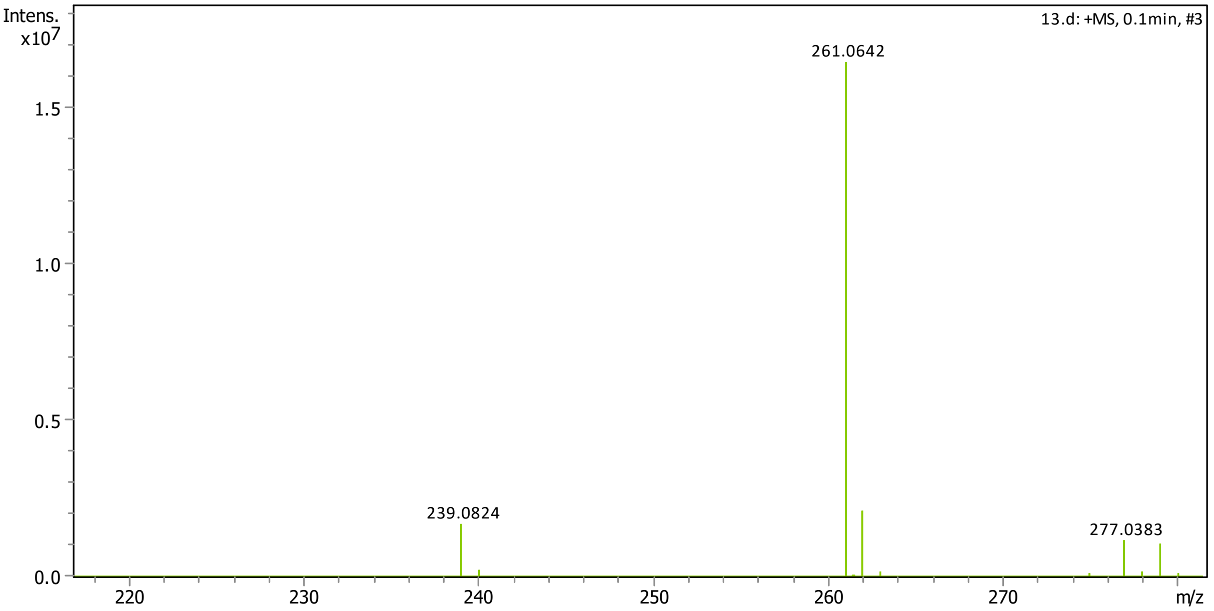

Figure S41. HR ESI–MS spectra of diethyl(phenylethynyl)phosphonate (**4b**).
HRMS (ESI-TOF) for $C_{12}H_{15}O_3P$ $[M+Na]^+$ m/z calcd 261.0656, obsd. 261.0642.

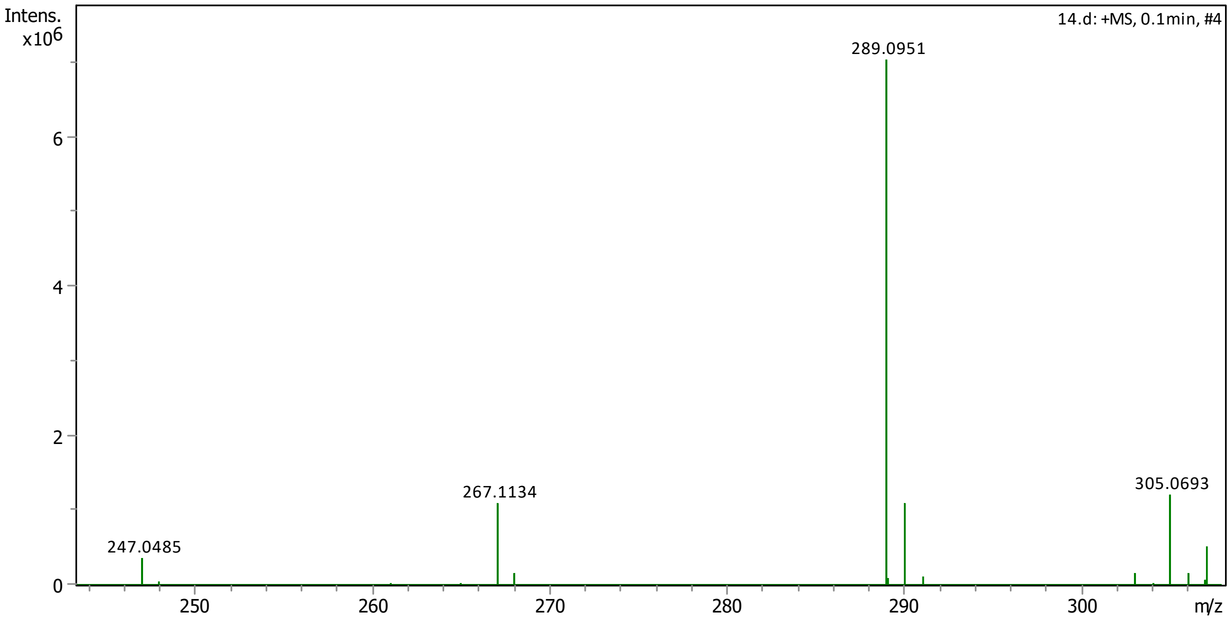

Figure S42. HR ESI–MS spectra of diisopropyl(phenylethynyl)phosphonate (**4c**).
HRMS (ESI-TOF) for $C_{14}H_{19}O_3P$ $[M+Na]^+$ m/z calcd 289.0970, obsd. 289.0951.

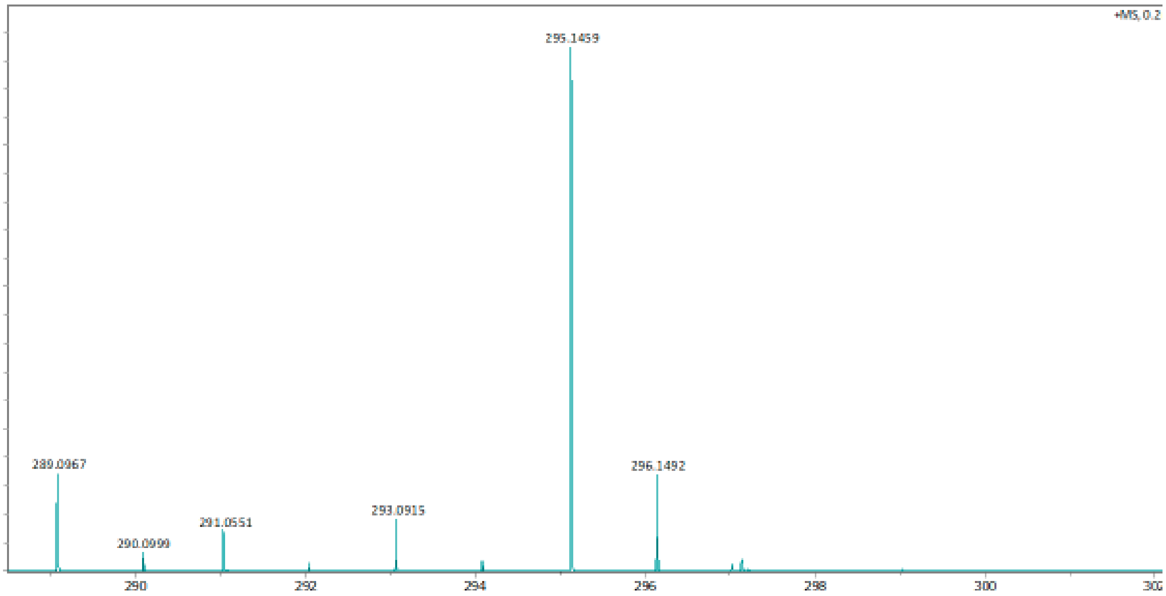

Figure S43. HR ESI–MS spectra of dibutyl(phenylethynyl)phosphonate (**4d**).
HRMS (ESI-TOF) for $C_{16}H_{23}O_3P$ $[M+H]^+$ m/z calcd 295.3359, obsd. 295.1459.

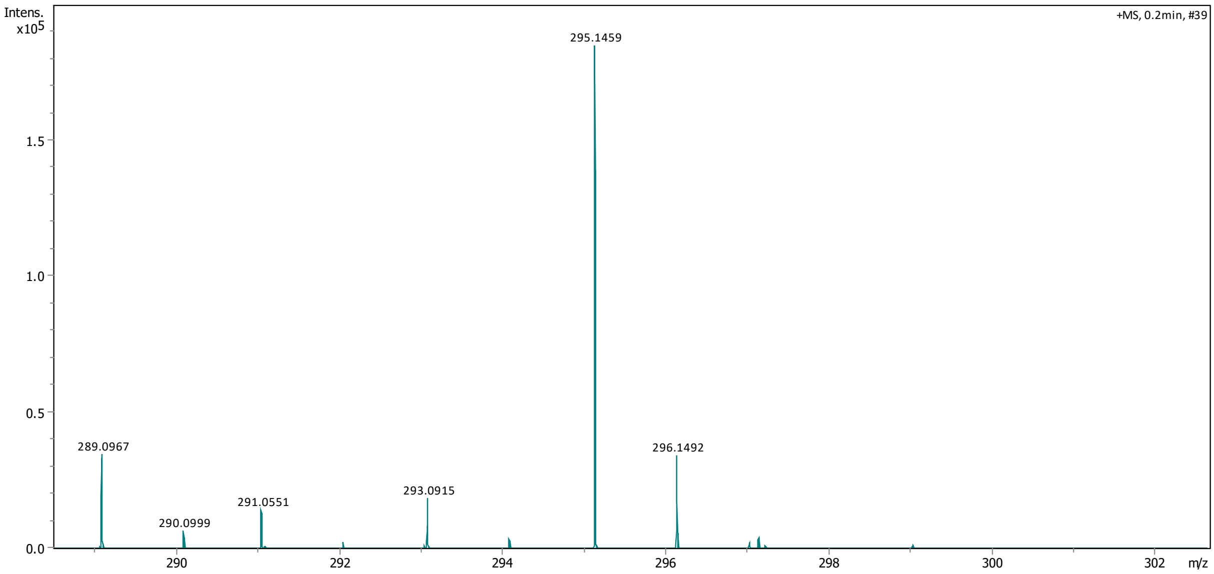

Figure S44. HR ESI–MS spectra of diisobutyl(phenylethynyl)phosphonate(**4e**).
HRMS (ESI-TOF) for $C_{16}H_{23}O_3P$ $[M+H]^+$ m/z calcd 295.3359, obsd. 295.1459.

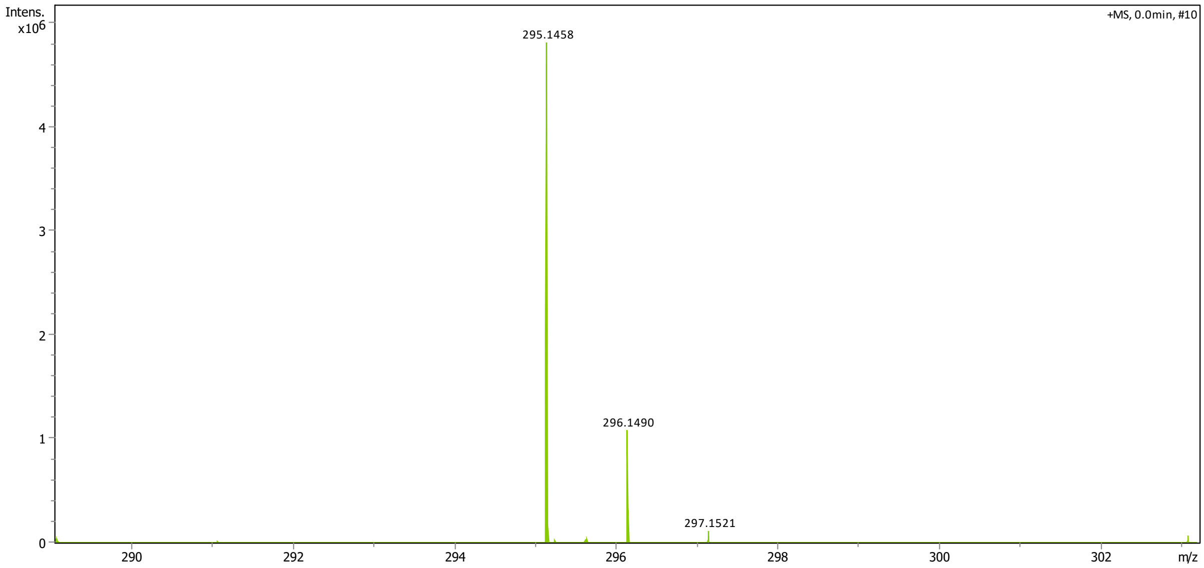

Figure S45. HR ESI–MS spectra of ditret-butyl(phenylethynyl)phosphonate (**4f**).
HRMS (ESI-TOF) for $C_{16}H_{23}O_3P$ $[M+H]^+$ m/z calcd 295.3343, obsd. 295.1458.

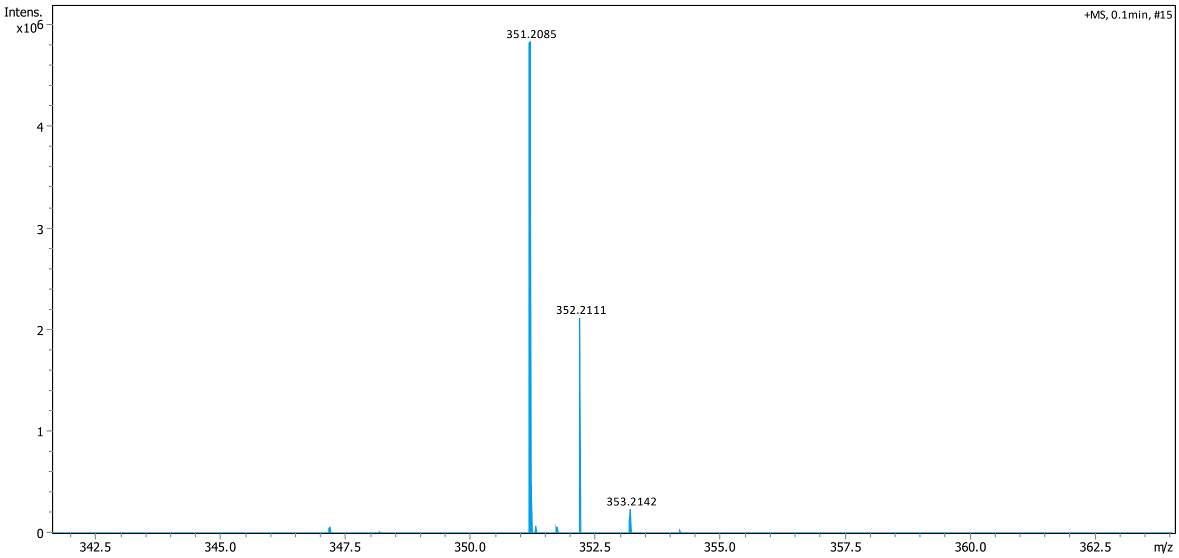

Figure S46. HR ESI–MS spectra of dihexyl(phenylethynyl)phosphonate (**4g**).
HRMS (ESI-TOF) for $C_{20}H_{31}O_3P$ $[M+H]^+$ m/z calcd 351.3301, obsd. 351.2085.

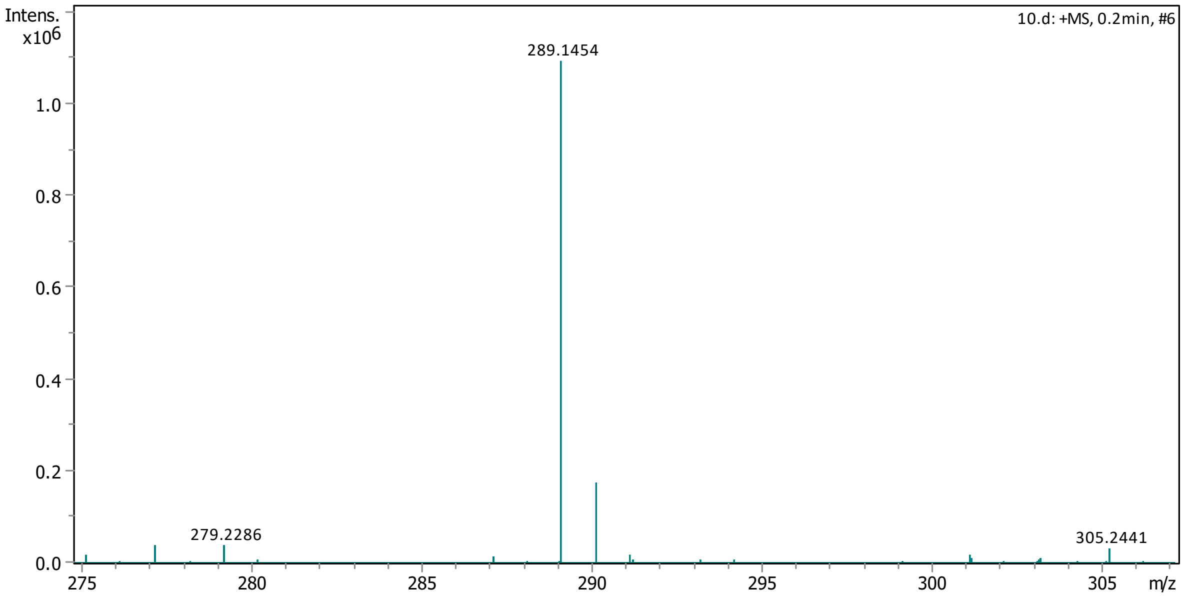

Figure S47. HR ESI–MS spectra of 1,1'-[(phenylethynyl)phosphoryl]dipyrrolidine (**6a**).
HRMS (ESI-TOF) for $C_{16}H_{21}N_2OP$ $[M+H]^+$ m/z calcd 289.1391, obsd. 289.1454.

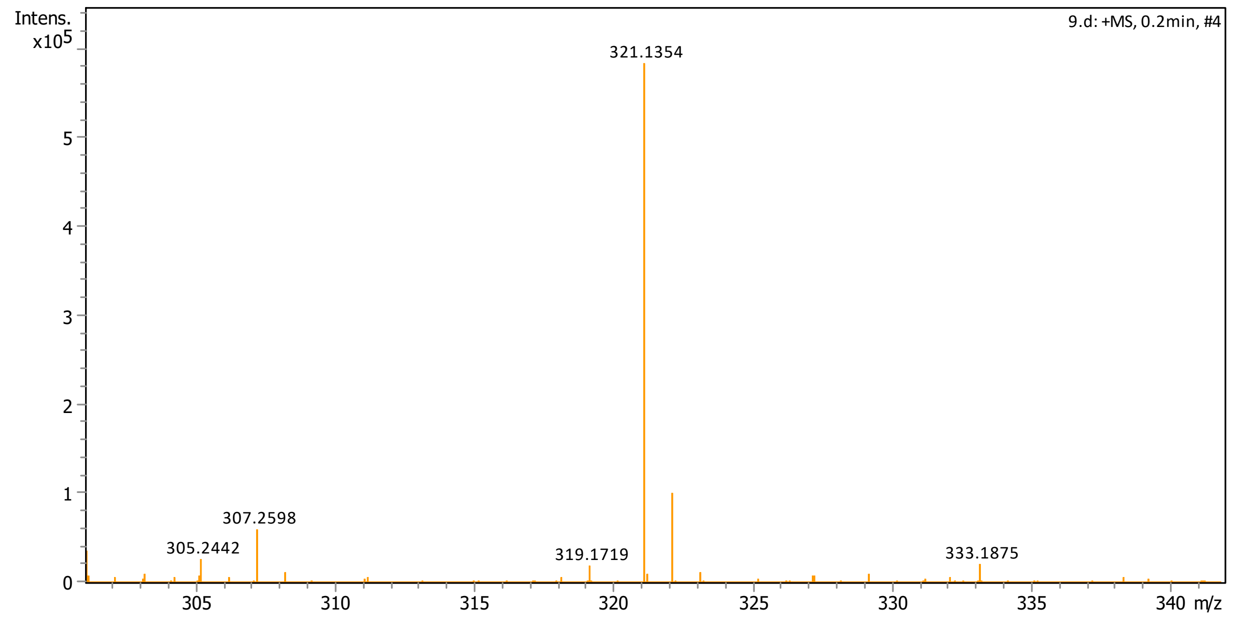

Figure S48. HR ESI–MS spectra of 4,4'-[(phenylethynyl)phosphoryl]bis(morpholine) (**6b**). HRMS (ESI-TOF) for $C_{16}H_{21}N_2O_2P$ $[M+H]^+$ m/z calcd 321.1290, obsd. 321.1354.

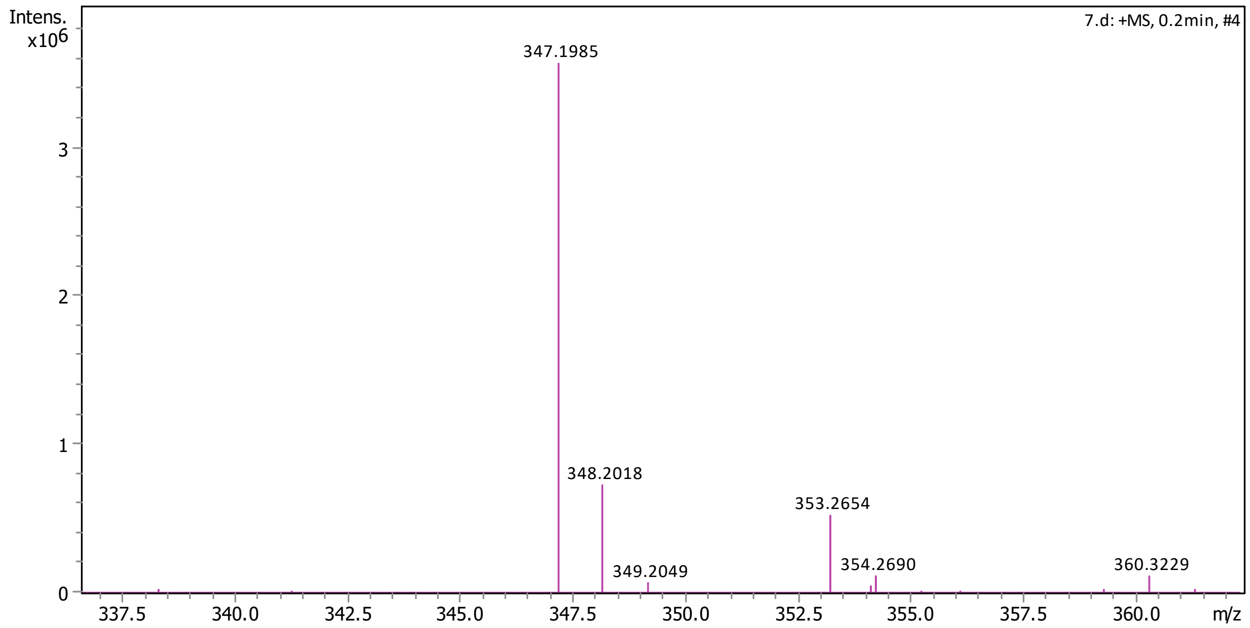

Figure S49. HR ESI–MS spectra of 1,1'-[(phenylethynyl)phosphoryl]dimethylpiperazine (**6c**). HRMS (ESI-TOF) for $C_{18}H_{27}N_4OP$ $[M+H]^+$ m/z calcd 347.1922, obsd. 347.1985.

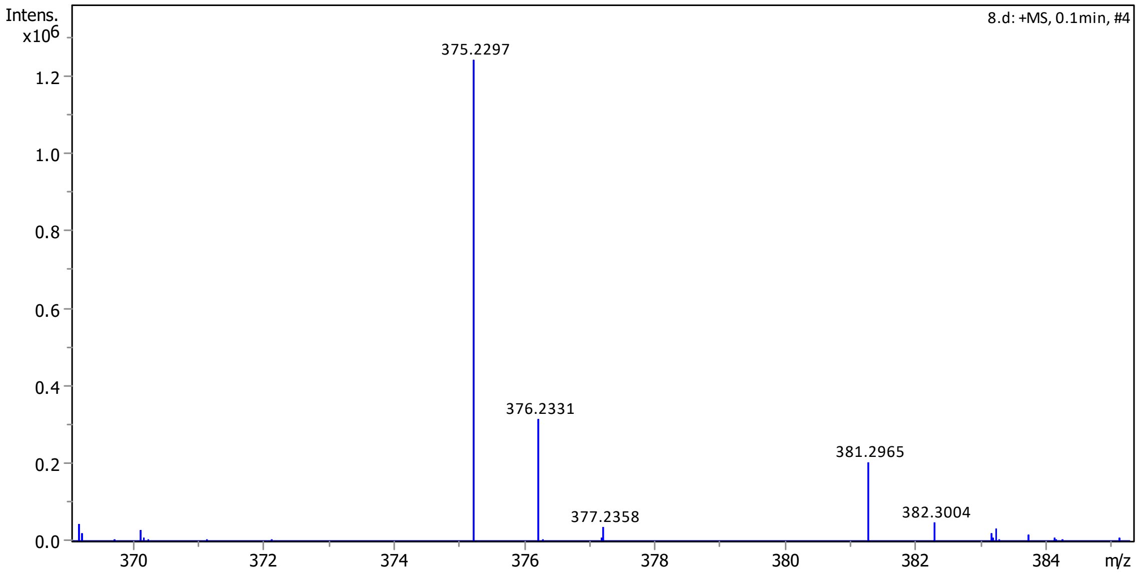

Figure S50. HR ESI–MS spectra of 1,1'-[(phenylethynyl)phosphoryl]diethylpiperazine (**6d**). HRMS (ESI-TOF) for $C_{20}H_{31}N_4OP$ $[M+H]^+$ m/z calcd 375.2235, obsd. 375.2297.

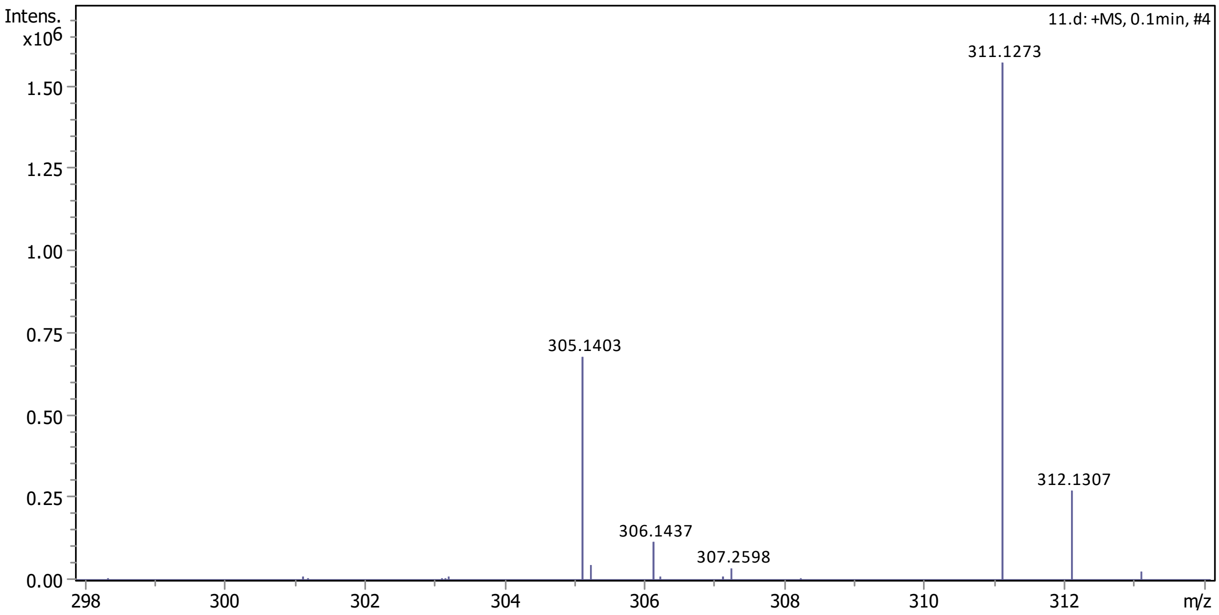

Figure S51. HR ESI–MS spectra of 4-[(phenylethynyl)(pyrrolidin-1-yl)phosphoryl]morpholine (**6e**). (ESI-TOF) for $C_{16}H_{21}N_2O_2P$ $[M+H]^+$ m/z calcd 305.1341, obsd. 305.1403.

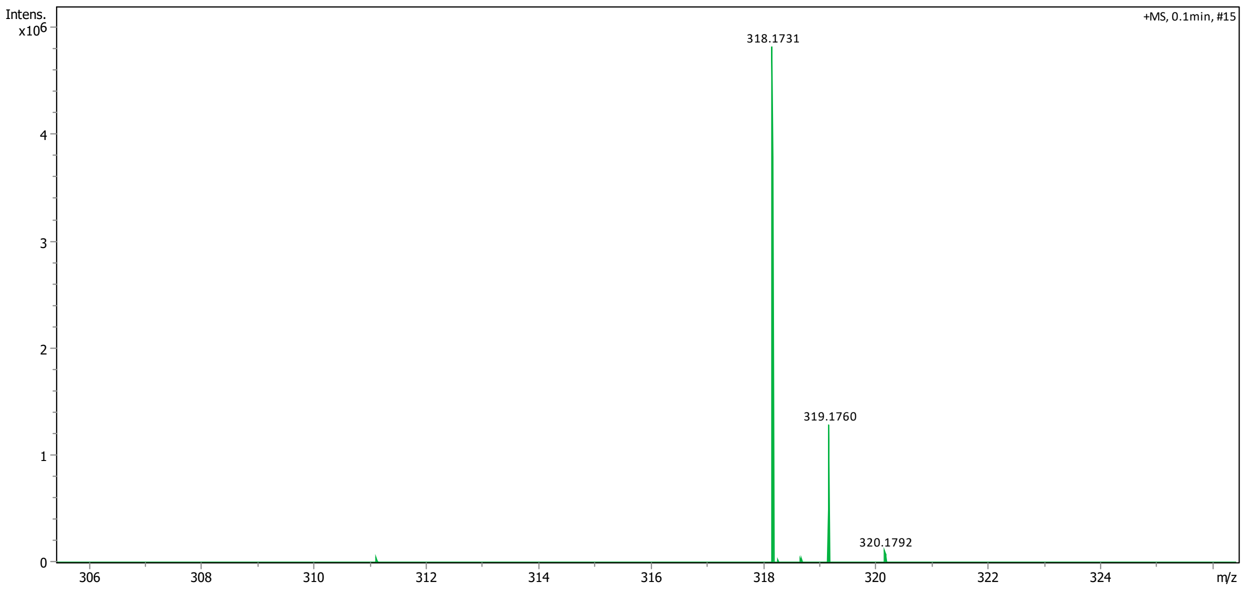

Figure S52. HR ESI–MS spectra of 4-[(phenylethynyl)(pyrrolidin-1-yl)phosphoryl]1-methylpiperazine (**6f**).

HRMS (ESI-TOF) for $C_{17}H_{24}N_3OP$ $[M+H]^+$ m/z calcd 318.1657, obsd. 318.1701.

4. HPLC analysis

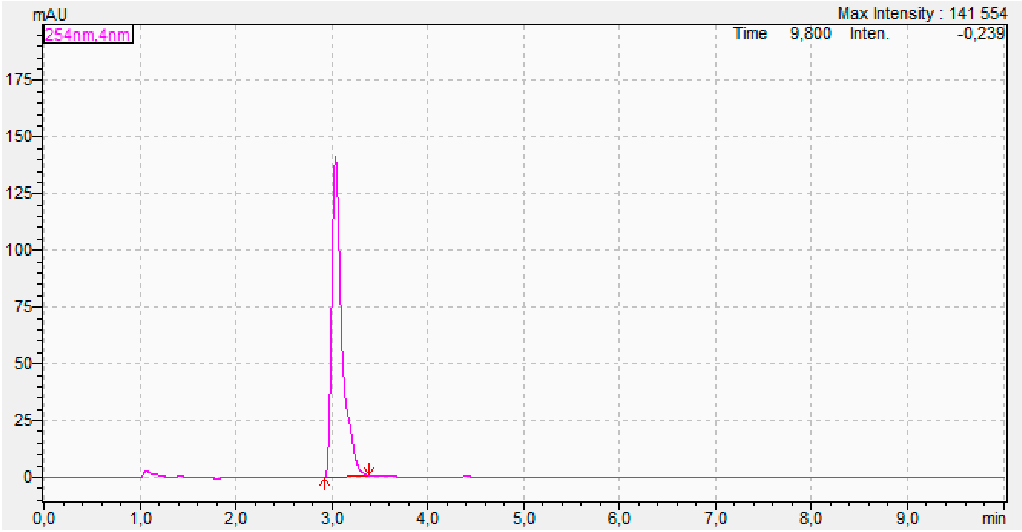

Figure S53. Chromatogram of dimethyl(phenylethynyl)phosphonate (**4a**).

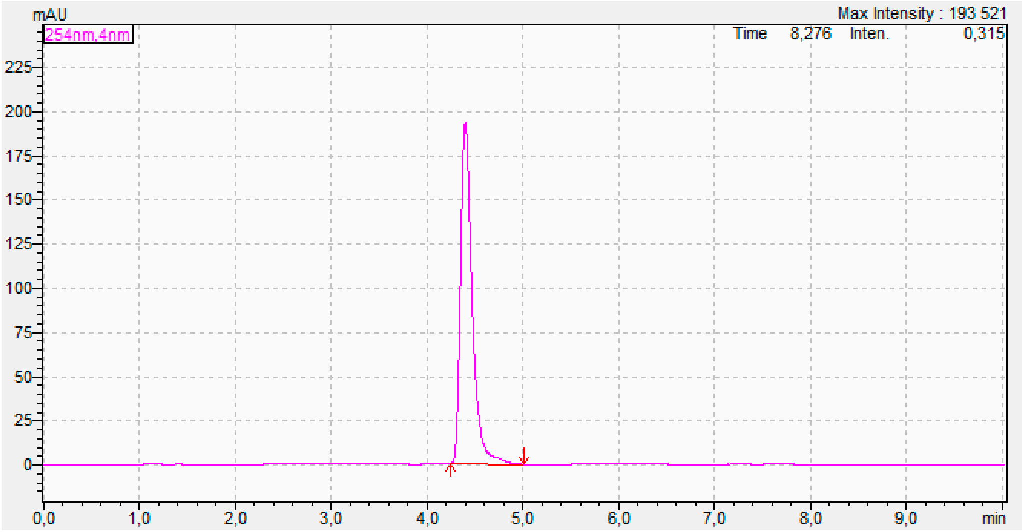

Figure S54. Chromatogram of diethyl(phenylethynyl)phosphonate (**4b**).

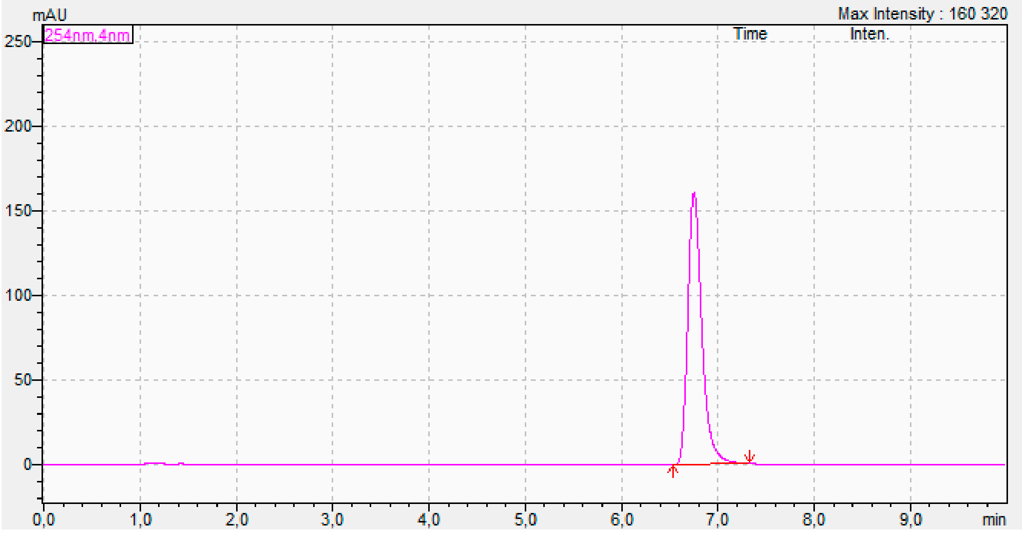

Figure S55. Chromatogram of diisopropyl(phenylethynyl)phosphonate (**4c**).

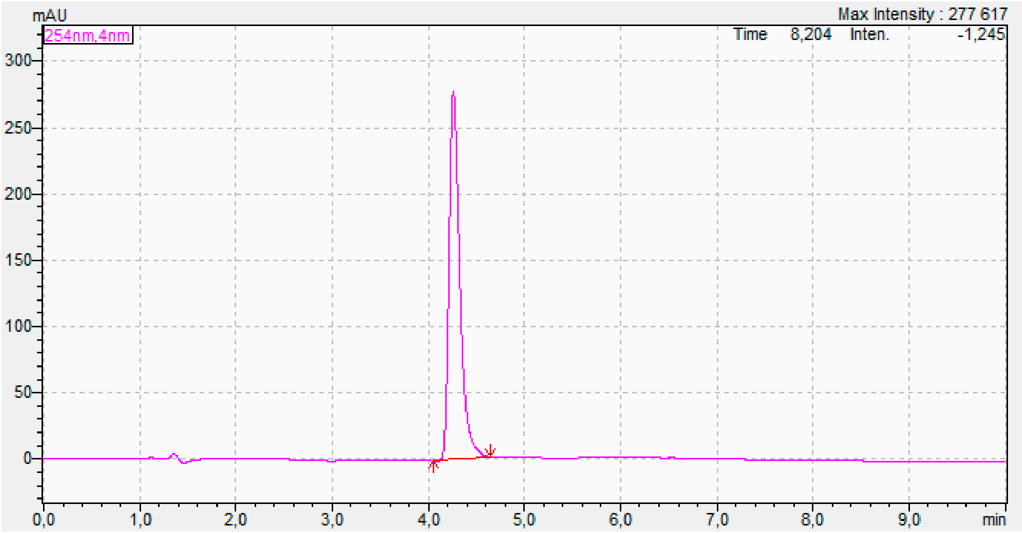

Figure S56. Chromatogram of dibutyl(phenylethynyl)phosphonate (**4d**).

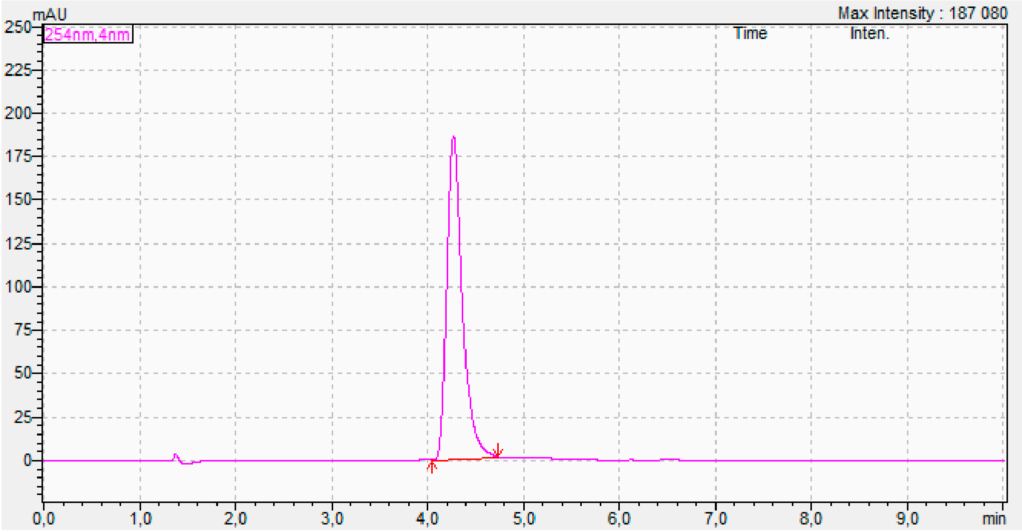

Figure S57. Chromatogram of diisobutyl(phenylethynyl)phosphonate (**4e**).

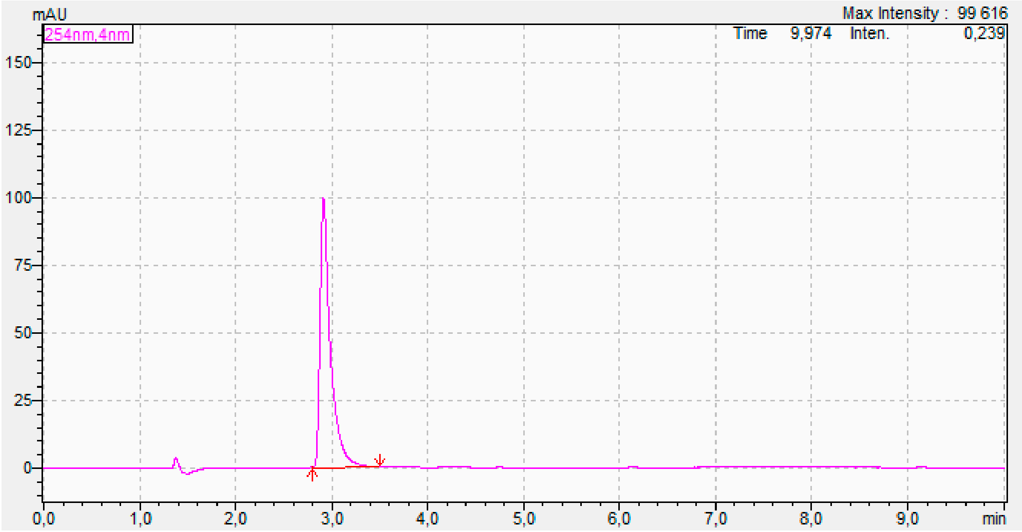

Figure S58. Chromatogram of ditret-butyl(phenylethynyl)phosphonate (**4f**).

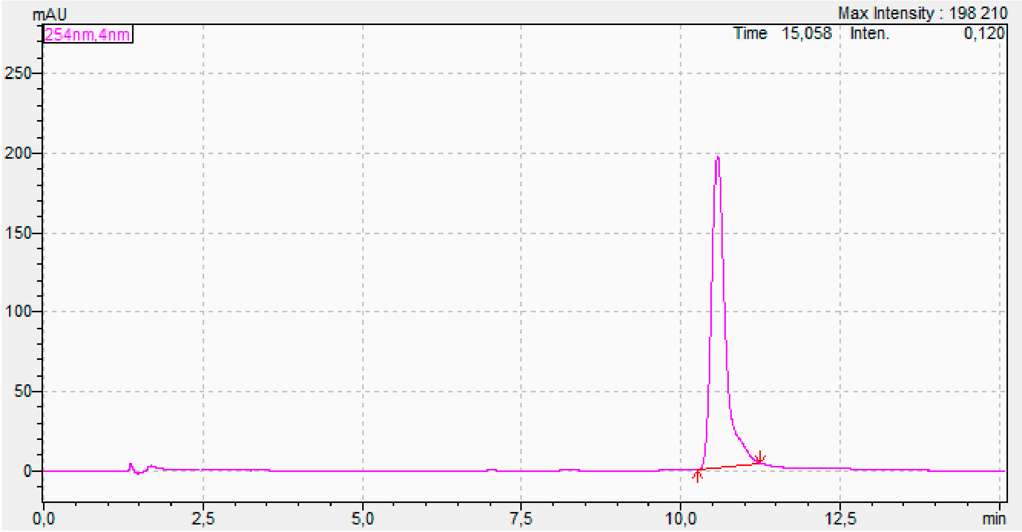

Figure S59. Chromatogram of dihexyl(phenylethynyl)phosphonate (**4g**).

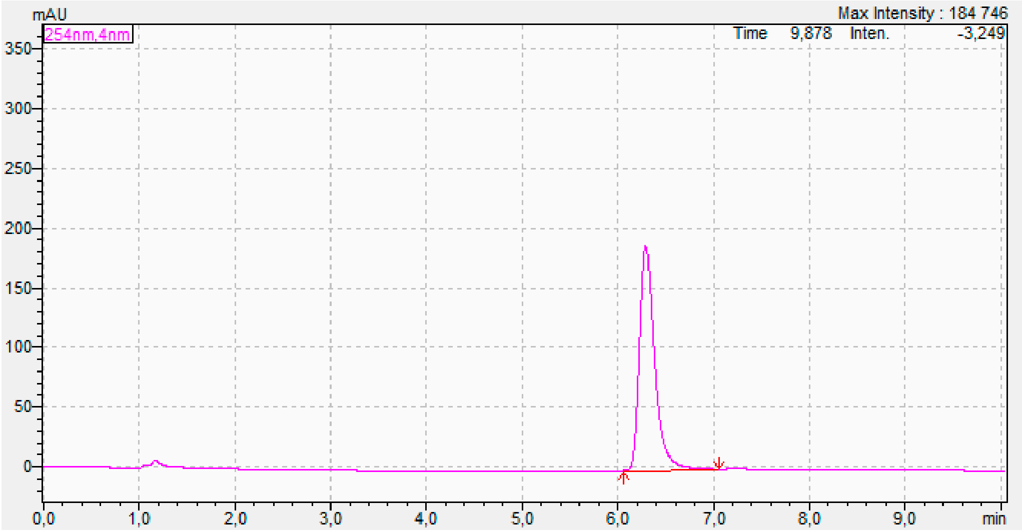

Figure S60. Chromatogram of 1,1'-[(phenylethynyl)phosphoryl]dipyrrolidine (**6a**).

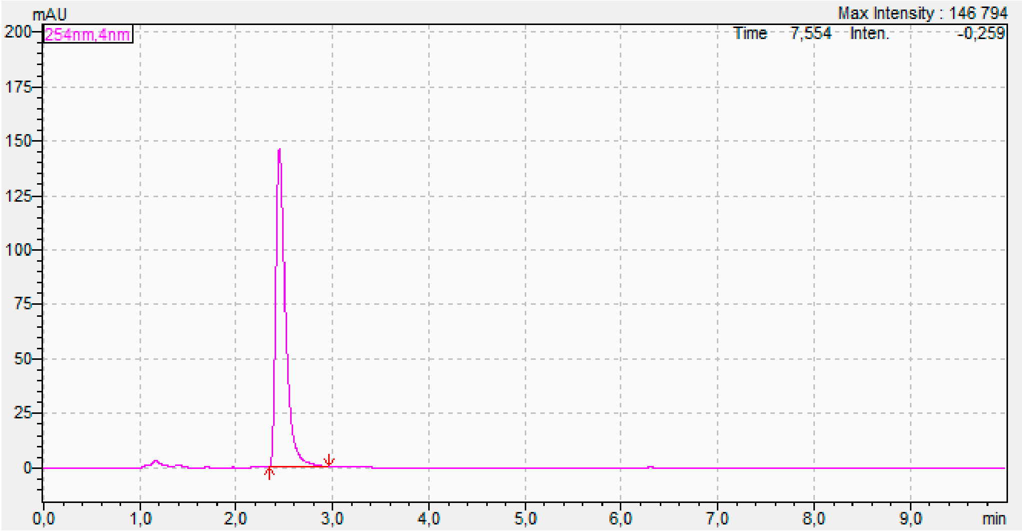

Figure S61. Chromatogram of 4,4'-[(phenylethynyl)phosphoryl]bis(morpholine) (**6b**).

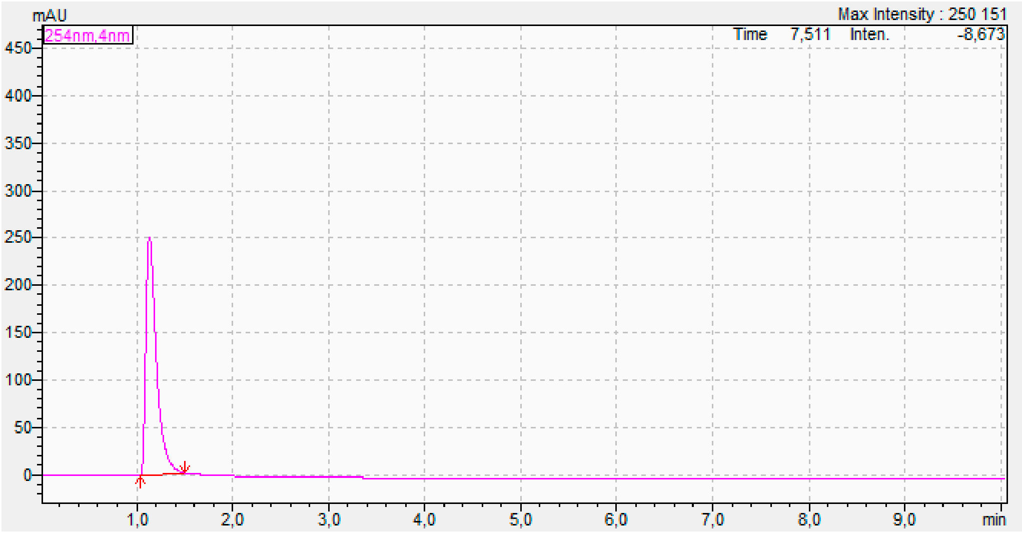

Figure S62. Chromatogram of 1,1'-[(phenylethynyl)phosphoryl]dimethylpiperazine (**6c**).

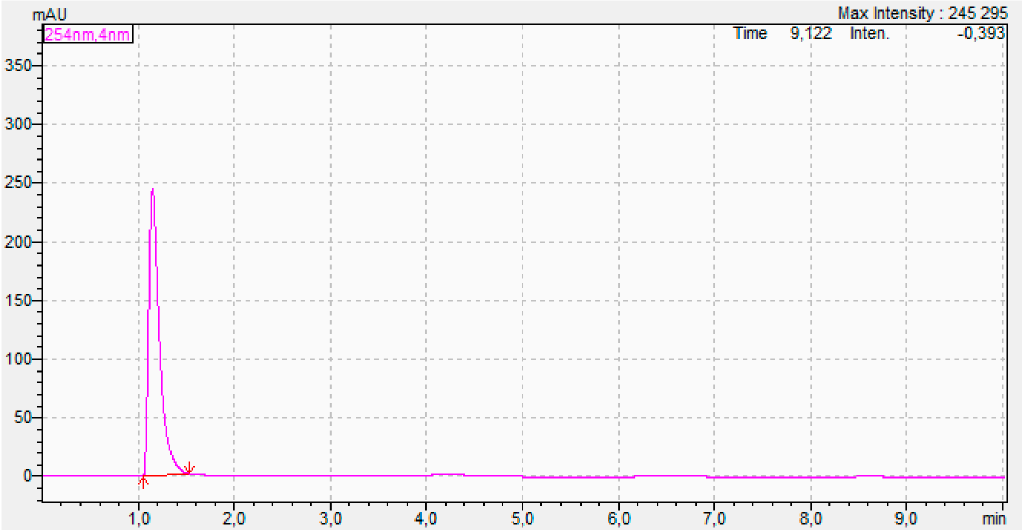

Figure S63. Chromatogram of 1,1'-[(phenylethynyl)phosphoryl]diethylpiperazine (**6d**).

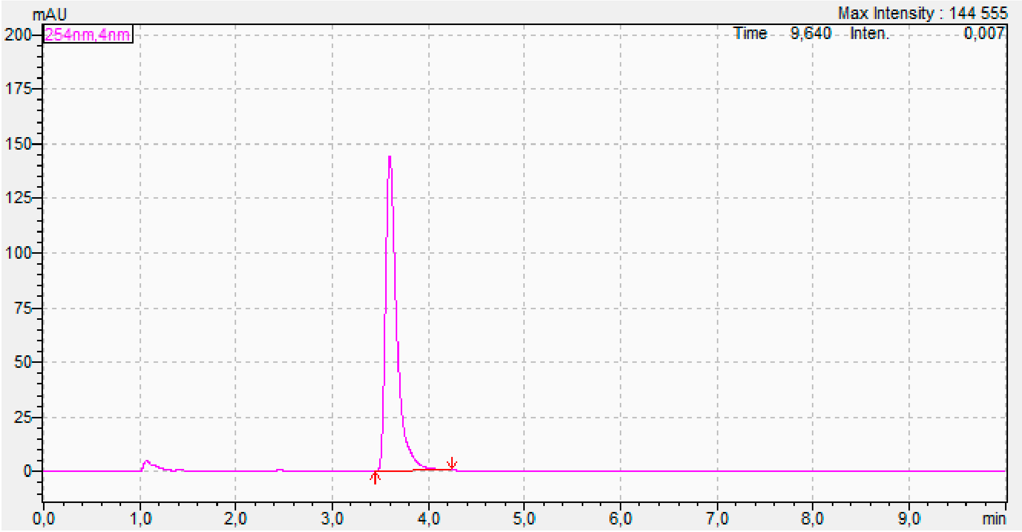

Figure S64. Chromatogram of 4-[(phenylethynyl)(pyrrolidin-1-yl)phosphoryl]morpholine (**6e**).

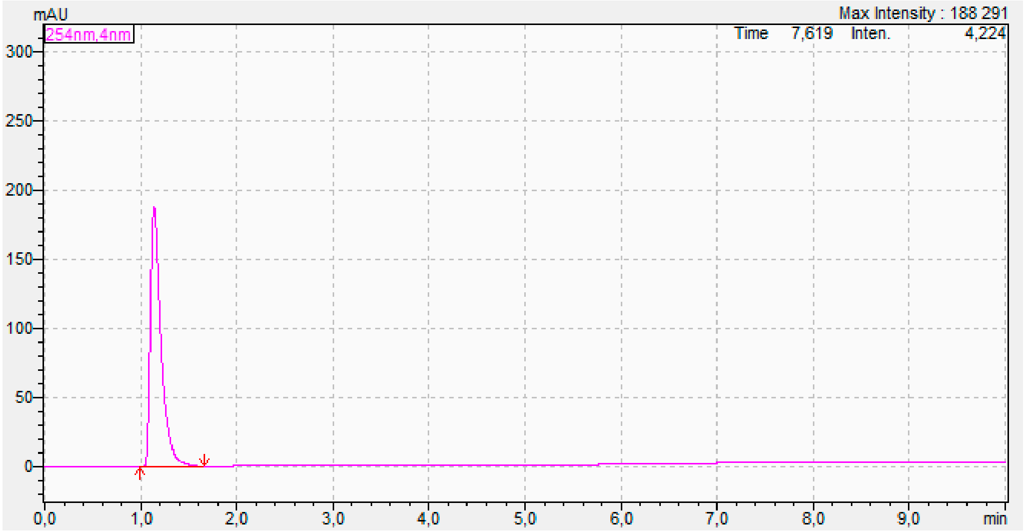

Figure S65. Chromatogram of 4-[(phenylethynyl)(pyrrolidin-1-yl)phosphoryl]1-methylpiperazine (**6f**).

Table S8. Retention time of compounds and chromatographic conditions.

No.	Compound	Retention time, min	Eluent
1	**4a**	3.040	MeCN–0.1% TFA, 50:50
2	**4b**	4.397	MeCN–0.1% TFA, 50:50
3	**4c**	6.752	MeCN–0.1% TFA, 50:50
4	**4d**	4.263	MeCN–0.1% TFA, 70:30
5	**4e**	4.268	MeCN–H_2O, 70:30
6	**4f**	2.916	MeCN–H_2O, 70:30
7	**4g**	10.578	MeCN–H_2O, 70:30
8	**6a**	6.293	MeCN–0.1% TFA, 50:50
9	**6b**	2.450	MeCN–0.1% TFA, 50:50
10	**6c**	1.135	MeCN–0.1% TFA, 40:60
11	**6d**	1.149	MeCN–0.1% TFA, 40:60
12	**6e**	3.601	MeCN–0.1% TFA, 50:50
13	**6f**	1.136	MeCN–0.1% TFA, 40:60

5. Wound-healing ability (scratch-test)

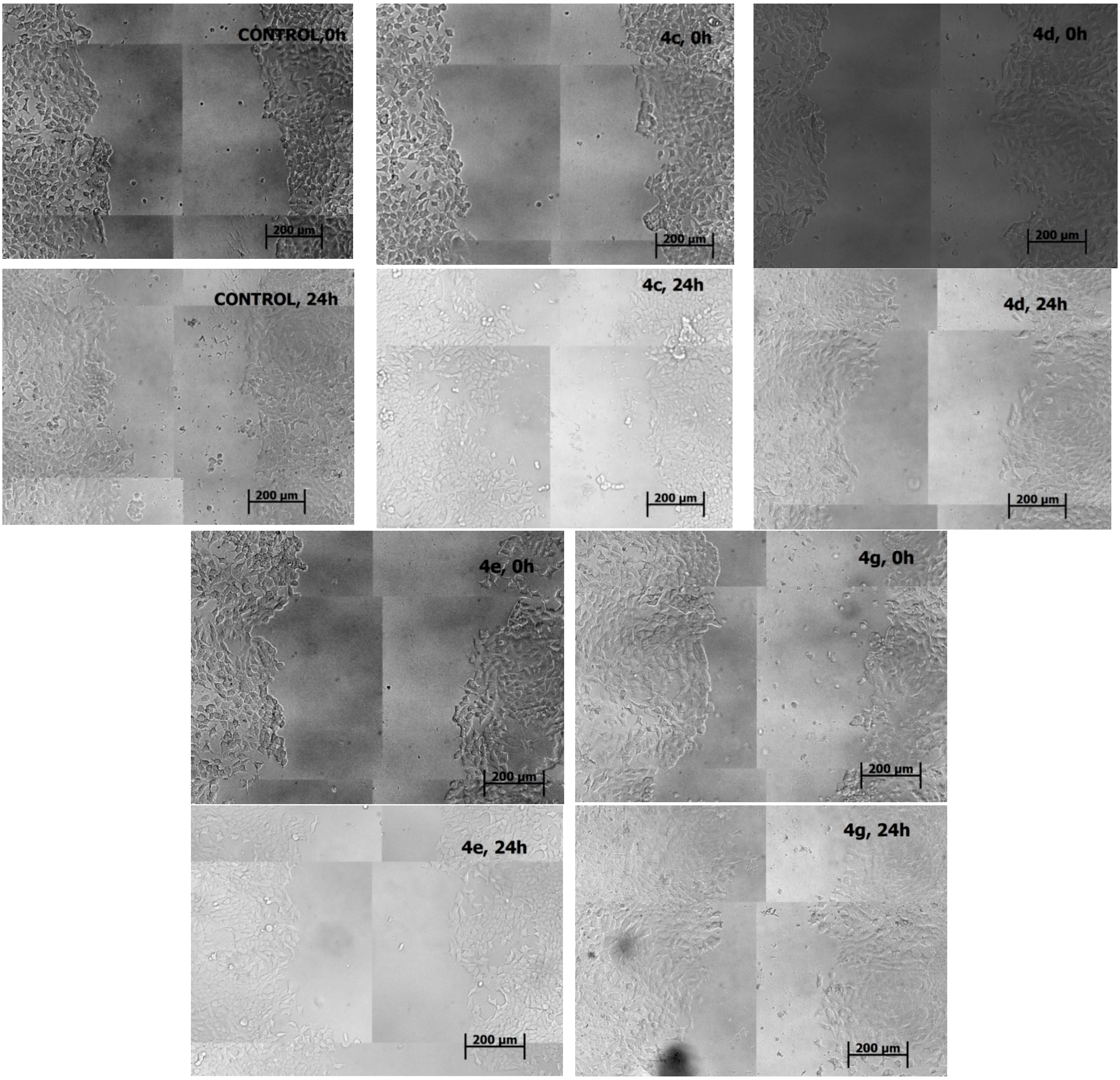

Figure S66. Wound-healing ability of Sk-mel-2 cells after incubation with compounds **4c**, **4d**, **4e**, and **4g**. p-value < 0.05 (*), 0.001 (****).

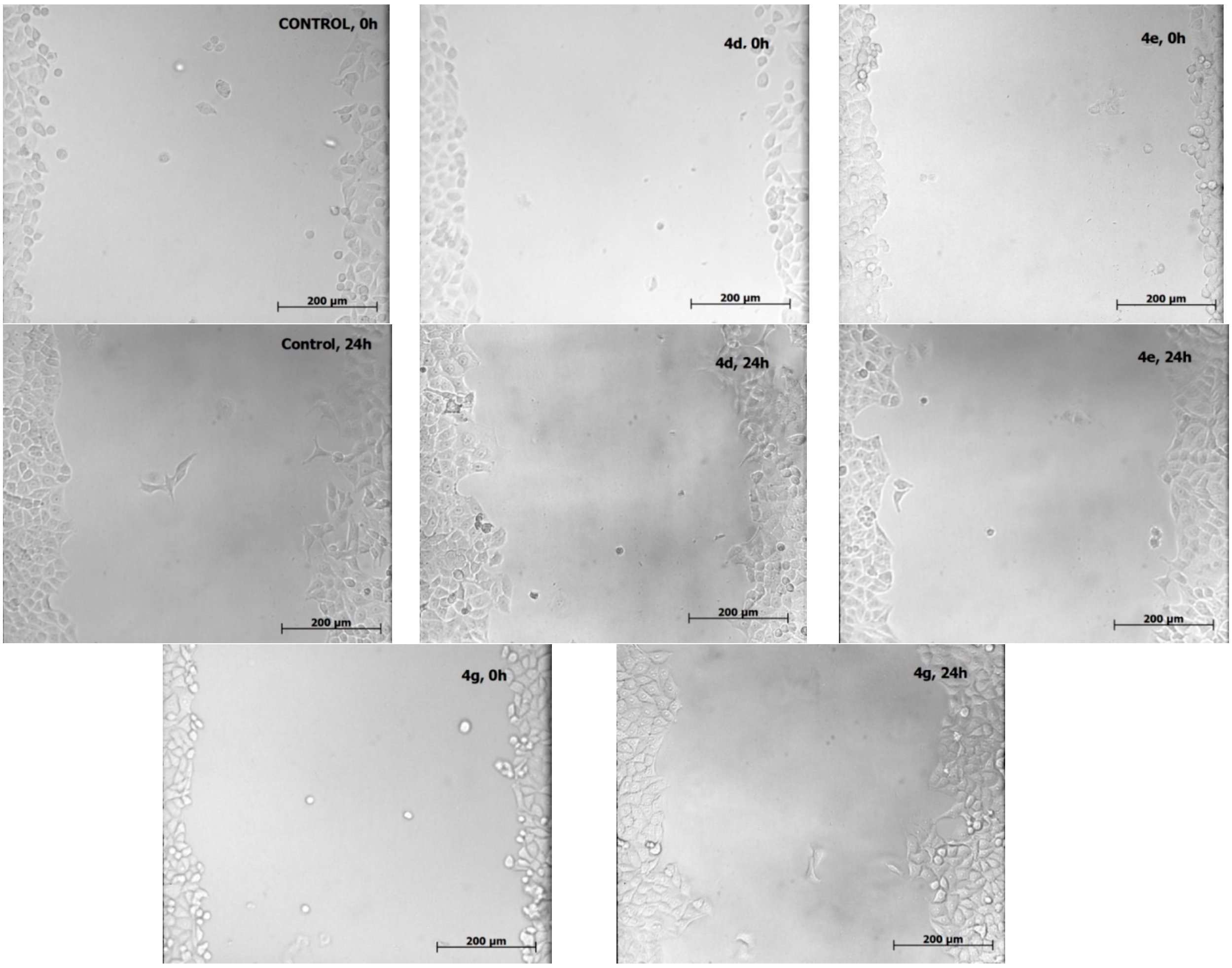

Figure S67. Wound-healing ability of HeLa cells after incubation with compounds **4d**, **4e**, and **4g**. p-value < 0.001 (****).

6. ADMET prediction

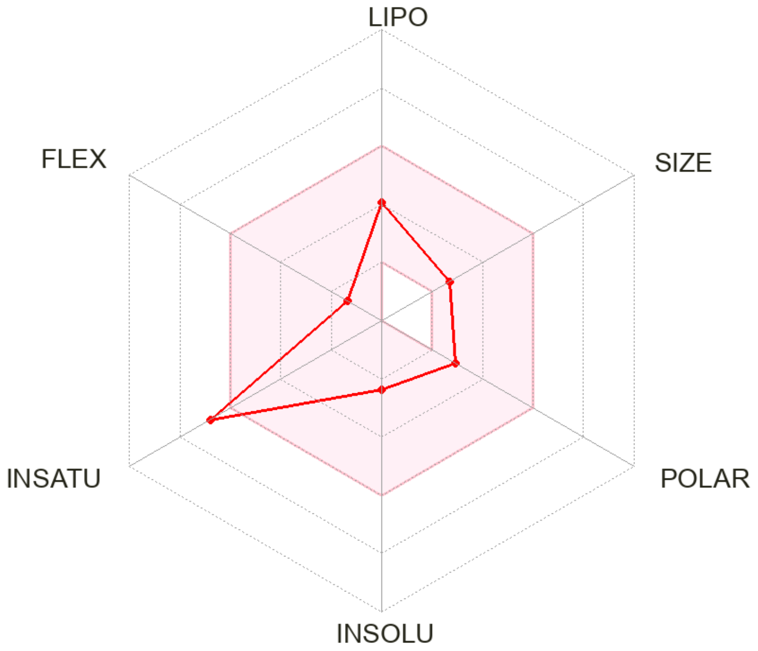

Figure S68. The Bioavailability Radar for dimethyl(phenylethynyl)phosphonate (**4a**).

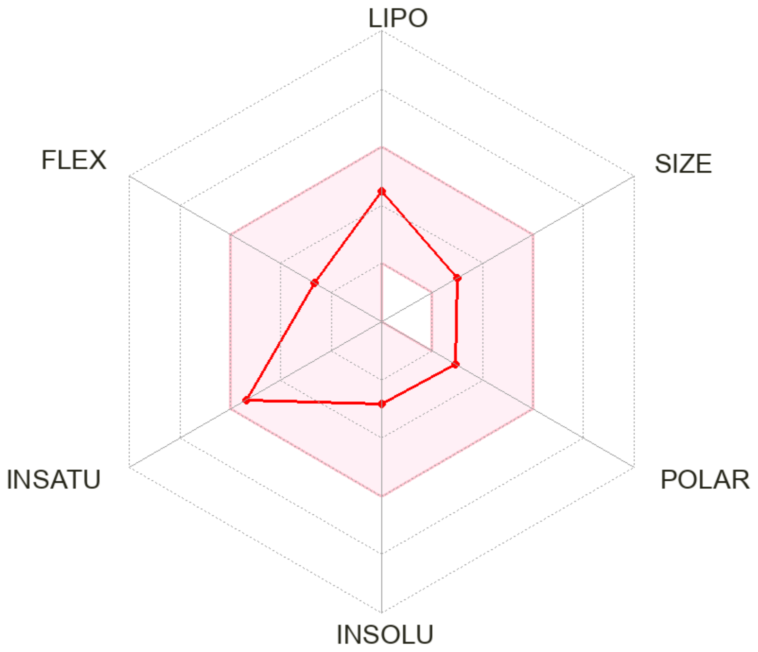

Figure S69. The Bioavailability Radar for diethyl(phenylethynyl)phosphonate (**4b**).

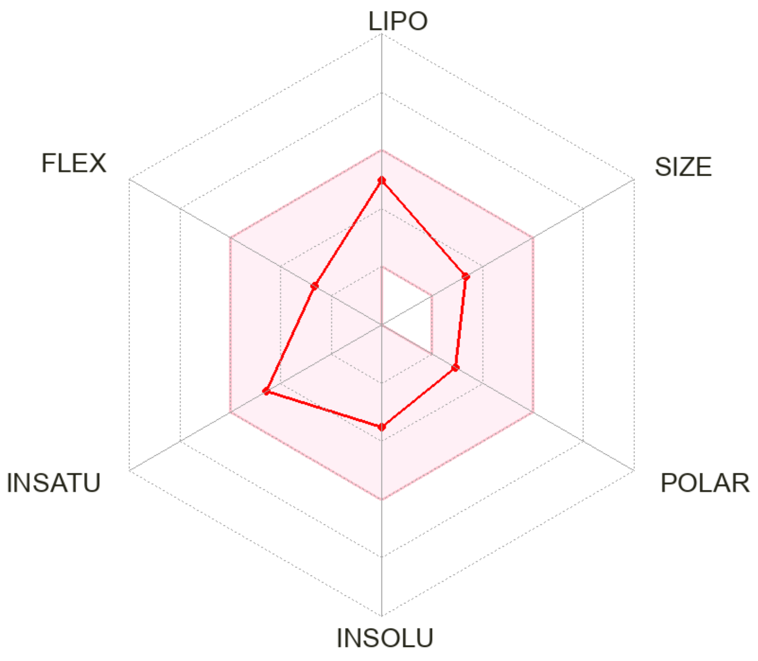

Figure S70. The Bioavailability Radar for diisopropyl(phenylethynyl)phosphonate (**4c**).

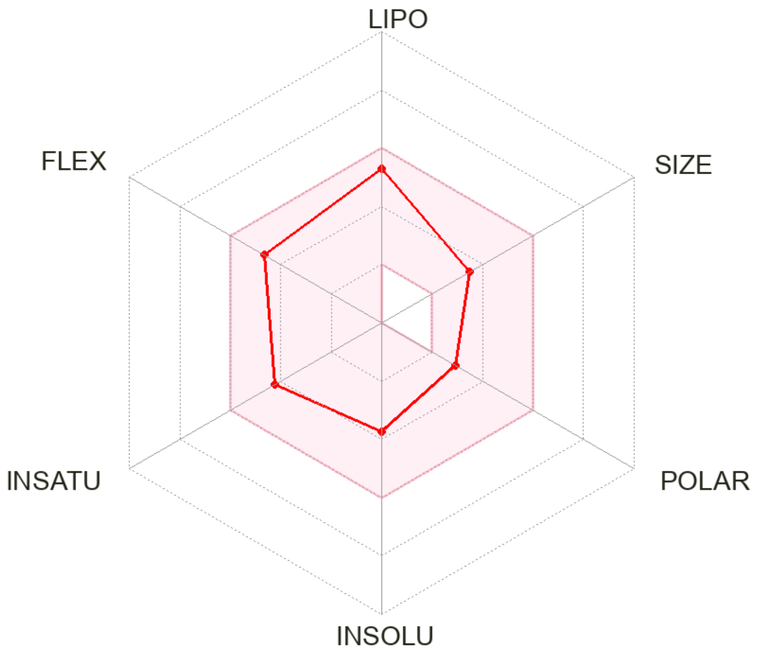

Figure S71. The Bioavailability Radar for dibutyl(phenylethynyl)phosphonate (**4d**).

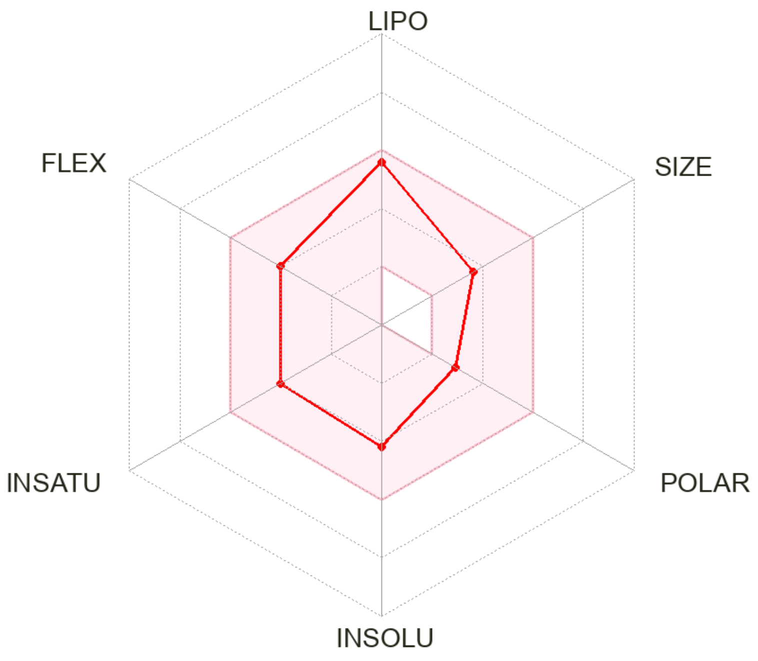

Figure S72. The Bioavailability Radar for diisobutyl(phenylethynyl)phosphonate (**4e**).

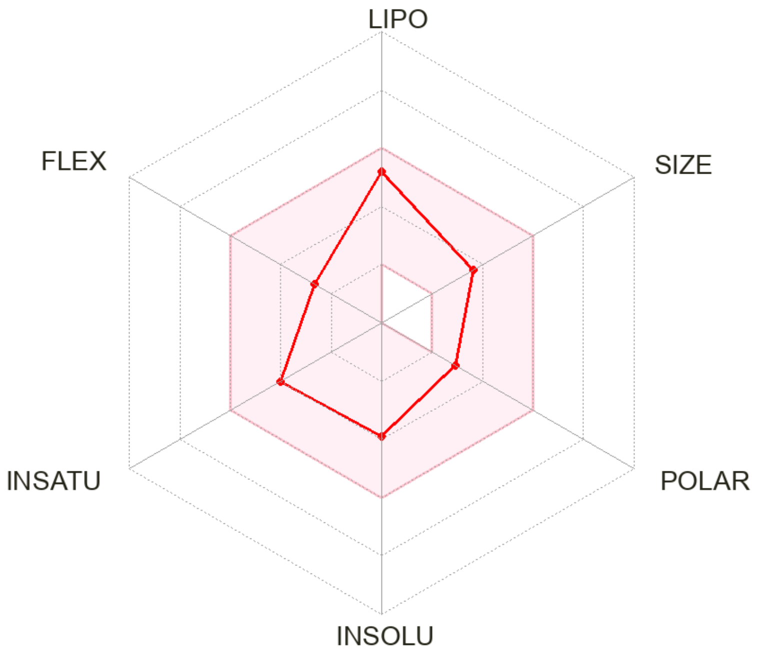

Figure S73. The Bioavailability Radar for ditret-butyl(phenylethynyl)phosphonate (**4f**).

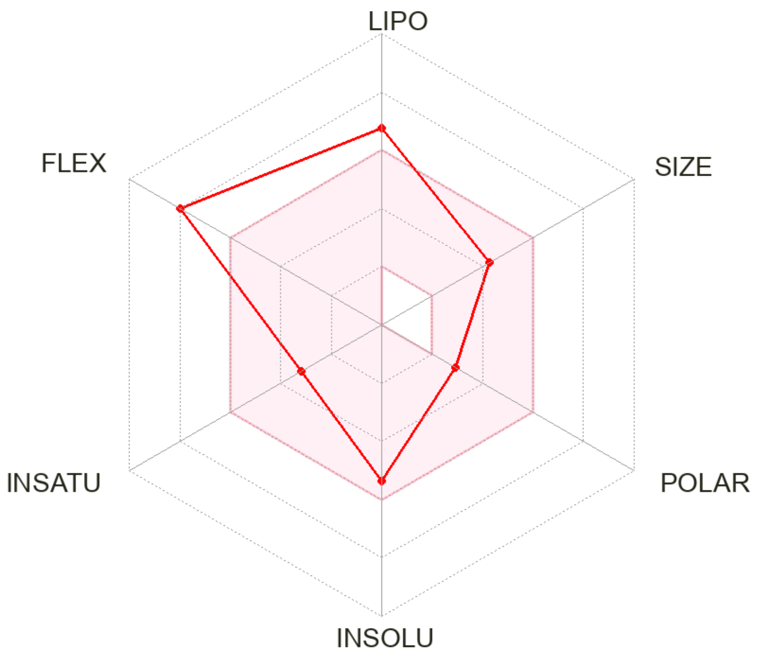

Figure S74. The Bioavailability Radar for dihexyl(phenylethynyl)phosphonate (**4g**).

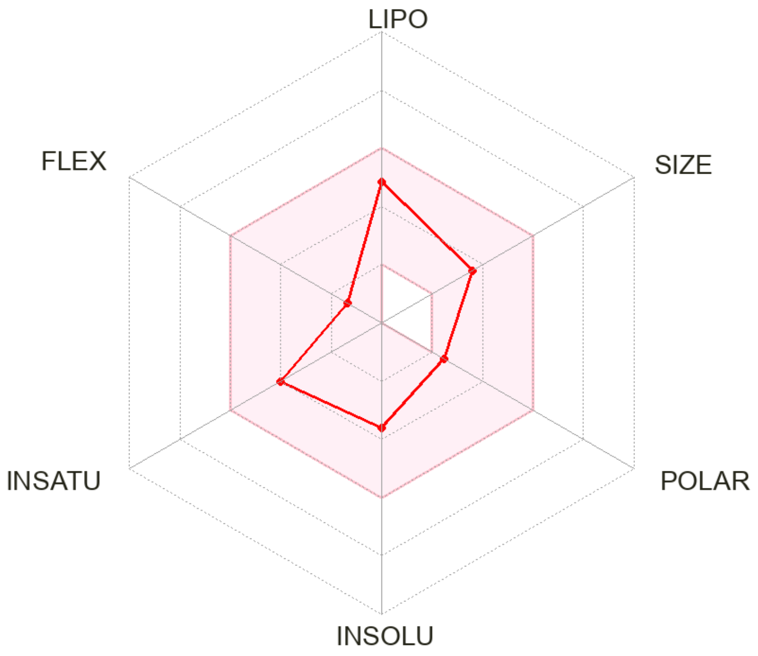

Figure S75. The Bioavailability Radar for 1,1'-[(phenylethynyl)phosphoryl]dipyrrolidine (**6a**).

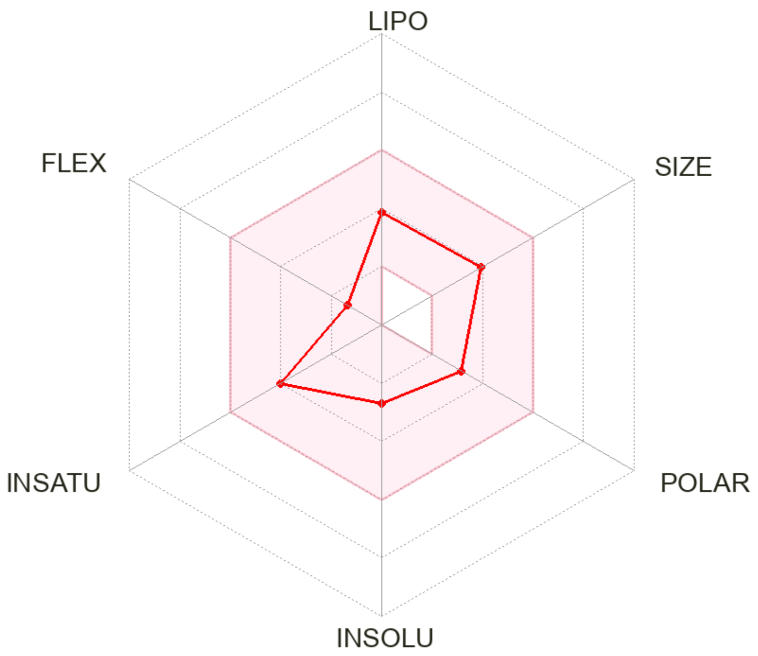

Figure S76. The Bioavailability Radar for 4,4'-[(phenylethynyl)phosphoryl]bis(morpholine) (**6b**).

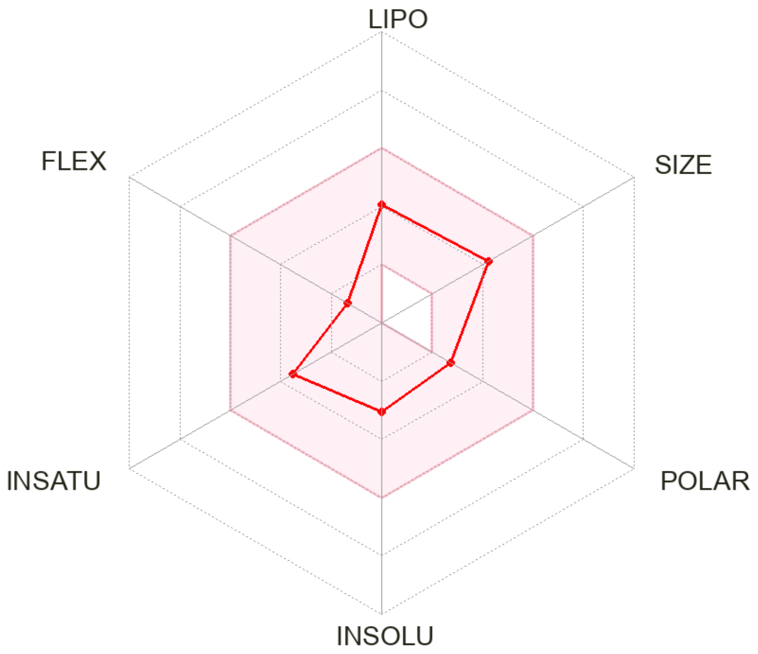

Figure S77. The Bioavailability Radar for 1,1'-[(phenylethynyl)phosphoryl]dimethylpiperazine (**6c**).

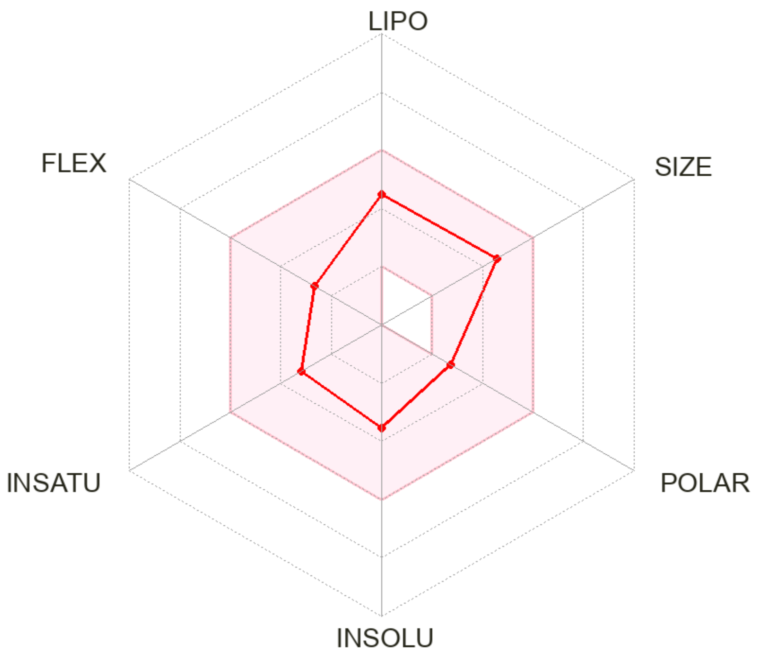

Figure S78. The Bioavailability Radar for 1,1'-[(phenylethynyl)phosphoryl]diethylpiperazine (**6d**).

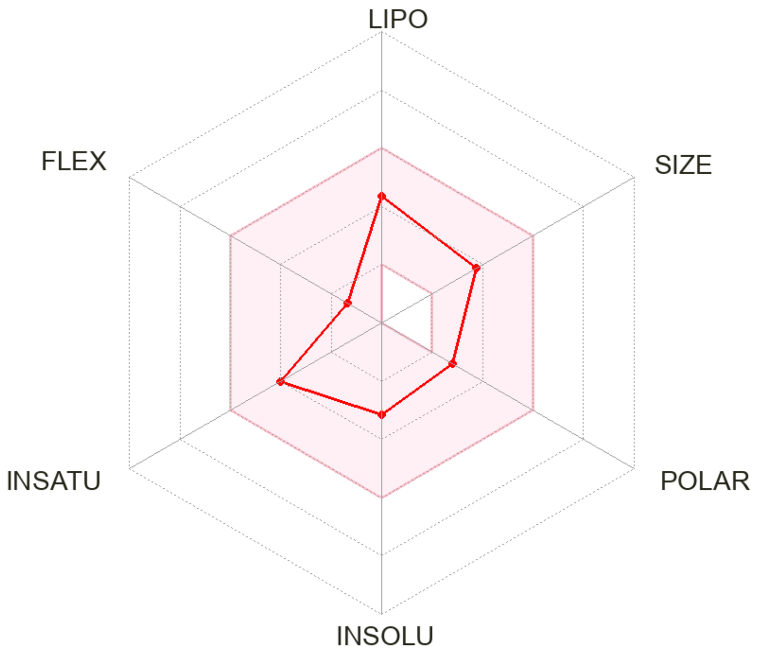

Figure S79. The Bioavailability Radar for 4-[(phenylethynyl)(pyrrolidin-1-yl)phosphoryl]morpholine (**6e**).

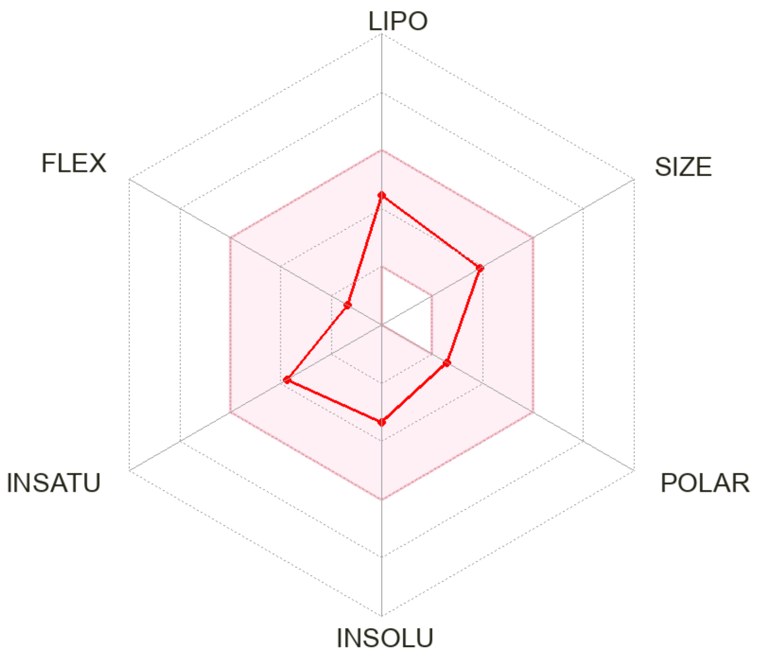

Figure S80. The Bioavailability Radar for 4-[(phenylethynyl)(pyrrolidin-1-yl)phosphoryl]1-methylpiperazine (**6f**).

7. Molecular docking

Table S9. Docking Score values for redocking of the ligand to the corcystalized protein model.

No.	Target	Model	Ligand	Docking Score, kcal/mol
1	EGFR	4HJO	-	-
2	PDGFRA	6JOL	-	-
3	B-RAF	5CSW	-	-
4	FAK	2IJM	-	-
5	AMPK	7JHG	Compound C	–8.7
6	DYRK1A	7O7K	abemaciclib	–11.2
7	DYRK1B	8C2Z	AZ191	–12.5
8	JAK1	6N7A	-	-
9	JAK2	8EX1	reversin	–9.5
10	HIPK2	6P5S	silmitasertib	–10.7
11	HIPK3	7O7J	abemaciclib	–10.4
12	CDK4	7SJ3	abemaciclib	–11.5
13	CDK7	8S0T	SY-5609	
14	CSNK2A1	3PE1	silmitasertib	–11.3
15	CSNK2A2	6HMB	silmitasertib	–11.4
16	MYLK4	2X4F	16X	–9.9
17	HASPIN	3IQ7	5-iodotubercidin	–6.5
18	PIM1	4XHK	Compound 1s	–8.9
19	PIM2	4X7Q	Compound 1s	–8.8
20	TYK2	6NZP	deucravacitinib	–9.9
21	AURKB	4AF3	VX6	–9.2
22	PDE5	4MD6	inhibitor 5R	–5.7
23	mGluR3	8TR0	LY-341495	–7.5
24	MDR1	7A65	Compound C	–7.8
25	PARP1	7KK4	olaparib	–12.5
26	PARP2	4TVJ	olaparib	–12.7
27	PARP3	4GV0	ME0355	–10.3
28	TGF-β	1PY5	-	-
29	HA	3EYM	TBHC	–6.0
30	RdRp	6LD3	G8O-FB2	–6.8
31	MPro	6LU7	-	-
32	Actin	8DNH	ADP	–7.6
